# Association between Dietary Indices and Dietary Patterns and Mortality and Cancer Recurrence among Cancer Survivors: An Updated Systematic Review and Meta-Analysis of Cohort Studies

**DOI:** 10.3390/nu15143151

**Published:** 2023-07-14

**Authors:** Angela Trauchburg, Lukas Schwingshackl, Georg Hoffmann

**Affiliations:** 1 Department of Nutritional Sciences, Faculty of Life Sciences, University of Vienna, Josef-Holaubek-Platz 2, UZA II, 1090 Vienna, Austria; a01446284@unet.univie.ac.at; 2 Institute for Evidence in Medicine, Medical Center, Faculty of Medicine, University of Freiburg, Breisacher Straße 153, 79110 Freiburg, Germany; lukas.schwingshackl@uniklinik-freiburg.de

**Keywords:** cancer survivors, dietary patterns, diet-quality indices, nutrition, overall mortality, cancer-specific mortality, cancer recurrence

## Abstract

The number of cancer survivors is growing rapidly; however, specific lifestyle recommendations for these patients are still sparse, including dietary approaches. Thus, the aim of the present systematic review and meta-analysis was to examine the associations between adherence to diet-quality indices and dietary patterns on overall mortality, cancer-specific mortality, and cancer recurrence among cancer survivors. The literature search was conducted in PubMed and Web of Science between 18 May 2016 and 22 May 2022 with no language restrictions. Thirty-nine studies were included for quantitative analysis, providing data from 77,412 participants. Adherence to both diet-quality indices and a healthy/prudent dietary pattern was inversely associated with overall mortality (RR, 0.81; 95% CI, 0.77–0.86; RR, 0.80; 95% CI, 0.70–0.92, respectively) and with cancer-specific mortality (RR, 0.86; 95% CI, 0.79–0.94; RR, 0.79; 95% CI, 0.64–0.97, respectively). These associations could be observed following assessment of dietary patterns either pre- and/or postdiagnosis. For unhealthy/western dietary patterns, high adherence was associated with overall mortality (RR, 1.26; 95% CI, 1.08–1.47). Although the certainty of evidence was rated as low, we conclude that there are no reservations against high adherence to healthy dietary patterns or indices in cancer survivors.

## 1. Introduction

With respect to causes of mortality, cancer is either number one or two in 112 countries for people under the age of 70 years. In 2020, global cancer incidence was estimated at 19.3 million cases and cancer mortality at 10 million deaths. Moreover, a 47% increase in new cancer cases is expected by 2040, resulting in 28.4 million new people affected [1]. Due to technical advancements in early detection as well as therapy procedures for cancer, the number of cancer survivors is growing rapidly, with more than 50.5 Mio. patients diagnosed within the last five years in 2020 [2,3]. The highest survival rates are reported in Australia, Canada, New Zealand, and the United States as well as in North European countries such as Finland, Iceland, Norway, and Sweden [4]. With respect to different sites of cancer, the five-year survival rate for breast cancer was 85% or more in 24 countries. For colon cancer, it was between 50–70% in 30 countries, and for lung cancer, it was 5–30% in 61 countries [2].

The systematic analyses of the Global Burden of Disease Study Group showed that in 2019, 44.4% of cancer deaths and 42% of cancer disability-adjusted life-years (DALYs) worldwide are attributable to modifiable risk factors. Dietary risks constitute the second most common risk factor causing 6.0% of deaths and 5.6% of DALYs, respectively [5]. There are no specific dietary recommendations for cancer survivors, and the World Cancer Research Fund (WCRF) analysis for breast cancer survivors found only limited evidence for the consumption of individual nutrients and foods [6]. Therefore, both the WCRF and the American Cancer Society (ACS) recommend that cancer survivors should follow the current recommendations for cancer prevention and should receive individualized dietary counseling [6,7]. To date, most of the studies investigating mortality and cancer recurrence in cancer survivors have examined the association with individual nutrients, foods, or food groups [7,8,9,10,11]. As these are consumed in combination rather than individually, dietary patterns and diet-quality indices may represent more useful tools to reflect and evaluate an individual’s diet and to enable comparisons across individuals or associations with several outcomes. By applying dietary patterns and diet-quality indices to the analysis of various diseases, the data provided can be used to derive and establish future dietary recommendations, thereby contributing to the enhancement of public health, or in this case, the health of cancer survivors [12]. The association of adherence to dietary patterns and diet-quality indices on mortality (however, not including cancer-specific mortality) and cancer recurrence in cancer survivors were synthesized in a previous systematic review and meta-analysis [13]. The literature search included only studies published until May 2016. Thus, the aim of this study was to perform an updated systematic review and meta-analysis to examine how adherence to diet-quality indices and dietary patterns affect mortality and cancer recurrence among cancer survivors. In an extension of the approach of the previous analysis, we wanted to focus on studies reporting postdiagnosis adherence and cancer-specific mortality as well.

## 2. Methods

The protocol of the previous version of this systematic review and meta-analysis has been registered in the PROSPERO International Prospective Register of Systematic Reviews (https://www.crd.york.ac.uk/prospero/display_record.php?ID=CRD42015023684 (accessed on 12 June 2023); no. CRD42015023684). The Preferred Reporting Items for Systematic Reviews and Meta-Analyses (PRISMA) statement, PRISMA for abstract [14], and PRISMA for searching [15] were used to plan, conduct, and report the current study.

### 2.1. Data Sources and Searches

The systematic literature search was conducted in two electronic databases (PubMed and Web of Science), between 18 May 2016 and 22 May 2022, with no language restrictions on 23 May 2022. The search strategies were adapted from Schwedhelm et al. [13] and are given in the Supplementary Materials Table S1.

In addition, a hand search of references from selected articles was performed to identify other eligible studies as well as systematic reviews and meta-analyses. Eligible studies selected by Schwedhelm et al. [13] were included in this systematic review and meta-analysis.

### 2.2. Study Selection

The evaluation of eligible studies was conducted by two authors (A.T. and G.H.), and any uncertainties were resolved by discussion with the third author (L.S.). After manually removing duplicates, studies were included in the systematic review and meta-analysis if they met the following criteria): (1) cohort study (prospective and retrospective); (2) diagnosed with primary cancer/survivor of any type of cancer (every neoplasm type, without cervical lesions and precancerous lesions of the colon); (3) use of a priori-based diet-quality indices (such as the Mediterranean diet (MED), Dietary Approach to Stop Hypertension (DASH), Healthy Eating Index (HEI), and WCRF/American Institute for Cancer Research (AICR) dietary guidelines adherence score (ACS)); (4) use of dietary patterns assessed by principal component analysis (healthy/prudent, unhealthy/western); (5) overall mortality and/or cancer-specific mortality and/or cancer recurrence reported as relative risk (RR) or hazard ratio (HR) with the corresponding 95% confidence interval (CI); and (6) study population restricted to adults only (≥18 years). PICOS criteria for eligible studies are given in Table 1.

For quantitative analysis, studies using the same cohort were screened for cancer type, same indices/patterns, and pre- or postdiagnosis diet assessment. In case of overlapping data, the study that provided more information was selected to avoid duplication of data. Studies with the same cohort but different diagnosed cancer types were included in the quantitative analysis. The same applied to studies with the same cohort but different indices or different times of dietary assessment (prediagnosis or postdiagnosis).

### 2.3. Data Extraction

Data extraction from the included studies was carried out as follows: (1) name of the first author and year of publication; (2) country; (3) cohort name; (4) types of outcome (overall mortality, cancer-specific mortality, non-cancer-specific mortality, and cancer recurrence); (5) sample size of the population; (6) mean follow-up duration in years; (7) sex of participants; (8) mean age of participants at diagnosis in years; (9) tumor characteristics; (10) assessment of recurrence; (11) exposure assessment and timeframe; (12) components of score and score range; (13) adjustment factors; and (14) multivariate-adjusted risk estimates (RR or HR with the corresponding 95% CI) comparing highest vs. lowest category. If a study provided more than one multivariate-adjusted model, the model with the most adjusted variables was selected. Furthermore, when combining outcomes, e.g., mortality and cancer progression, risk estimates were not extracted. All studies previously selected by Schwedhelm et al. [13] were reviewed again to extract further values of outcomes needed for this analysis.

### 2.4. Risk of Bias Assessment

An adjusted version of the Cochrane Risk of Bias in Nonrandomized Studies of Interventions (ROBINS-I) tool was used to evaluate the risk of bias in each study. [16] It includes seven domains of bias due to: (1) confounding; (2) selection of participants; (3) exposure assessment; (4) misclassification during follow-up; (5) missing data; (6) measurement of the outcome; and (7) selective reporting of the results. The possible judgments were low risk of bias, moderate risk of bias, high risk of bias, and no information. A description of each domain is provided in the Supplementary Material (Table S2).

### 2.5. Certainty of Evidence

The certainty of evidence for diet quality indices, healthy/prudent dietary patterns, unhealthy/western dietary patterns in association with overall mortality, cancer-specific mortality, and cancer recurrence was evaluated using the Grades of Recommendation, Assessment, Development, and Evaluation (GRADE) approach [17]. The GRADE approach allows for consideration of the within-study risk of bias, inconsistency, indirectness and imprecision between the studies, publication bias, magnitude of the effect, and dose-response relationship. The GRADE approach classifies the certainty of evidence into one of four levels: high, moderate, low, and very low. Two reviewers (A.T. and L.S.) independently rated the certainty of evidence, with disagreements being resolved by consensus. We used GRADEpro to elaborate evidence profiles (GRADEpro GDT: GRADEpro Guideline Development Tool [Software], McMaster University and Evidence Prime, 2021. Available at gradepro.org (accessed on 12 June 2023)).

### 2.6. Statistical Analysis

The meta-analysis was conducted by pooling the most multivariable-adjusted risk estimates (RR or HR) of the highest compared with the lowest category of dietary adherence using a random-effects model with the Der Simonian-Laird method [18]. The uncertainty of pooled effect estimates was minimized by using an inverse variance method. Each study was weighted by the inverse of the variance of the effect estimate. For each analysis, the studies were grouped by cancer type. First, prediagnosis and postdiagnosis dietary assessments were combined, then postdiagnosis dietary assessments were analyzed separately. Additional subgroup analyses were performed for the type of index. Furthermore, postdiagnosis diet assessments for specific cancer types were analyzed with the type of index as subgroups if at least three results were available. The results of the syntheses are shown as forest plots.

Heterogeneity was estimated using the Cochrane Q test and the inconsistency test (*I*^2^). As recommended by Higgins et al., an *I*^2^ of 25% was considered as low heterogeneity, an *I*^2^ of 50% as moderate heterogeneity, and an *I*^2^ of 75% as high heterogeneity [19]. Publication bias and small study effects were tested by using funnel plots when at least ten studies were included in the meta-analysis. All analyses were conducted using Review Manager (RevMan) version 5.4.1 (Copyright © 2023 The Cochrane Collaboration).

## 3. Results

### 3.1. Literature Search and Study Characteristics

**Figure 1:**
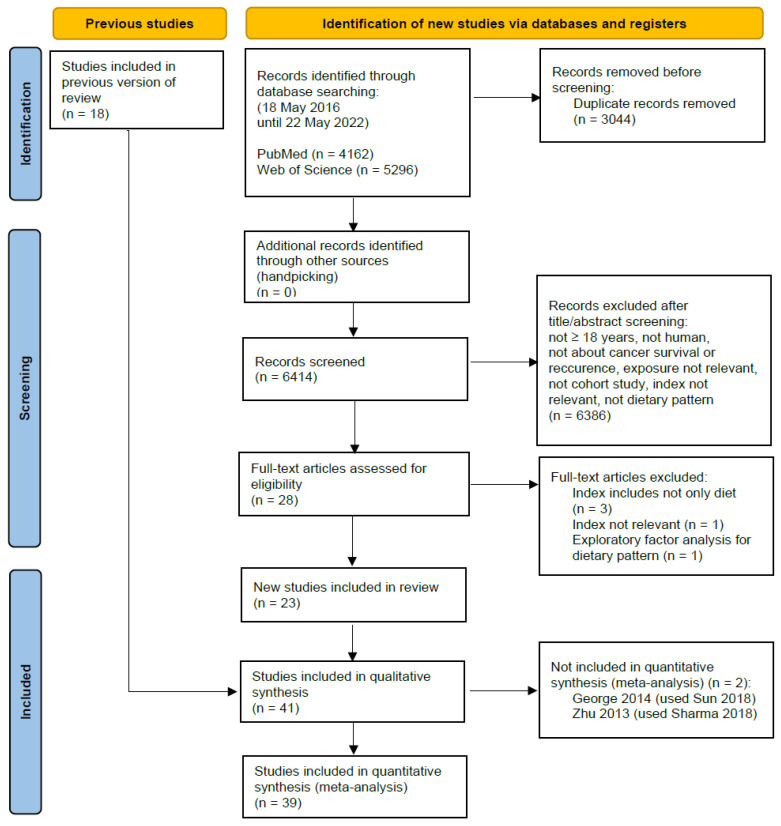
Flow chart of the literature search and selection process.

The flow chart depicting the steps of the systematic search and selection process is given in Figure 1.

Overall, 6414 articles were identified through the database search and manual searches without duplicates, leaving 28 cohort studies for full-text analysis after title/abstract screening. In the process of full-text analysis, five articles were excluded [20,21,22,23,24], resulting in 23 new studies. Together with the 18 included studies from the previous review by Schwedhelm et al. [13], a total of 41 studies were included in the current qualitative synthesis. Excluded full-text articles with the reason for exclusion are summarized in Supplementary Material Table S3, while Table S4 provides an overview of studies using the same cohort, the screened variables, and the final decision of whether the study was included or excluded [25,26].

The detailed characteristics of the included studies are given in Supplementary Material Table S5. Overall, 39 studies were included for quantitative analysis providing 77,412 participants. During follow-ups, 23,046 mortality cases occurred, with 11,208 of them being cancer-specific. Additionally, there were 2531 cases of cancer recurrence. The cohort size varied from 230 to 6370 cancer survivors, with a median follow-up time of 2.2 to 17.2 years. Breast cancer was the most commonly investigated cancer, with 14 studies, followed by 11 studies examining colorectal cancer. Three studies focused on prostate cancer, three on ovarian cancer, and one study each on head and neck squamous cell carcinoma, gastric cancer, non-Hodgkin lymphoma, multiple myeloma, hepatocellular carcinoma, and gynecological cancer. A total of three studies did not differ by cancer type.

Most studies used a validated food frequency questionnaire to assess dietary intake. Two studies used the 24-h recall method instead, and one study evaluated diet with a 7-day dietary record for one of the two included cohorts. A total of 22 studies used a prediagnosis diet assessment, 24 studies used a postdiagnosis diet assessment, and seven provided both data. With respect to diet quality indices, HEI (1995, 2005, 2010, or 2015) was used in eleven studies, alternate Healthy Eating Index (aHEI) in seven studies, MED or an adapted version like the alternate Mediterranean Diet (aMED) in fourteen studies, DASH in nine studies, ACS in four studies, and WCRF in three studies. The Recommended Food Score and healthful Plant-based Diet were each used in two studies, and the Chinese Healthy Eating Index, Chinese Food Pagoda (2007 and 2016), Australian Dietary Guideline Index, Dutch Healthy Diet Index, and Diet Quality Index-Revised were each used in one study. Fifteen of the included studies investigated diet quality using dietary patterns obtained by principal component analysis. Both a healthy/prudent dietary pattern and an unhealthy/western dietary pattern were derived from 14 studies, with 13 studies examining both.

Overall mortality was reported in 36 studies. Two studies [27,28] did not report this outcome and were calculated by combining the HRs for cancer-specific mortality and non-cancer-specific mortality. A total of 28 studies evaluated cancer-specific mortality, while eight studies assessed cancer recurrence.

Twenty-six of the included studies were conducted in the United States. Seven studies were conducted in European countries: three from Germany, two from Italy, one from Portugal, and one from the Netherlands. One cohort study was conducted in Australia, one was conducted in Canada, and three studies were conducted in China.

Supplementary Material Tables S6–S8 summarize the included studies with respect to diet-quality indices and/or dietary patterns investigated.

### 3.2. Risk of Bias Assessment

The potential risk of bias was assessed using an adjusted version of the ROBINS-I tool. Supplementary Material Table S9 provides comprehensive information on each domain for each study included in the present systematic review. As summarized in Table 2, the overall judgment for bias was high risk for 18 studies, although 16 of them were judged for high risk only in one domain (bias due to confounding). Another 18 studies were assessed with moderate potential risk of bias. Three studies, George et al. (2011), Meyerhardt et al., and Van Blarigan et al. [29,30,31], did not provide information in one or more domains and received the overall judgment of “no information”.

### 3.3. Main Outcomes

Synthesized risk ratios of all analyses with at least three studies included are shown in Table 3 (overall mortality), Table 4 (cancer-specific mortality), and Table 5 (cancer recurrence). The corresponding Forest plots are represented in Supplementary Material Figures S1–S18, as indexed in Table 3, Table 4 and Table 5.

The certainty of evidence for the major outcomes is summarized in Table 6. In brief, adherence to a high-quality diet was inversely associated with overall mortality (RR, 0.81; 95% CI, 0.77–0.86, low certainty of evidence for diet-quality indices; RR, 0.80; 95% CI, 0.70–0.92, low certainty of evidence for healthy/prudent dietary patterns, respectively), and with cancer-specific mortality (RR, 0.86; 95% CI, 0.79–0.94, low certainty of evidence for diet-quality indices; RR, 0.79; 95% CI, 0.64–0.97, low certainty of evidence for healthy/prudent dietary patterns, respectively). In contrast, no inverse association could be observed for cancer recurrence (RR, 1.10; 95% CI, 1.02–1.19, low certainty of evidence for diet-quality indices; RR, 0.89; 95% CI, 0.73–1.09, very low certainty of evidence for healthy/prudent dietary patterns, respectively). With respect to unhealthy/western dietary patterns, high adherence was significantly associated with overall mortality (RR, 1.26; 95% CI, 1.08–1.47, low certainty of evidence) but not with cancer-specific mortality (RR, 1.21; 95% CI, 0.96–1.53, very low certainty of evidence) or cancer recurrence (RR, 1.17; 95% CI, 0.75–1.84, very low certainty of evidence).

### 3.4. Subgroup Analyses–Cancer Types

With respect to breast cancer, adherence to diet-quality indices was inversely associated with overall mortality (RR, 0.83; 95% CI, 0.77–0.91), which was still the case following post-diagnostic assessments only (RR, 0.83; 95% CI, 0.75–0.92). Adherence to an unhealthy/western dietary pattern was associated with overall mortality in breast cancer survivors (RR, 1.36; 95% CI, 1.11–1.66). Regarding colorectal cancer survivors, inverse associations with overall mortality were found following adherence to high-quality diets (RR, 0.85; 95% CI, 0.78–0.93), and this observation could still be made when only postdiagnosis assessments were considered (RR, 0.78; 95% CI, 0.67–0.91) (Table 3). In addition, cancer-specific mortality yielded an inverse association with adherence to diet-quality indices (RR, 0.88; 95% CI, 0.77–0.99). In contrast, overall mortality was higher in colorectal cancer survivors adhering to an unhealthy/western dietary pattern after diagnosis (RR, 1.47; 95% CI, 1.05–2.05) (Table 4).

### 3.5. Subgroup Analyses–Type of Diet Quality Index

Inverse associations with overall mortality following assessments of dietary indices were found for HEI (RR, 0.81; 95% CI, 0.74–0.88), aHEI (RR, 0.82; 95% CI, 0.72–0.94), MED (RR, 0.77; 95% CI, 0.72–0.83), and DASH (RR, 0.86; 95% CI, 0.80–0.93). Comparable results could be obtained considering only postdiagnostic dietary analyses: HEI (RR, 0.73; 95% CI, 0.64–0.82), aHEI (RR, 0.81; 95% CI, 0.73–0.89), MED (RR, 0.75; 95% CI, 0.68–0.84), and DASH (RR, 0.81; 95% CI, 0.74–0.88) (Table 3). For cancer-specific mortality (Table 4), inverse associations were found for HEI (RR, 0.86; 95% CI, 0.76–0.98), MED (RR, 0.84; 95% CI, 0.73–0.96), and DASH (RR, 0.84; 95% CI, 0.76–0.93). Taking postdiagnostic dietary evaluations into account, only the DASH diet resulted in a significant inverse association (RR, 0.78; 95% CI, 0.68–0.90) (Table 4).

### 3.6. Small Study Effects and Publication Bias

To test for small study effects and publication bias, funnel plots were used and analyzed for symmetry/asymmetry. Funnel plots were created for thirteen analyses with ten or more studies included and are presented in Supplementary Material Figures S19–S31. The funnel plots in Figures S19–S22, as well as Figures S24–S31, indicate little to moderate asymmetry. Therefore, publication bias and/or small-study effects cannot be ruled out as an influencing factor on the results of the present meta-analysis.

## 4. Discussion

The purpose of this updated systematic review and meta-analysis was to investigate the associations of adherence (now including postdiagnosis adherence) to dietary indices and dietary patterns on mortality and cancer recurrence among cancer survivors. Furthermore, cancer-specific mortality was examined for the first time in this update. In summary, 21 new studies were added to the quantitative analysis, resulting in a total of 39 studies providing data on 77,412 patients.

Higher adherence to diet-quality indices and healthy/prudent dietary patterns showed an inverse association with overall mortality among cancer survivors, whereas unhealthy/western dietary patterns were associated with an increase. In breast and colorectal cancer survivors, diet-quality indices were also inversely associated with the risk of overall mortality, which is in line with findings of previous meta-analyses [13,66]. Similar to overall mortality, an inverse association was observed for cancer-specific mortality risk with high adherence to diet-quality indices and also for adherence to a healthy/prudent dietary pattern. However, with adherence after diagnosis only, the association was no longer present. Furthermore, high adherence to unhealthy/western dietary patterns showed no association with cancer-specific mortality, regardless of the time of dietary assessment. We could not find an association between adherence to either a healthy/prudent or an unhealthy/western dietary pattern and cancer recurrence, thus repeating the results of the previous analyses by Schwedhelm et al. [13]. This might be due to the fact that only a few studies have analyzed cancer recurrence, and often, no adjustment for the cancer stage and/or treatment was carried out.

With respect to diet-quality indices, an inverse association with overall mortality risk was observed for higher adherence to the HEI, AHEI, MED, and DASH. These data confirm the results of meta-analyses by Morze et al. [67,68]. In addition, these relations were still present when postdiagnosis adherence was investigated. A lower risk of cancer-specific mortality was found following higher adherence to HEI, DASH, and MED; however, only the DASH diet still exhibited these benefits when postdiagnosis adherence was considered exclusively.

Overall, diet-quality indices, as well as dietary patterns with beneficial associations, can differ with respect to specific characteristics, but analyses of the same cohorts with different dietary indices showed moderate to strong correlations between them [69]. Likewise, in the Dietary Patterns Methods Project, Liese et al. [70] analyzed the correlation of four indices, HEI-2010, AHEI-2010, DASH, and the alternate MED, across three cohorts. A moderate to strong correlation was found between all of them, with the lowest correlation range of 0.48–0.54 between HEI-2010 and the alternate MED and the highest correlation range of 0.69–0.72 between HEI-2010 and DASH. A typical profile of a favorable diet includes a plant-based approach with a high intake of fruits, vegetables, and whole grains supplemented by fish, dairy products, and white meat such as poultry while simultaneously avoiding red meat, processed meat, and high-caloric beverages. On the other hand, detrimental patterns such as the Western diet exhibit opposing characteristics.

Fruits and vegetables are food groups with a high density of nutrients such as dietary fiber, selenium, and vitamins A, C, and E, as well as with high amounts of phytochemicals, such as carotenoids, flavonoids, phenols, isothiocyanates, dithiolthiones, glucosinolates and indoles, allium compounds, plant sterols, limonene, and protease inhibitors. All of these agents are potential anticarcinogens due to their ability to have antioxidative effects, induce detoxification enzymes, bind carcinogens, inhibit nitrosamine formation, alter hormone metabolism, suppress proliferation, and others [71]. Foods high in vitamin C may reduce the risk of lung cancer in tobacco smokers and colorectal cancer. The antioxidant effects of carotenoids, for example, include quenching of free radicals and neutralization of reactive oxygen species (ROS), and vitamin C has protective effects against nitrates and lipid peroxidation. However, data supporting a correlation between circulating levels of vitamin C and the risk of cancer are limited [72]. Strong evidence was found for a probable protective relationship between colorectal cancer risk and foods containing dietary fiber and whole grains [73]. Whole grains are rich in fiber, oligosaccharides, and fermentable carbohydrates, which are known to be protective against cancer [74]. Gut bacteria are known to metabolize dietary fiber, which results in the production of short-chain fatty acids such as butyrate, propionate, or acetate, which have been shown to regulate tumor formation and growth. Short-chain fatty acids are active in the regulation of oxidative stress, and butyrate, in particular, is involved in the process of colon cancer cell apoptosis and the inhibition of tumor angiogenesis [75]. Whole-grain foods are also a good source of antioxidants and phytoestrogens, the latter involved in hormone metabolism, which also has an effect on cancer regulation [74]. With dietary fiber, the transit time in the intestine is reduced, thereby shortening the amount of time epithelial cells are exposed to carcinogens [76]. In addition, whole grains are digested more slowly. As a result, blood glucose levels rise more slowly, and insulin levels are not as high. Elevated blood glucose and insulin levels may increase the risk of colon cancer [74]. Fish and seafood represent a potent source of omega-3 fatty acids. Among these, eicosapentaenoic acid (EPA) and docosahexaenoic acid form eicosanoids that provide anti-inflammatory characteristics. Moreover, EPA and eicosanoids derived by EPA can inhibit the synthesis of eicosanoids derived from arachidonic acid (AA), which have been shown to suppress cell adhesion and have an impact on some hallmarks of cancer, such as inducing or accessing angiogenesis, resisting cell death, and sustaining proliferative signaling [77]. Another food group that may influence cancer development is dairy products, mostly due to the high amount of calcium. Milk and dairy products are associated with increased blood levels of insulin-like growth factor 1, which may promote the development of prostate cancer [78]. In contrast, there is strong evidence for calcium reducing the risk of colorectal cancer, for example, via neutralizing secondary bile acids that can damage epithelial cells [79,80]. In addition to calcium, a number of different compounds in dairy products, such as lactic acid-producing bacteria, lactoferrin, or butyrate, may exert protective effects [81].

Carcinogenic N-nitroso compounds are an exogenous component of processed meat but can be formed endogenously as well due to the high heme-iron content of red meat. Moreover, the heating of meat at high temperatures yields carcinogenic heterocyclic amines and polycyclic aromatic hydrocarbons. Taken together, there is strong evidence for a correlation between high consumption of red and processed meat and increased risk for colorectal cancer [82]. The same can be said for drinking alcoholic beverages with respect to the risk of developing cancers of the breast, colorectum, larynx, mouth, liver, pharynx, and esophagus [83]. The ethanol metabolite acetaldehyde is a known carcinogen as it can inhibit DNA synthesis and repair and can cause DNA mutation. In addition, DNA methylation can be altered by acetaldehyde through its effect on DNA methyltransferase and on the synthesis of S-adenosyl-L-methionine [84]. Ethanol itself has an impact on the development of oxidative stress and inflammation. During the metabolization of ethanol to acetaldehyde by CYP2E1, large amounts of ROS are released. This is supported by the production of pro-inflammatory cytokines, leading to lipid peroxidation, changes in cell cycle behavior, promotion of cell proliferation and metastasis, and angiogenesis [85,86]. Another effect of high ethanol consumption may be dysbiosis of the microbiome with a disruption of the intestinal barrier. This enables bacteria to enter the blood, which promotes inflammatory responses and, thereby, carcinogenesis [87]. In this systematic review, we found positive effects of MED, which usually includes moderate amounts of red wine. However, different MED scores used different thresholds for “moderate” consumption [88,89]. The potential benefits of polyphenols found in red wine may, in part, explain our findings [90]. It should be noted that dietary patterns not only include the respective food groups but may include “eating rituals” as well. Red wine is not a constitutional part of a meal in countries outside the Mediterranean area [91]. Since the mechanisms by which alcohol affects cancer risk are not yet fully elucidated, the promotion of red wine as a habitual part of a diet does not seem appropriate.

### Strengths and Limitations

The interpretation of the results of this systematic review should be conducted considering its limitations. Different attempts were made to keep these limitations as small as possible using various state-of-the-art tools. In cohort studies, the interpretation of causality is limited, and bias is more likely when compared to randomized controlled trials. Therefore, the ROBINS-I tool was used to assess the risk of bias in each included study, resulting in 18 studies with a moderate risk of bias, 18 studies with a high risk of bias, and 3 studies with insufficient information to assess the risk of bias correctly. It should be noted that randomized controlled trials are difficult to conduct over a long follow-up period. In addition, the timing of dietary assessment and follow-up varied among the included studies. To reduce the risk of bias due to confounding, the most adjusted HRs were chosen, and almost all included studies reported HR adjusted for sex, age, and smoking, but the majority of studies did not adjust for cancer stage and treatment. However, several studies excluded people who died within 1 year of cancer diagnosis or performed a sensitivity analysis regarding this outcome, with no changes in findings. The majority of the included studies assessed the diet at only one time point. Therefore, if a change in diet occurred after the dietary assessment, it is not included in the results. Nevertheless, changes in overall diet sometimes occur after a cancer diagnosis, but the current evidence suggests that these changes are small [59,92,93,94]. Unpublished data were not included, which could cause an overestimation of the true effect. The results of this study are based on data from studies conducted in the United States, followed by European countries, with only a few representing populations from Canada, Australia, and Asia. No studies conducted in South America or Africa were included. Therefore, the results may not be generalizable to other populations that may have different dietary patterns, risk factors, medical care, and cancer screening.

Apart from its limitations, this systematic review has several strengths as well. Two databases were searched without language restriction yielding 41 studies in the systematic review and 39 studies in the meta-analysis. Accordingly, data from an overall high number of participants could be synthesized. This study focused on cancer survivors, who represent an increasing number of patients with a special interest in the topic of this study, which is shared by organizations and authorities working in this field of research. A prominent example is the Global Cancer Update Programme of the WCRF continuously reviewing the long-term effects of lifestyle factors (diet, physical activity, and weight management) following diagnosis of colorectal and prostate cancer, with an already published Update Report for breast cancer survivors [95]. The present systematic review set a focus on postdiagnosis dietary assessment, with data providing new insights into this area of research. Moreover, the analysis of cancer recurrence provided a comparatively larger number of studies. With regard to the correct procedure, the PRISMA guidelines were followed in the conduct of this systematic review and meta-analysis. Lastly, an adjusted version of the ROBINS-I assessment tool was used, which is regarded to be the recommended instrument for assessing the risk of bias in nonrandomized studies.

## 5. Conclusions

In the present study, a high-quality diet, as defined by adherence to various dietary quality indices, was inversely associated with the risk of overall mortality and cancer-specific mortality among cancer survivors, even when dietary adherence starts after diagnosis. Considering specific cancer sites, this relationship was also found in breast and colorectal cancer survivors with respect to overall mortality. The individual diet-quality indices HEI, aHEI, MED, and DASH demonstrated beneficial associations on overall mortality, while detrimental associations on this outcome were found following an unhealthy/western dietary pattern before and/or after diagnosis. Regarding cancer-specific mortality other than breast and colorectal cancer or recurrence among cancer survivors in general, the available data are not robust enough to draw any conclusions. Since no detrimental associations could be observed, there are no reservations against adherence to healthy dietary patterns or indices in cancer survivors.

## Figures and Tables

**Table 1:** PICOS for study eligibility criteria.

	Inclusion	Exclusion
Populations/participants	Survivors of any type of cancer (every neoplasm type) Adults only (aged ≥ 18 years)	Survivors of cervical lesions Survivors of precancerous lesions of the colon Children and adolescents (aged < 18 years) Animals
Interventions/exposure	A priori-based diet-quality indices (Mediterranean diet, DASH, Healthy Eating Index, and WCRF/AICR dietary guidelines adherence score) Adherence to data-driven dietary patterns (healthy/prudent, unhealthy/Western)	Indices including not only diet (e.g., WCRF/AICR without analyzing diet score separately) Intake of supplements Single nutrients Intake of individual foods (e.g., fruit, vegetables, dairy, meat, fish, cereals, and bread) Intake of beverages (e.g., alcohol, coffee, and tea)
Comparators/Comparison	Highest versus lowest categories of exposure	
Outcomes	Overall mortality and/or cancer-specific mortality and/or cancer recurrence	Studies that did not report any of the outcomes
Study designs	Cohort studies (prospective and retrospective)	Intervention studies Case-control studies Systematic reviews

Abbreviations: DASH Dietary Approaches to Stop Hypertension, WCRF/AICR World Cancer Research Fund/American Institute for Cancer Research.

**Table 2:** Summary of the results from the risk of bias analysis. Conducted with an adapted version of the Cochrane Risk of Bias in Nonrandomized Studies of Interventions tool.

Study	Bias Due to Confounding	Bias Due to Selection of Participants	Bias Due to Exposure Assessment	Bias Due to Misclassification during Follow-Up	Bias Due to Missing Data	Bias Due to Measurement of the Outcome	Bias Due to Selective Reporting of the Results	Overall Judgment
Al Ramadhani et al. (2021) [32]	High	Low	Moderate	Moderate	Low	Low	Low	H
Anyene et al. (2021) [33]	High	Moderate	Moderate	Low	Low	Low	Low	H
Arthur et al. (2013) [34]	Moderate	Moderate	Moderate	Moderate	Low	Low	Low	M
Deshmukh et al. (2018) [35]	High	Low	High	Moderate	Low	Low	Low	H
Di Maso et al. (2020) [36]	High	Moderate	Moderate	Moderate	Low	Low	Low	H
Di Maso et al. (2021) [28]	High	Moderate	Moderate	Moderate	Low	Low	Low	H
Ergas et al. (2021) [37]	Moderate	Moderate	Moderate	Moderate	Low	Low	Low	M
Ferronha et al. (2012) [38]	Moderate	Moderate	Moderate	Moderate	Low	Low	Low	M
Fung et al. (2014) [39]	Moderate	Low	Moderate	Moderate	Low	Low	Low	M
George et al. (2011) [29]	N.I.	Low	Moderate	Moderate	Low	Low	Low	N.I.
Guinter et al. (2018) [40]	Moderate	Low	Moderate	Low	Low	Low	Low	M
Inoue-Choi et al. (2013) [41]	High	Low	Moderate	Moderate	Low	Low	Low	H
Izano et al. (2013) [27]	Moderate	Low	Moderate	Low	Low	Moderate	Low	M
Jacobs et al. (2016) [42]	Moderate	Moderate	Moderate	Low	Low	Low	Low	M
Karavasiloglou et al. (2019) [43]	High	Low	High	Moderate	Low	Low	Low	H
Kenfield et al. (2014) [44]	Moderate	Moderate	Moderate	Low	Low	Moderate	Low	M
Kim et al. (2011) [45]	Moderate	Low	Moderate	Moderate	Low	Moderate	Low	M
Kroenke et al. (2005) [46]	Moderate	Low	Moderate	Moderate	Low	Moderate	Low	M
Kwan et al. (2009) [47]	Moderate	Low	Moderate	Moderate	Low	Low	Low	M
Lee et al. (2020) [48]	High	Low	Moderate	Low	Low	Low	Low	H
Lei et al. (2021) [49]	High	Moderate	Moderate	Moderate	Low	Low	Low	H
Luo et al. (2020) [50]	Moderate	Moderate	Moderate	Moderate	Low	Low	Low	M
McCullough et al. (2016) [51]	Moderate	Low	Moderate	Moderate	Low	Low	Low	M
Meyerhardt et al. (2007) [30]	Moderate	Moderate	Moderate	Low	N.I.	N.I.	Low	N.I.
Ollberding et al. (2013) [52]	High	Moderate	Moderate	Moderate	Low	Low	Low	H
Park et al. (2022) [53]	Moderate	Low	Moderate	Low	Low	Low	Low	M
Pelser et al. (2014) [54]	Moderate	Moderate	Moderate	Moderate	Low	Low	Low	M
Ratjen et al. (2017) [55]	High	Low	Moderate	Moderate	Low	Low	Low	H
Ratjen et al. (2021) [56]	High	Low	Moderate	Moderate	Low	Low	Low	H
Sharma et al. (2018) [57]	High	Moderate	Moderate	Moderate	Low	Moderate	Low	H
Song et al. (2021) [58]	High	Low	Moderate	Low	Low	Low	Low	H
Sun et al. (2018) [59]	High	Moderate	Moderate	Low	Low	Low	Low	H
Thomson et al. (2014) [60]	High	Moderate	Moderate	Moderate	Low	Low	Low	H
Van Blarigan et al. (2020) [31]	High	Moderate	Moderate	Moderate	N.I.	N.I.	Low	N.I.
Van Zutphen et al. (2021) [61]	Moderate	Moderate	Moderate	Low	Low	Low	Low	M
Vrieling et al. (2013) [62]	Moderate	Moderate	Moderate	Moderate	Low	Moderate	Low	M
Wang et al. (2020) [63]	Moderate	Moderate	Moderate	Moderate	Low	Low	Low	M
Wen et al. (2022) [64]	High	Moderate	Moderate	Low	Low	Low	Low	H
Yang et al. (2015) [65]	High	Low	Moderate	Moderate	Low	Moderate	Low	H

Abbreviations: H serious risk of bias, M moderate risk of bias, N.I. no information.

**Table 3:** Risk of overall mortality comparing the highest versus lowest category of pre- and postdiagnosis and postdiagnosis-only adherence to diet-quality indices, healthy/prudent dietary pattern, and unhealthy/western dietary pattern with subgroups. Data are shown only if at least three studies per subgroup were included.

Exposure	No. of Studies	Risk Ratio (95% CI)	*I* ^2^	Forest Plot
All diet-quality indices, Cancer subtypes				Figure S1
Breast cancer	10	0.83 [0.77, 0.91]	58%	
Colorectal cancer	10	0.85 [0.78, 0.93]	56%	
All cancer types	3	0.74 [0.65, 0.86]	50%	
All diet-quality indices, Cancer subtypes, postdiagnosis only				Figure S2
Breast cancer	8	0.83 [0.75, 0.92]	43%	
Colorectal cancer	6	0.78 [0.67, 0.91]	45%	
All cancer types	3	0.74 [0.65, 0.86]	50%	
Diet-quality indices				Figure S3
HEI	10	0.81 [0.74, 0.88]	41%	
AHEI	6	0.82 [0.72, 0.94]	60%	
MED	13	0.77 [0.72, 0.83]	23%	
DASH	9	0.86 [0.80, 0.93]	28%	
ACS	4	0.83 [0.69, 1.00]	56%	
WCRF	3	0.89 [0.77, 1.02]	0%	
Diet-quality indices, postdiagnosis only				Figure S4
HEI	5	0.73 [0.64, 0.82]	29%	
AHEI	3	0.81 [0.73, 0.89]	0%	
MED	6	0.75 [0.68, 0.84]	28%	
DASH	5	0.81 [0.74, 0.88]	0%	
ACS	3	0.78 [0.58, 1.05]	58%	
WCRF	3	0.88 [0.75, 1.04]	0%	
Healthy/prudent dietary pattern				Figure S5
Breast cancer	4	0.84 [0.61, 1.15]	53%	
Colorectal cancer	5	0.89 [0.77, 1.04]	19%	
Healthy/prudent dietary pattern, postdiagnosis only				Figure S6
Breast cancer	3	0.84 [0.52, 1.34]	68%	
Colorectal cancer	3	0.93 [0.66, 1.30]	66%	
Unhealthy/western dietary pattern				Figure S7
Breast cancer	4	1.36 [1.11, 1.66]	4%	
Colorectal cancer	5	1.28 [0.96, 1.70]	76%	
Unhealthy/western dietary pattern, postdiagnosis only				Figure S8
Breast cancer	3	1.31 [0.91, 1.89]	37%	
Colorectal cancer	3	1.47 [1.05, 2.05]	53%	

Abbreviations: ACS American Cancer Society, AHEI Alternative Healthy Eating Index, DASH Dietary Approaches to Stop Hypertension, CI confidence interval, HEI Healthy Eating Index, *I*^2^ inconsistency, MED Mediterranean Diet Score, WCRF World Cancer Research Fund Score.

**Table 4:** Risk of cancer-specific mortality comparing the highest versus lowest category of pre- and postdiagnosis and postdiagnosis-only adherence to diet-quality indices, healthy/prudent dietary pattern, and unhealthy/western dietary pattern with subgroups. Data are shown only if at least three studies per subgroup were included.

Exposure	No. of Studies	Risk Ratio (95% CI)	*I* ^2^	Forest Plot
Cancer subtypes				Figure S9
Breast cancer	9	0.92 [0.79, 1.06]	69%	
Colorectal cancer	5	0.88 [0.77, 0.99]	44%	
All cancer types	3	0.67 [0.47, 0.96]	72%	
Cancer subtypes, postdiagnosis only				Figure S10
Breast cancer	7	0.95 [0.76, 1.19]	73%	
Colorectal cancer	3	0.74 [0.49, 1.13]	68%	
All cancer types	3	0.67 [0.47, 0.96]	72%	
Diet-quality indices				Figure S11
HEI	11	0.86 [0.76, 0.98]	53%	
AHEI	6	0.88 [0.73, 1.06]	69%	
MED	9	0.84 [0.73, 0.96]	45%	
DASH	8	0.84 [0.76, 0.93]	0%	
ACS	3	0.85 [0.56, 1.28]	66%	
Diet-quality indices, postdiagnosis only				Figure S12
HEI	6	0.79 [0.58, 1.10]	72%	
AHEI	4	0.93 [0.77, 1.13]	27%	
MED	4	0.90 [0.69, 1.17]	64%	
DASH	5	0.78 [0.68, 0.90]	9%	
Healthy/prudent dietary pattern				Figure S13
Breast cancer	4	0.99 [0.77, 1.28]	0%	
Healthy/prudent dietary pattern, postdiagnosis only				Figure S14
Breast cancer	3	1.06 [0.77, 1.46]	0%	
Unhealthy/western dietary pattern				Figure S15
Breast cancer	4	1.01 [0.79, 1.30]	0%	
Unhealthy/western dietary pattern, postdiagnosis only	6	1.15 [0.84, 1.58]	28%	Figure S16
Breast cancer	3	1.03 [0.72, 1.46]	0%	

Abbreviations: ACS American Cancer Society, AHEI Alternative Healthy Eating Index, DASH Dietary Approaches to Stop Hypertension, CI confidence interval, HEI Healthy Eating Index, *I*^2^ inconsistency, MED Mediterranean Diet Score.

**Table 5:** Risk of cancer recurrence comparing the highest versus lowest category of pre- and postdiagnosis and postdiagnosis-only adherence to diet-quality indices, healthy/prudent dietary pattern, and unhealthy/western dietary pattern with subgroups. Data are shown only if at least three studies per subgroup were included.

Exposure	No. of Studies	Risk Ratio (95% CI)	*I* ^2^	Forest Plot
Healthy/prudent dietary pattern				Figure S17
Breast cancer	3	0.87 [0.68, 1.10]	0%	
Unhealthy/western dietary pattern				Figure S18
Breast cancer	3	0.96 [0.74, 1.25]	0%	

Abbreviations: CI confidence interval, *I*^2^ inconsistency.

**Table 6:** Certainty of evidence for associations of high adherence to diet-quality indices/dietary pattern and overall mortality, cancer-specific mortality, and cancer recurrence among cancer survivors.

N of Studies	Study Design	Risk of Bias	Inconsistency	Indirectness	Imprecision	Other Considerations	Relative Effect (95% CI)	Certainty
Diet-quality indices-Overall mortality								
28	observational studies	very serious ^a^	not serious	not serious	not serious	none	RR 0.81 (0.77 to 0.86)	⨁⨁◯◯ Low
Healthy/prudent dietary pattern-Overall mortality								
14	observational studies	very serious ^a^	not serious	not serious	not serious	none	RR 0.80 (0.70 to 0.92)	⨁⨁◯◯ Low
Unhealthy/western dietary pattern-Overall mortality								
14	observational studies	very serious ^a^	not serious	not serious	not serious	none	RR 1.26 (1.08 to 1.47)	⨁⨁◯◯ Low
Diet-quality indices-Cancer specific mortality								
23	observational studies	very serious ^a^	not serious	not serious	not serious	none	RR 0.86 (0.79 to 0.94)	⨁⨁◯◯ Low
Healthy/prudent dietary pattern-Cancer specific mortality								
8	observational studies	very serious ^a^	not serious	not serious	not serious	none	RR 0.79 (0.64 to 0.97)	⨁⨁◯◯ Low
Unhealthy/western dietary pattern-Cancer specific mortality								
8	observational studies	very serious ^a^	not serious	not serious	serious ^b^	none	RR 1.21 (0.96 to 1.53)	⨁◯◯◯ Very low
Diet-quality indices-Cancer recurrence								
3	observational studies	very serious ^a^	not serious	not serious	not serious	none	RR 1.10 (1.02 to 1.19)	⨁⨁◯◯ Low
Healthy/prudent dietary pattern-Cancer recurrence								
5	observational studies	very serious ^a^	not serious	not serious	serious ^c^	none	RR 0.89 (0.73 to 1.09)	⨁◯◯◯ Very low
Unhealthy/western dietary pattern-Cancer recurrence								
5	observational studies	very serious ^a^	not serious	not serious	serious ^d^	none	RR 1.17 (0.75 to 1.84)	⨁◯◯◯ Very low

Abbreviations: CI confidence interval; RR risk ratio. ^a.^ Downgraded by two levels for risk of bias since several studies were rated with an overall high risk of bias (predominately in the confounding domain). ^b.^ Downgraded by one level for imprecision since 95% CI overlaps null effect and is wide (95% CI: 0.96 to 1.53). ^c.^ Downgraded by one level for imprecision since 95% CI overlaps null effect and is wide (95% CI: 0.73 to 1.09). ^d.^ Downgraded by one level for imprecision since 95% CI overlaps null effect and is wide (95% CI: 0.75 to 1.84). Level of certainty: ⨁◯◯◯: Very low; ⨁⨁◯◯: Low; ⨁⨁⨁◯: Moderate; ⨁⨁⨁⨁: High.

## Data Availability

Not applicable.

## Supplementary Materials

The following supporting information can be downloaded at: https://www.mdpi.com/article/10.3390/nu15143151/s1, Table S1: Literature search strategy. Table S2: Description and decision criteria for each domain in Cochrane Risk of Bias in Nonrandomized Studies of Interventions. Table S3: Full-text articles excluded. Table S4: Comparison of included studies in the qualitative analysis with the same cohort. Table S5: Characteristics of the cohort studies included in the present meta-analysis. Table S6: Overview of included studies in the meta-analysis analyzing diet-quality indices. Table S7: Overview of included studies in the meta-analysis analyzing healthy/prudent dietary patterns. Table S8: Overview of included studies in the meta-analysis analyzing unhealthy/western dietary patterns. Table S9: Detailed risk of bias judgment for each included study in the meta-analysis. Figure S1: Forest plot showing pooled risk ratios with 95% CIs for overall mortality comparing the highest versus lowest category of adherence to diet-quality indices of 28 observational studies by subgroup: type of cancer. Figure S2: Forest plot showing pooled risk ratios with 95% CIs for overall mortality comparing the highest versus lowest category of postdiagnosis adherence to diet-quality indices of 18 observational studies by subgroup: type of cancer. Figure S3: Forest plot showing pooled risk ratios with 95% CIs for overall mortality comparing the highest versus lowest category of adherence to diet-quality indices of 28 observational studies by subgroup: type of index. Figure S4: Forest plot showing pooled risk ratios with 95% CIs for overall mortality comparing the highest versus lowest category of postdiagnosis adherence to diet-quality indices of 18 observational studies by subgroup: type of index. Figure S5: Forest plot showing pooled risk ratios with 95% CIs for overall mortality comparing the highest versus lowest category of adherence to a healthy/prudent dietary pattern of 14 observational studies by subgroup: type of cancer. Figure S6: Forest plot showing pooled risk ratios with 95% CIs for overall mortality comparing the highest versus lowest category of postdiagnosis adherence to a healthy/prudent dietary pattern of 7 observational studies by subgroup: type of cancer. Figure S7: Forest plot showing pooled risk ratios with 95% CIs for overall mortality comparing the highest versus lowest category of adherence to an unhealthy/western dietary pattern of 14 observational studies by subgroup: type of cancer. Figure S8: Forest plot showing pooled risk ratios with 95% CIs for overall mortality comparing the highest versus lowest category of postdiagnosis adherence to an unhealthy/western dietary pattern of 7 observational studies by subgroup: type of cancer. Figure S9: Forest plot showing pooled risk ratios with 95% CIs for cancer-specific mortality comparing the highest versus lowest category of adherence to diet-quality indices of 23 observational studies by subgroup: type of cancer. Figure S10: Forest plot showing pooled risk ratios with 95% CIs for cancer-specific mortality comparing the highest versus lowest category of postdiagnosis adherence to diet-quality indices of 15 observational studies by subgroup: type of cancer. Figure S11: Forest plot showing pooled risk ratios with 95% CIs for cancer-specific mortality comparing the highest versus lowest category of adherence to diet-quality indices of 23 observational studies by subgroup: type of index. Figure S12: Forest plot showing pooled risk ratios with 95% CIs for cancer-specific mortality comparing the highest versus lowest category of postdiagnosis adherence to diet-quality indices of 15 observational studies by subgroup: type of index. Figure S13: Forest plot showing pooled risk ratios with 95% CIs for cancer-specific mortality comparing the highest versus lowest category of adherence to a healthy/prudent dietary pattern of 8 observational studies by subgroup: type of cancer. Figure S14: Forest plot showing pooled risk ratios with 95% CIs for cancer-specific mortality comparing the highest versus lowest category of postdiagnosis adherence to a healthy/prudent dietary pattern of 6 observational studies by subgroup: type of cancer. Figure S15: Forest plot showing pooled risk ratios with 95% CIs for cancer-specific mortality comparing the highest versus lowest category of adherence to an unhealthy/western dietary pattern of 8 observational studies by subgroup: type of cancer. Figure S16: Forest plot showing pooled risk ratios with 95% CIs for cancer-specific mortality comparing the highest versus lowest category of postdiagnosis adherence to an unhealthy/western dietary pattern of 6 observational studies by subgroup: type of cancer. Figure S17: Forest plot showing pooled risk ratios with 95% CIs for cancer recurrence comparing the highest versus lowest category of adherence to a healthy/prudent dietary pattern of 5 observational studies by subgroup: type of cancer. Figure S18: Forest plot showing pooled risk ratios with 95% CIs for cancer recurrence comparing the highest versus lowest category of adherence to an unhealthy/western dietary pattern of 5 observational studies by subgroup: type of cancer. Figure S19: Funnel plot showing study precision against the relative risk with 95% CI for diet-quality indices of pre- and postdiagnosis diet (subgroup-analysis with cancer type) and overall mortality. Figure S20: Funnel plot showing study precision against the relative risk with 95% CI for diet-quality indices of postdiagnosis diet (subgroup-analysis with cancer type) and overall mortality. Figure S21: Funnel plot showing study precision against the relative risk with 95% CI for diet-quality indices of pre- and postdiagnosis diet (subgroup-analysis with each diet quality index) and overall mortality. Figure S22: Funnel plot showing study precision against the relative risk with 95% CI for diet-quality indices of postdiagnosis diet (subgroup-analysis with each diet quality index) and overall mortality. Figure S23: Funnel plot showing study precision against the relative risk with 95% CI for a healthy/prudent dietary pattern of pre- and postdiagnosis diet (subgroup-analysis with cancer type) and overall mortality. Figure S24: Funnel plot showing study precision against the relative risk with 95% CI for an unhealthy/western dietary pattern of pre- and postdiagnosis diet (subgroup-analysis with cancer type) and overall mortality. Figure S25: Funnel plot showing study precision against the relative risk with 95% CI for diet-quality indices of pre- and postdiagnosis diet (subgroup-analysis with cancer type) and cancer-specific mortality. Figure S26: Funnel plot showing study precision against the relative risk with 95% CI for diet-quality indices of postdiagnosis diet (subgroup-analysis with cancer type) and cancer-specific mortality. Figure S27: Funnel plot showing study precision against the relative risk with 95% CI for diet-quality indices of pre- and postdiagnosis diet (subgroup-analysis with each diet quality index) and cancer-specific mortality. Figure S28: Funnel plot showing study precision against the relative risk with 95% CI for diet-quality indices of postdiagnosis diet (subgroup-analysis with each diet quality index) and cancer-specific mortality. Figure S29: Funnel plot showing study precision against the relative risk with 95% CI for diet-quality indices of postdiagnosis diet (subgroup-analysis with each diet quality index) and overall mortality among breast cancer survivors. Figure S30: Funnel plot showing study precision against the relative risk with 95% CI for diet-quality indices of postdiagnosis diet (subgroup-analysis with each diet quality index) and cancer-specific mortality among breast cancer survivors. Figure S31: Funnel plot showing study precision against the relative risk with 95% CI for diet-quality indices of postdiagnosis diet (subgroup-analysis with each diet quality index) and overall mortality among colorectal cancer survivors.
