# Supplementary material for: Association between Dietary Indices and Dietary Patterns and Mortality and Cancer Recurrence among Cancer Survivors: An Updated Systematic Review and Meta-Analysis of Cohort Studies
Source: Nutrients. 2023 Jul 14;15(14):3151. doi: 10.3390/nu15143151 (PMC10385219; doi:10.3390/nu15143151)
Supplement: Supplementary file 1 [file nutrients-15-03151-s001.zip › Trauchburg et al Manuscript_Supplementary_Material_Figures.docx]

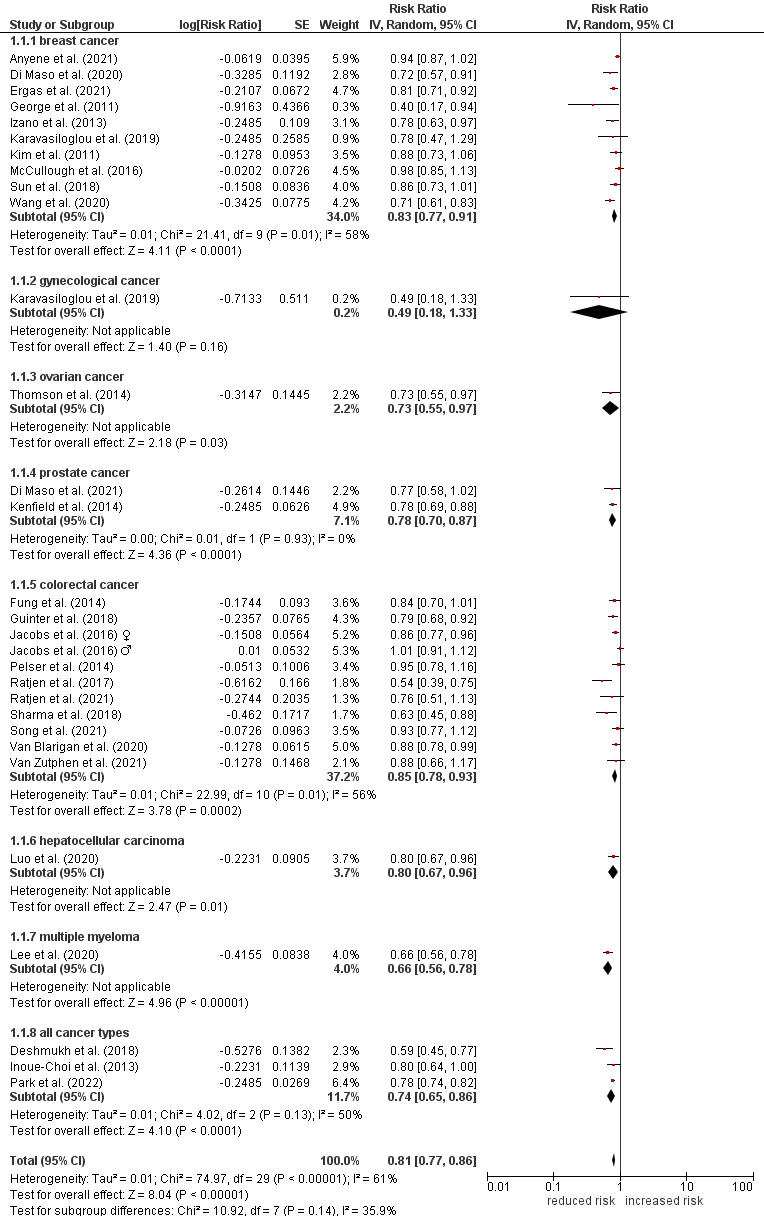


Figure S1: Forest plot showing pooled risk ratios with 95% CIs for overall mortality comparing the highest versus lowest category of adherence to diet-quality indices of 28 observational studies by subgroup: type of cancer.

The red squares represent the point of estimates of the intervention effect, the size of the red squares represents the weight assigned to each study within the meta-analysis. The horizontal lines represent the corresponding 95% CI. The black diamonds show the effect estimates for each subgroup and the black diamond at the bottom of the graph represents the overall effect estimate of all included studies.

Abbreviations: *CI* confidence interval, *df* degrees of freedom, *I²* inconsistency, *IV* inverse variance, *SE* standard error, *tau* estimate between study variance.


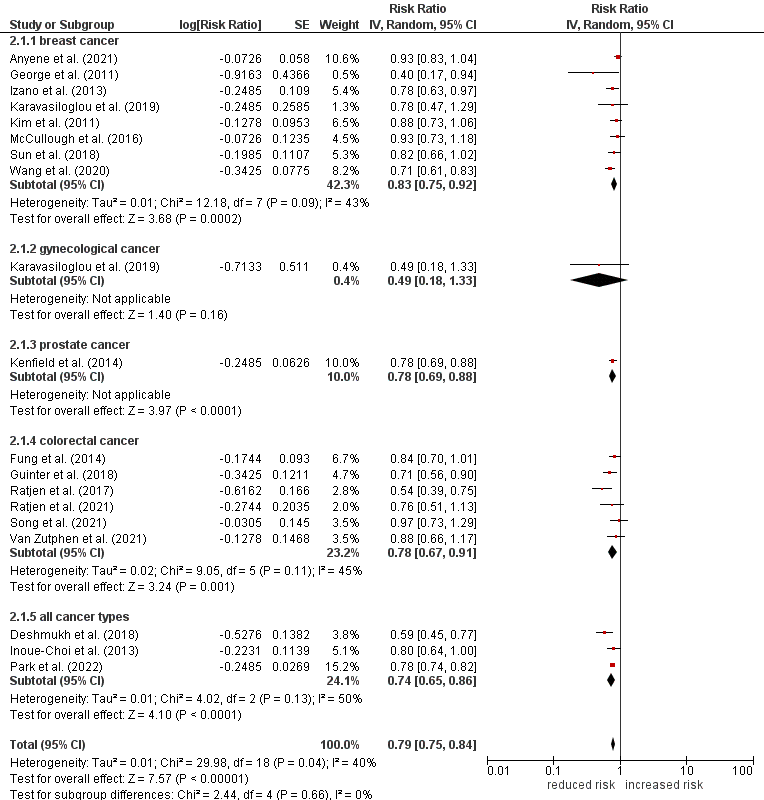


Figure S2: Forest plot showing pooled risk ratios with 95% CIs for overall mortality comparing the highest versus lowest category of postdiagnosis adherence to diet-quality indices of 18 observational studies by subgroup: type of cancer.

The red squares represent the point of estimates of the intervention effect, the size of the red squares represents the weight assigned to each study within the meta-analysis. The horizontal lines represent the corresponding 95% CI. The black diamonds show the effect estimates for each subgroup and the black diamond at the bottom of the graph represents the overall effect estimate of all included studies.

Abbreviations: *CI* confidence interval, *df* degrees of freedom, *I²* inconsistency, *IV* inverse variance, *SE* standard error, *tau* estimate between study variance.


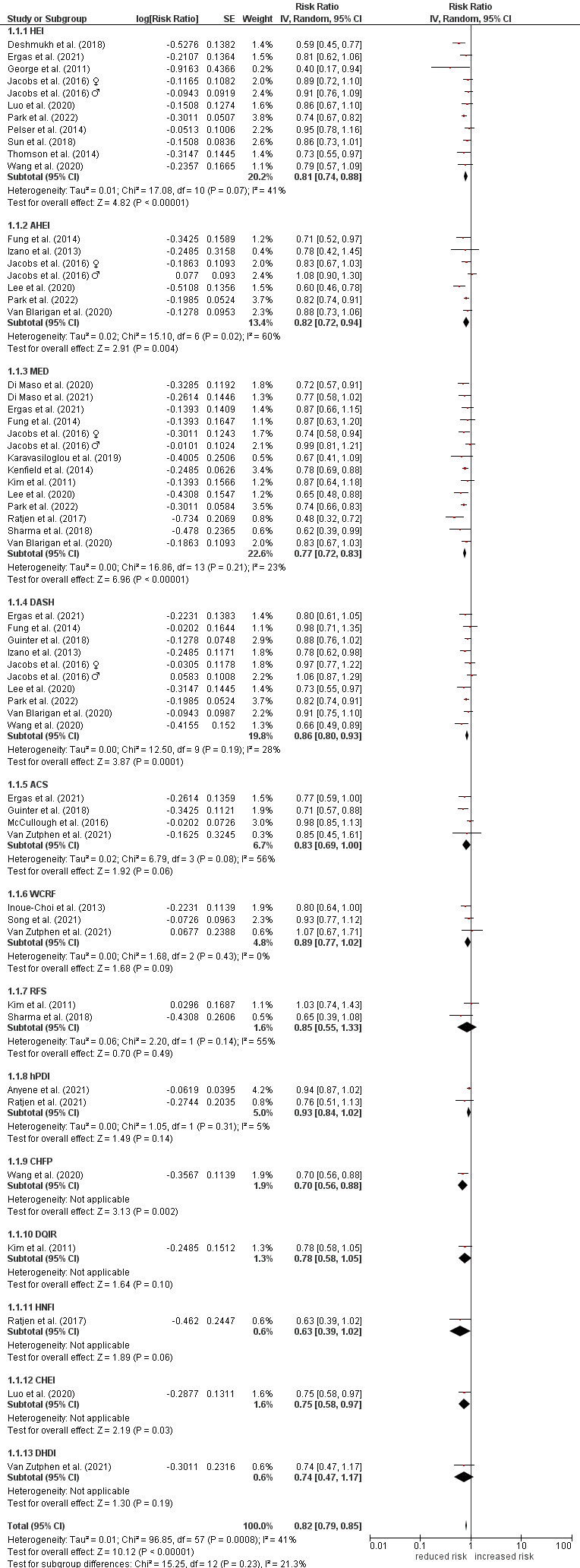


Figure S3: Forest plot showing pooled risk ratios with 95% CIs for overall mortality comparing the highest versus lowest category of adherence to diet-quality indices of 28 observational studies by subgroup: type of index.

The red squares represent the point of estimates of the intervention effect, the size of the red squares represents the weight assigned to each study within the meta-analysis. The horizontal lines represent the corresponding 95% CI. The black diamonds show the effect estimates for each subgroup and the black diamond at the bottom of the graph represents the overall effect estimate of all included studies.

Abbreviations: *ACS* American Cancer Society, *AHEI* Alternative Healthy Eating Index, *CHFP* Chinese Food Pagoda, *CI* confidence interval, *DASH* Dietary Approaches to Stop Hypertension, *df* degrees of freedom, *DHDI* Dutch Healthy Diet Index, *DQIR* Diet Quality Index-Revised, *HEI* Healthy Eating Index, *HNFI* healthy Nordic Food Index, *hPDI* healthful plant-based diet index, *I²* inconsistency, *IV* inverse variance, *MED* Mediterranean Diet Score, *SE* standard error, *tau* estimate between study variance, *RFS* Recommended Food Score, *WCRF/AICR* World Cancer Research Fund/ American Institute for Cancer Research.


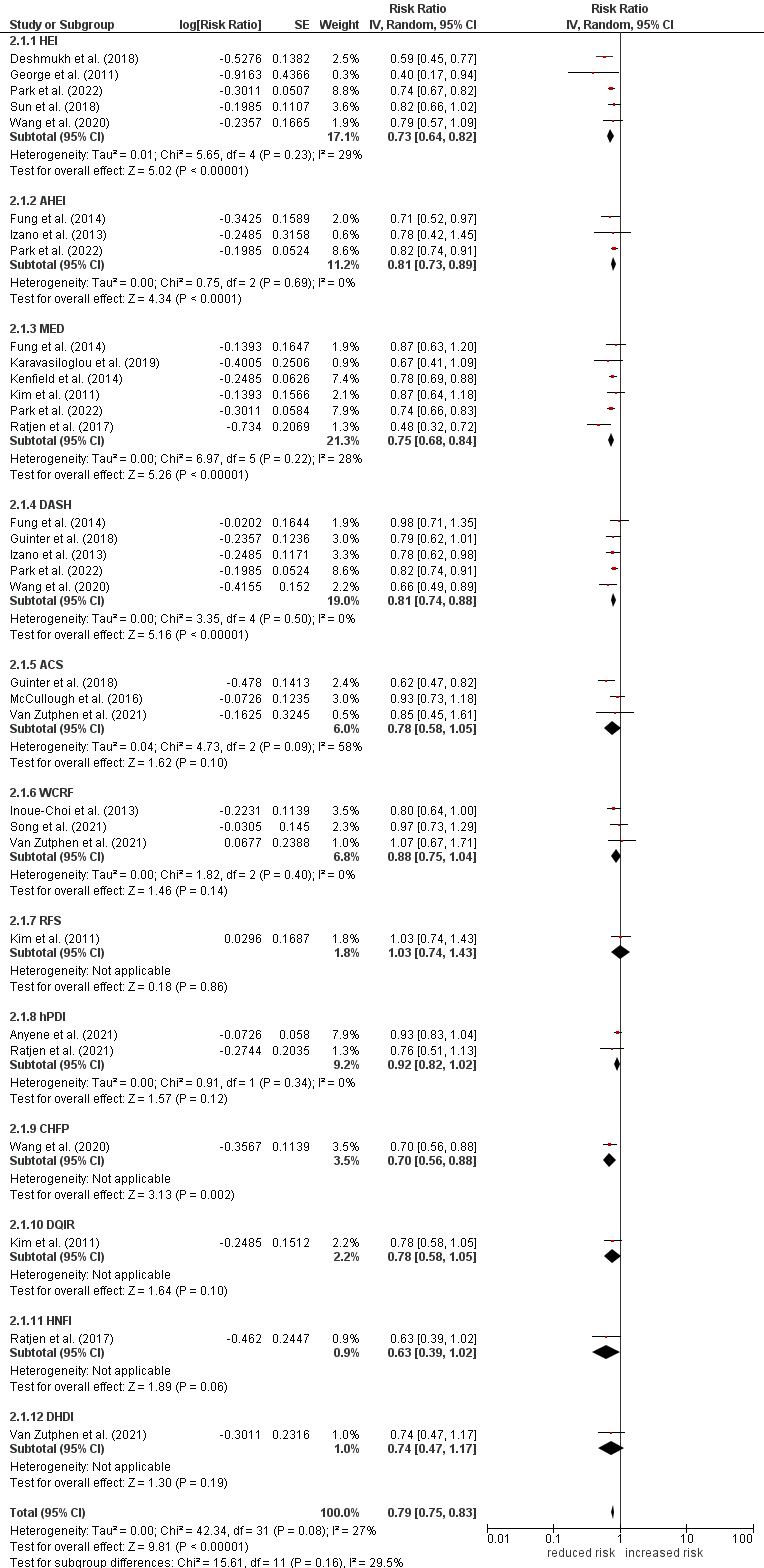


Figure S4: Forest plot showing pooled risk ratios with 95% CIs for overall mortality comparing the highest versus lowest category of postdiagnosis adherence to diet-quality indices of 18 observational studies by subgroup: type of index.

The red squares represent the point of estimates of the intervention effect, the size of the red squares represents the weight assigned to each study within the meta-analysis. The horizontal lines represent the corresponding 95% CI. The black diamonds show the effect estimates for each subgroup and the black diamond at the bottom of the graph represents the overall effect estimate of all included studies.

Abbreviations: *ACS* American Cancer Society, *AHEI* Alternative Healthy Eating Index, *CHFP* Chinese Food Pagoda, *CI* confidence interval, *DASH* Dietary Approaches to Stop Hypertension, *df* degrees of freedom, *DHDI* Dutch Healthy Diet Index, *DQIR* Diet Quality Index-Revised, *HEI* Healthy Eating Index, *HNFI* healthy Nordic Food Index, *hPDI* healthful plant-based diet index, *I²* inconsistency, *IV* inverse variance, *MED* Mediterranean Diet Score, *SE* standard error, *tau* estimate between study variance, *RFS* Recommended Food Score, *WCRF/AICR* World Cancer Research Fund/ American Institute for Cancer Research.


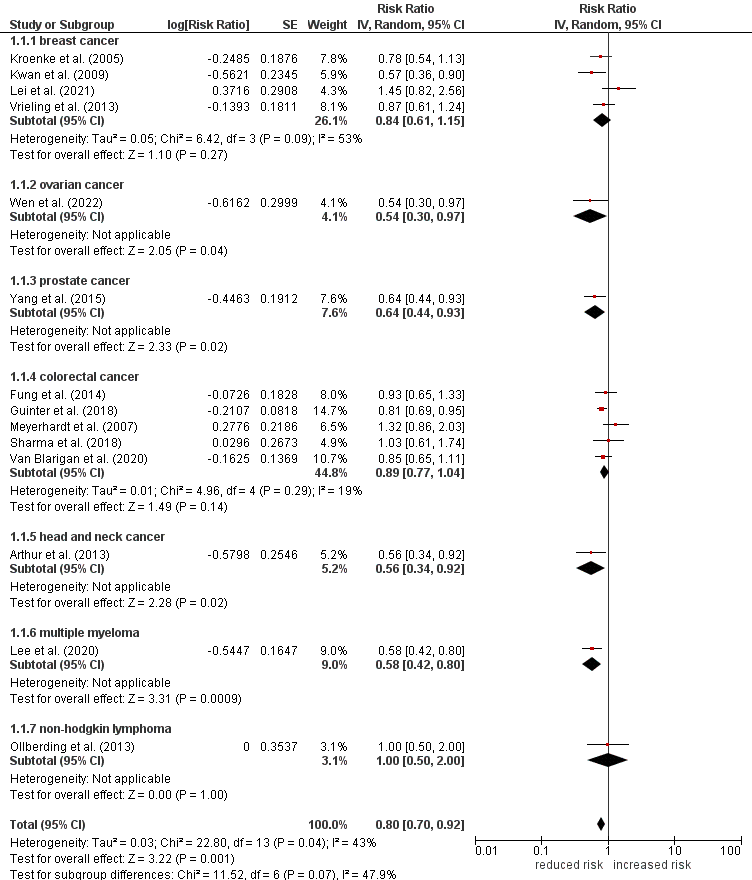


Figure S5: Forest plot showing pooled risk ratios with 95% CIs for overall mortality comparing the highest versus lowest category of adherence to a healthy/prudent dietary pattern of 14 observational studies by subgroup: type of cancer.

The red squares represent the point of estimates of the intervention effect, the size of the red squares represents the weight assigned to each study within the meta-analysis. The horizontal lines represent the corresponding 95% CI. The black diamonds show the effect estimates for each subgroup and the black diamond at the bottom of the graph represents the overall effect estimate of all included studies.

Abbreviations: *CI* confidence interval, *df* degrees of freedom, *I²* inconsistency, *IV* inverse variance, *SE* standard error, *tau* estimate between study variance.


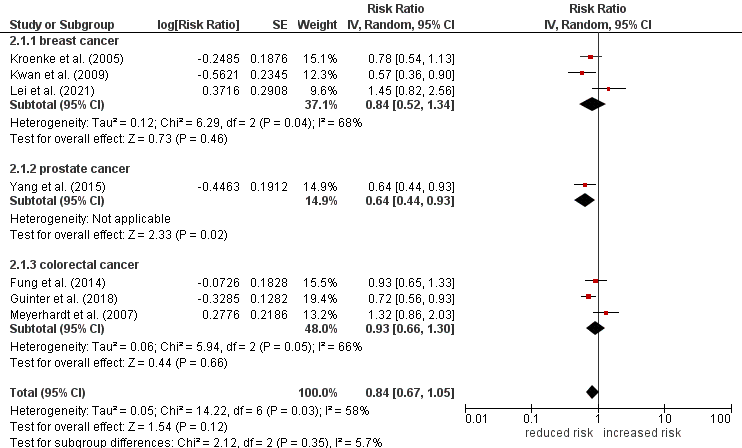


Figure S6: Forest plot showing pooled risk ratios with 95% CIs for overall mortality comparing the highest versus lowest category of postdiagnosis adherence to a healthy/prudent dietary pattern of 7 observational studies by subgroup: type of cancer.

The red squares represent the point of estimates of the intervention effect, the size of the red squares represents the weight assigned to each study within the meta-analysis. The horizontal lines represent the corresponding 95% CI. The black diamonds show the effect estimates for each subgroup and the black diamond at the bottom of the graph represents the overall effect estimate of all included studies.

Abbreviations: *CI* confidence interval, *df* degrees of freedom, *I²* inconsistency, *IV* inverse variance, *SE* standard error, *tau* estimate between study variance.


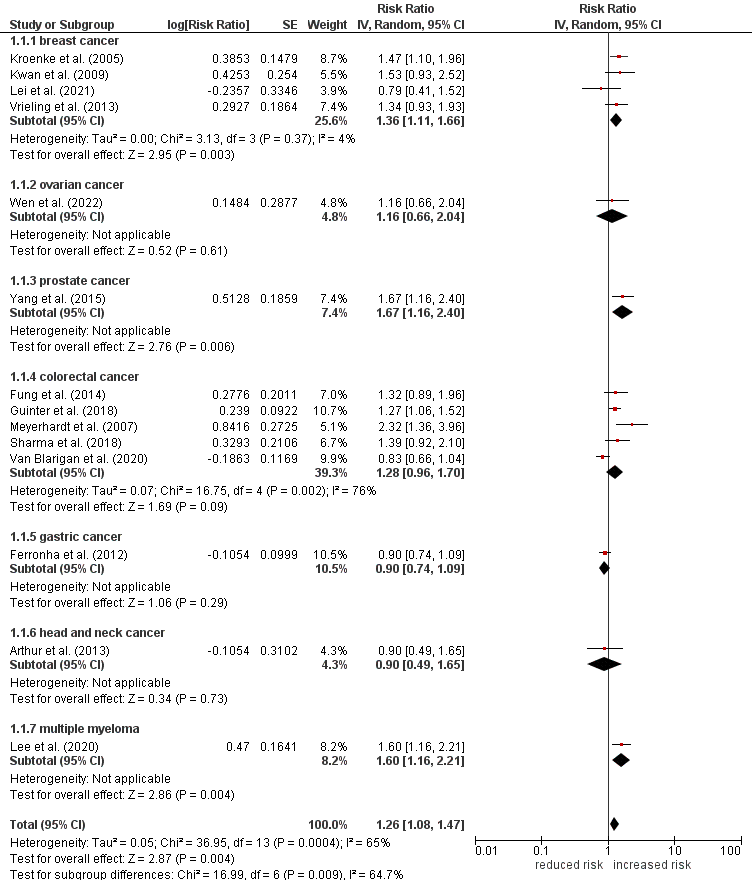


Figure S7: Forest plot showing pooled risk ratios with 95% CIs for overall mortality comparing the highest versus lowest category of adherence to an unhealthy/western dietary pattern of 14 observational studies by subgroup: type of cancer.

The red squares represent the point of estimates of the intervention effect, the size of the red squares represents the weight assigned to each study within the meta-analysis. The horizontal lines represent the corresponding 95% CI. The black diamonds show the effect estimates for each subgroup and the black diamond at the bottom of the graph represents the overall effect estimate of all included studies.

Abbreviations: *CI* confidence interval, *df* degrees of freedom, *I²* inconsistency, *IV* inverse variance, *SE* standard error, *tau* estimate between study variance.


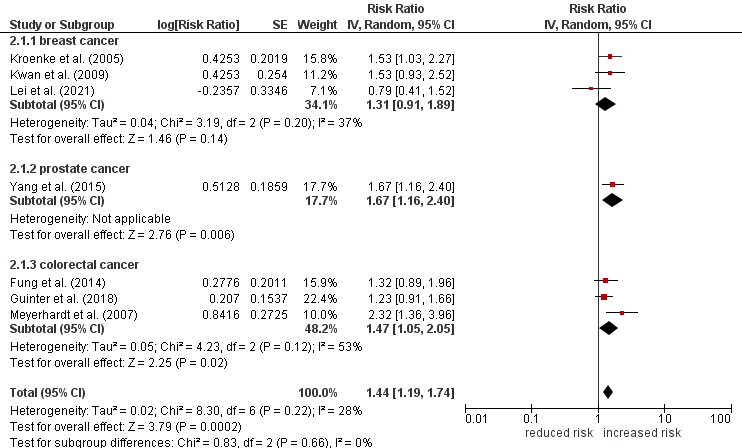


Figure S8: Forest plot showing pooled risk ratios with 95% CIs for overall mortality comparing the highest versus lowest category of postdiagnosis adherence to an unhealthy/western dietary pattern of 7 observational studies by subgroup: type of cancer.

The red squares represent the point of estimates of the intervention effect, the size of the red squares represents the weight assigned to each study within the meta-analysis. The horizontal lines represent the corresponding 95% CI. The black diamonds show the effect estimates for each subgroup and the black diamond at the bottom of the graph represents the overall effect estimate of all included studies.

Abbreviations: *CI* confidence interval, *df* degrees of freedom, *I²* inconsistency, *IV* inverse variance, *SE* standard error, *tau* estimate between study variance.


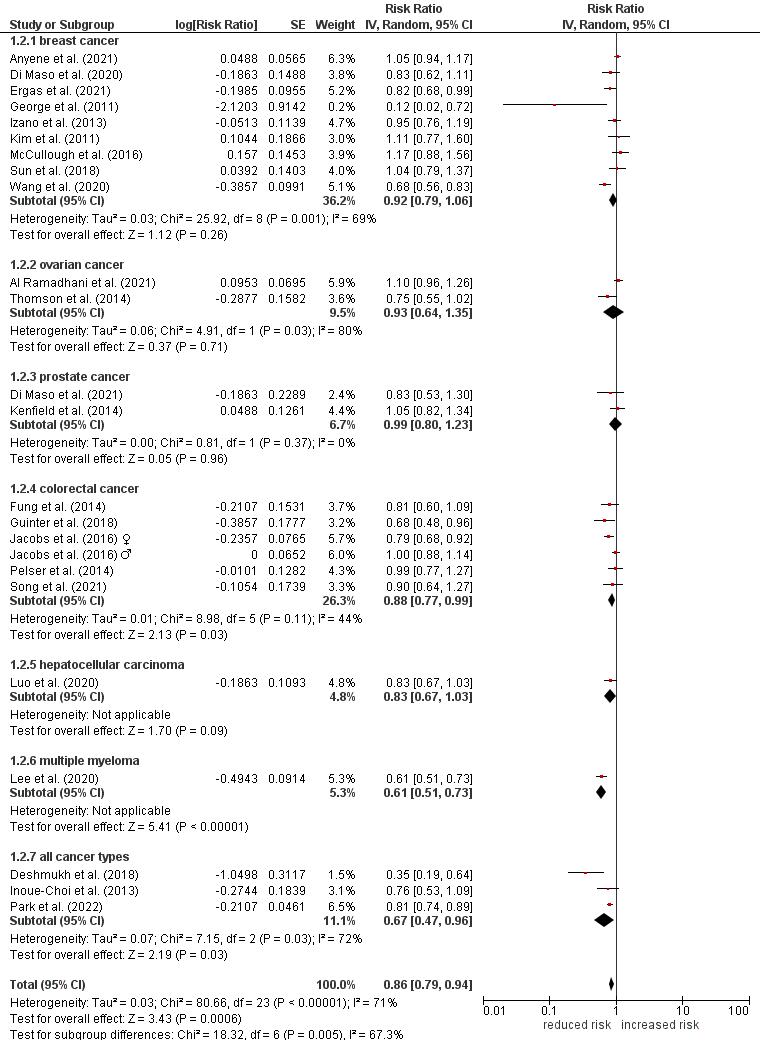


Figure S9: Forest plot showing pooled risk ratios with 95% CIs for cancer-specific mortality comparing the highest versus lowest category of adherence to diet-quality indices of 23 observational studies by subgroup: type of cancer.

The red squares represent the point of estimates of the intervention effect, the size of the red squares represents the weight assigned to each study within the meta-analysis. The horizontal lines represent the corresponding 95% CI. The black diamonds show the effect estimates for each subgroup and the black diamond at the bottom of the graph represents the overall effect estimate of all included studies.

Abbreviations: *CI* confidence interval, *df* degrees of freedom, *I²* inconsistency, *IV* inverse variance, *SE* standard error, *tau* estimate between study variance.


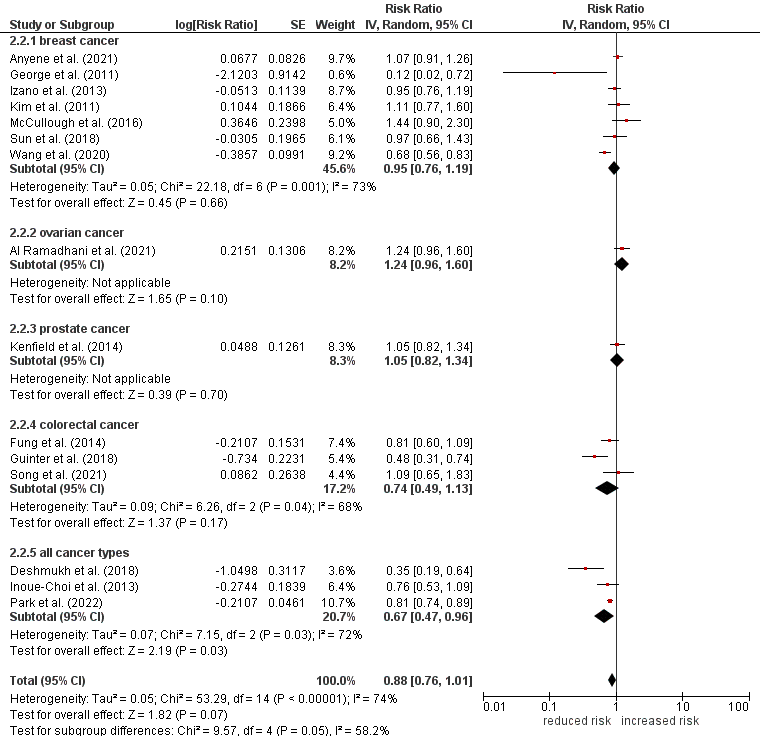


Figure S10: Forest plot showing pooled risk ratios with 95% CIs for cancer-specific mortality comparing the highest versus lowest category of postdiagnosis adherence to diet-quality indices of 15 observational studies by subgroup: type of cancer.

The red squares represent the point of estimates of the intervention effect, the size of the red squares represents the weight assigned to each study within the meta-analysis. The horizontal lines represent the corresponding 95% CI. The black diamonds show the effect estimates for each subgroup and the black diamond at the bottom of the graph represents the overall effect estimate of all included studies.

Abbreviations: *CI* confidence interval, *df* degrees of freedom, *I²* inconsistency, *IV* inverse variance, *SE* standard error, *tau* estimate between study variance.


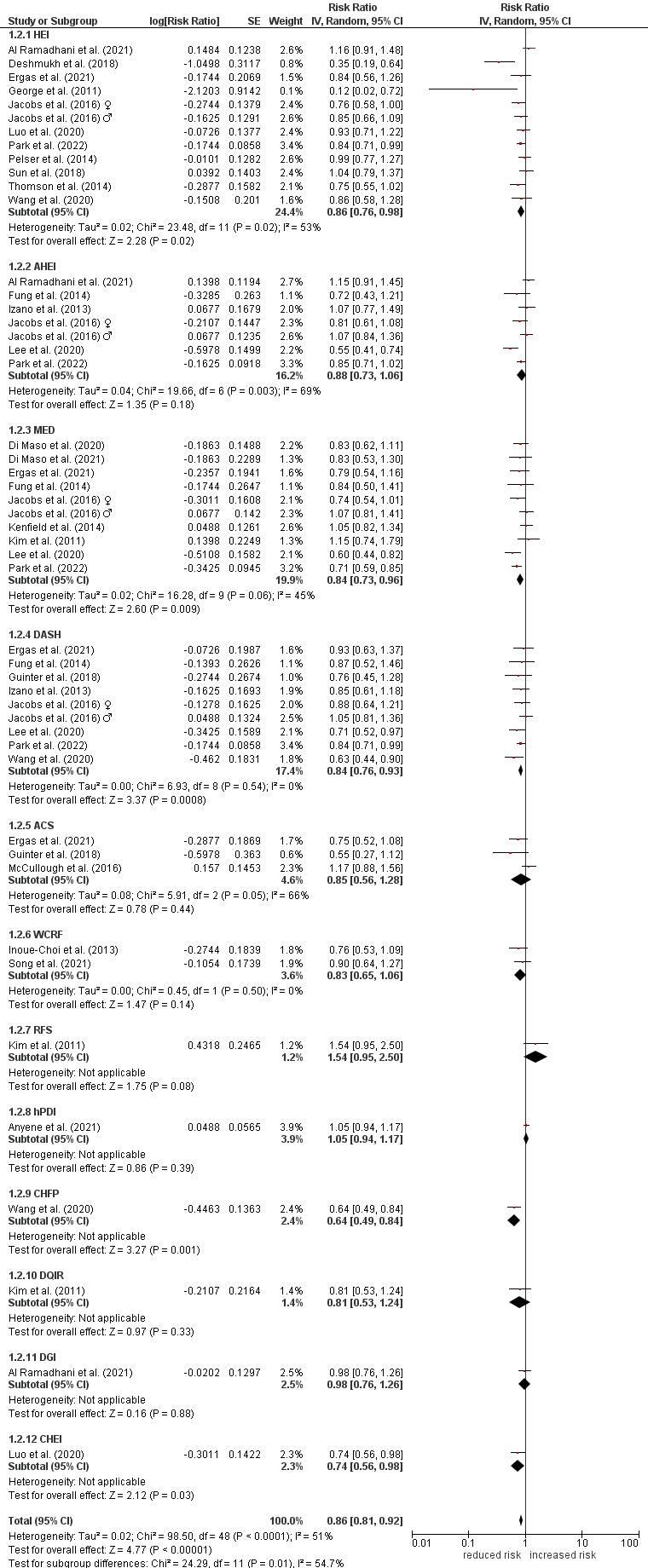


Figure S11: Forest plot showing pooled risk ratios with 95% CIs for cancer-specific mortality comparing the highest versus lowest category of adherence to diet-quality indices of 23 observational studies by subgroup: type of index.

The red squares represent the point of estimates of the intervention effect, the size of the red squares represents the weight assigned to each study within the meta-analysis. The horizontal lines represent the corresponding 95% CI. The black diamonds show the effect estimates for each subgroup and the black diamond at the bottom of the graph represents the overall effect estimate of all included studies.

Abbreviations: *ACS* American Cancer Society, *CI* confidence interval, *DASH* Dietary Approaches to Stop Hypertension, *df* degrees of freedom, *DHDI* Dutch Healthy Diet Index, *HEI* Healthy Eating Index, *hPDI* healthful plant-based diet index, *I²* inconsistency, *IV* inverse variance, *MED* Mediterranean Diet Score, *SE* standard error, *tau* estimate between study variance, *WCRF/AICR* World Cancer Research Fund/ American Institute for Cancer Research.


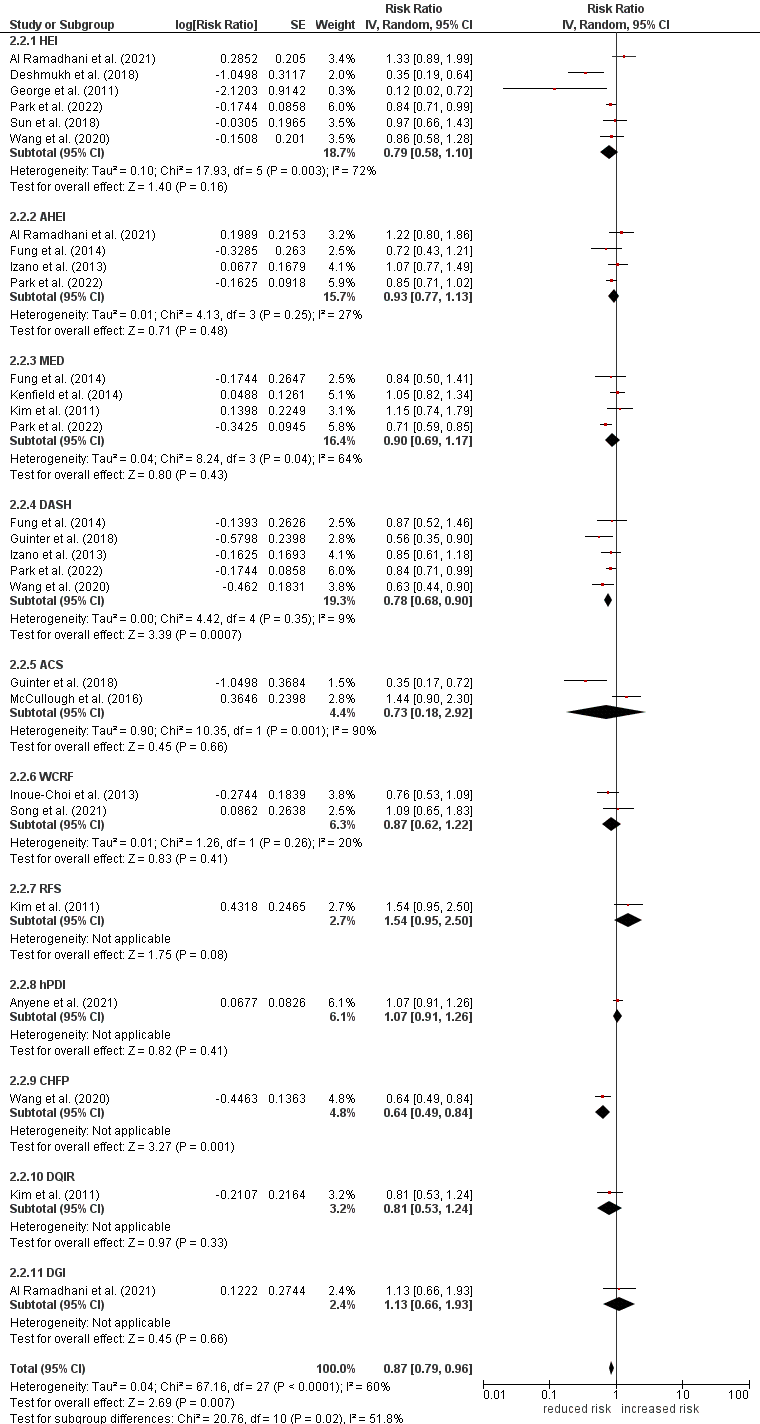


Figure S12: Forest plot showing pooled risk ratios with 95% CIs for cancer-specific mortality comparing the highest versus lowest category of postdiagnosis adherence to diet-quality indices of 15 observational studies by subgroup: type of index.

The red squares represent the point of estimates of the intervention effect, the size of the red squares represents the weight assigned to each study within the meta-analysis. The horizontal lines represent the corresponding 95% CI. The black diamonds show the effect estimates for each subgroup and the black diamond at the bottom of the graph represents the overall effect estimate of all included studies.

Abbreviations: *ACS* American Cancer Society, *CI* confidence interval, *df* degrees of freedom, *DHDI* Dutch Healthy Diet Index, *hPDI* healthful plant-based diet index, *I²* inconsistency, *IV* inverse variance, *SE* standard error, *tau* estimate between study variance, *WCRF/AICR* World Cancer Research Fund/ American Institute for Cancer Research.


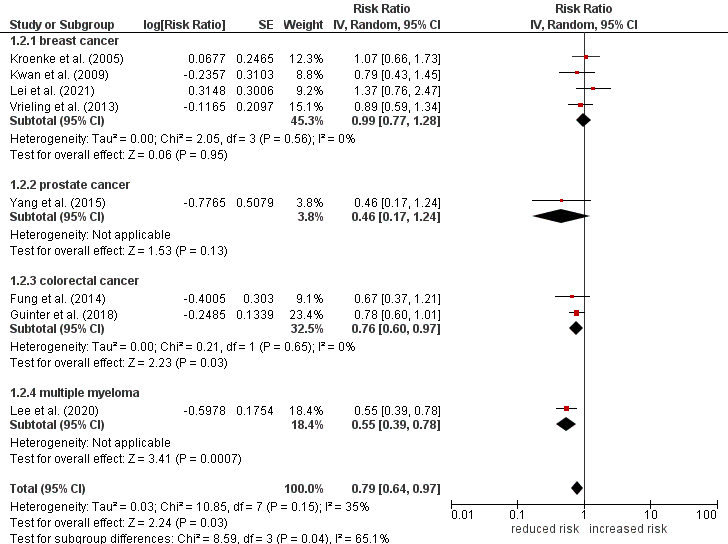


Figure S13: Forest plot showing pooled risk ratios with 95% CIs for cancer-specific mortality comparing the highest versus lowest category of adherence to a healthy/prudent dietary pattern of 8 observational studies by subgroup: type of cancer.

The red squares represent the point of estimates of the intervention effect, the size of the red squares represents the weight assigned to each study within the meta-analysis. The horizontal lines represent the corresponding 95% CI. The black diamonds show the effect estimates for each subgroup and the black diamond at the bottom of the graph represents the overall effect estimate of all included studies.

Abbreviations: *CI* confidence interval, *df* degrees of freedom, *I²* inconsistency, *IV* inverse variance, *SE* standard error, *tau* estimate between study variance.


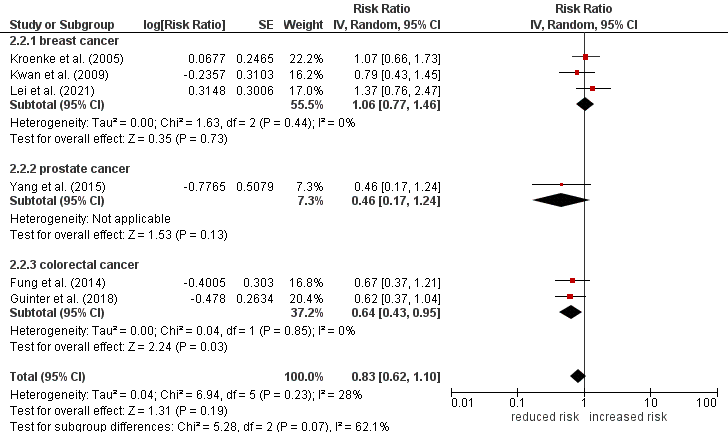


Figure S14: Forest plot showing pooled risk ratios with 95% CIs for cancer-specific mortality comparing the highest versus lowest category of postdiagnosis adherence to a healthy/prudent dietary pattern of 6 observational studies by subgroup: type of cancer.

The red squares represent the point of estimates of the intervention effect, the size of the red squares represents the weight assigned to each study within the meta-analysis. The horizontal lines represent the corresponding 95% CI. The black diamonds show the effect estimates for each subgroup and the black diamond at the bottom of the graph represents the overall effect estimate of all included studies.

Abbreviations: *CI* confidence interval, *df* degrees of freedom, *I²* inconsistency, *IV* inverse variance, *SE* standard error, *tau* estimate between study variance.


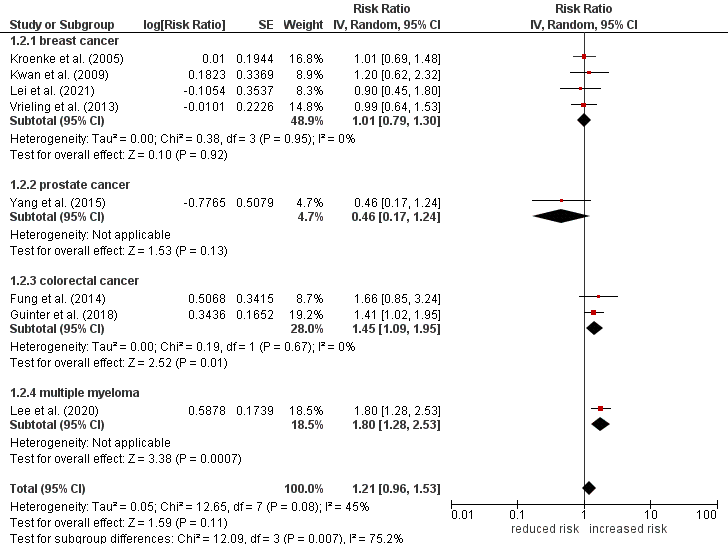


Figure S15: Forest plot showing pooled risk ratios with 95% CIs for cancer-specific mortality comparing the highest versus lowest category of adherence to an unhealthy/western dietary pattern of 8 observational studies by subgroup: type of cancer.

The red squares represent the point of estimates of the intervention effect, the size of the red squares represents the weight assigned to each study within the meta-analysis. The horizontal lines represent the corresponding 95% CI. The black diamonds show the effect estimates for each subgroup and the black diamond at the bottom of the graph represents the overall effect estimate of all included studies.

Abbreviations: *CI* confidence interval, *df* degrees of freedom, *I²* inconsistency, *IV* inverse variance, *SE* standard error, *tau* estimate between study variance.


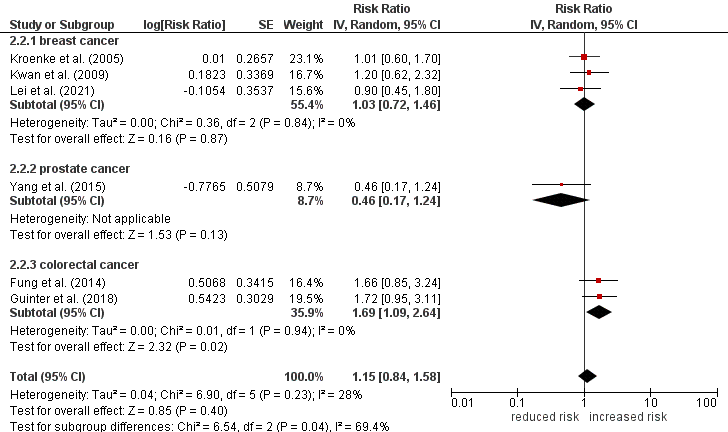


Figure S16: Forest plot showing pooled risk ratios with 95% CIs for cancer-specific mortality comparing the highest versus lowest category of postdiagnosis adherence to an unhealthy/western dietary pattern of 6 observational studies by subgroup: type of cancer.

The red squares represent the point of estimates of the intervention effect, the size of the red squares represents the weight assigned to each study within the meta-analysis. The horizontal lines represent the corresponding 95% CI. The black diamonds show the effect estimates for each subgroup and the black diamond at the bottom of the graph represents the overall effect estimate of all included studies.

Abbreviations: *CI* confidence interval, *df* degrees of freedom, *I²* inconsistency, *IV* inverse variance, *SE* standard error, *tau* estimate between study variance.


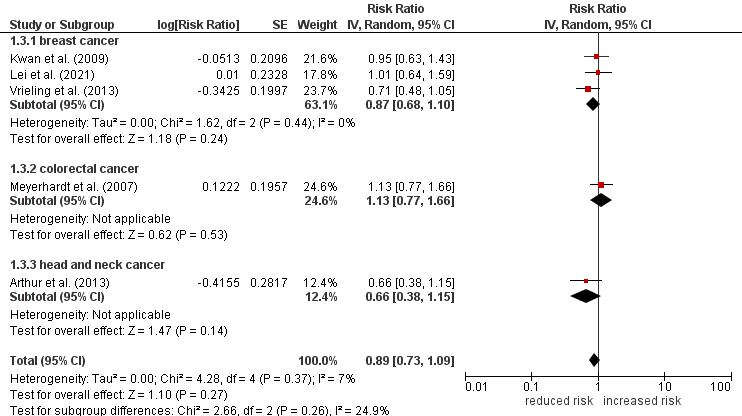


Figure S17: Forest plot showing pooled risk ratios with 95% CIs for cancer recurrence comparing the highest versus lowest category of adherence to a healthy/prudent dietary pattern of 5 observational studies by subgroup: type of cancer.

The red squares represent the point of estimates of the intervention effect, the size of the red squares represents the weight assigned to each study within the meta-analysis. The horizontal lines represent the corresponding 95% CI. The black diamonds show the effect estimates for each subgroup and the black diamond at the bottom of the graph represents the overall effect estimate of all included studies.

Abbreviations: *CI* confidence interval, *df* degrees of freedom, *I²* inconsistency, *IV* inverse variance, *SE* standard error, *tau* estimate between study variance.


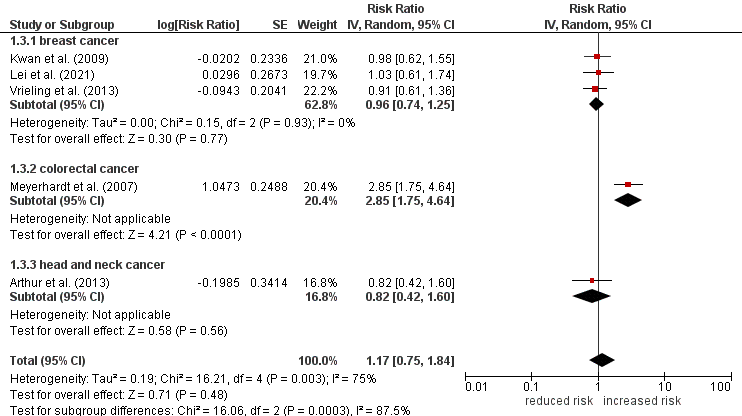


Figure S18: Forest plot showing pooled risk ratios with 95% CIs for cancer recurrence comparing the highest versus lowest category of adherence to an unhealthy/western dietary pattern of 5 observational studies by subgroup: type of cancer.

The red squares represent the point of estimates of the intervention effect, the size of the red squares represents the weight assigned to each study within the meta-analysis. The horizontal lines represent the corresponding 95% CI. The black diamonds show the effect estimates for each subgroup and the black diamond at the bottom of the graph represents the overall effect estimate of all included studies.

Abbreviations: *CI* confidence interval, *df* degrees of freedom, *I²* inconsistency, *IV* inverse variance, *SE* standard error, *tau* estimate between study variance.


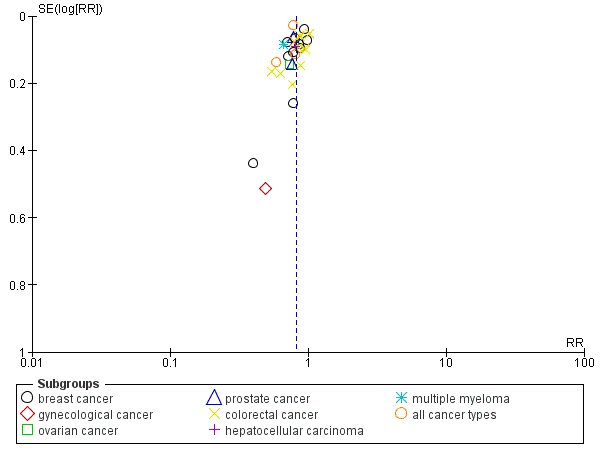


Figure S19: Funnel plot showing study precision against the relative risk with 95% CI for diet-quality indices of pre- and postdiagnosis diet (subgroup-analysis with cancer type) and overall mortality.

Abbreviations: *RR* relative risk, *SE* standard error.


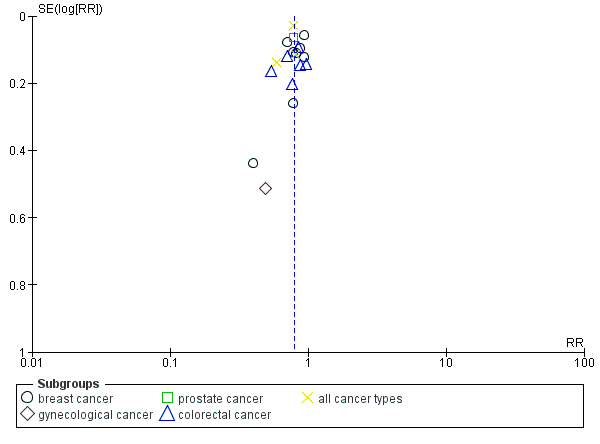


Figure S20: Funnel plot showing study precision against the relative risk with 95% CI for diet-quality indices of postdiagnosis diet (subgroup-analysis with cancer type) and overall mortality.

Abbreviations: *RR* relative risk, *SE* standard error.


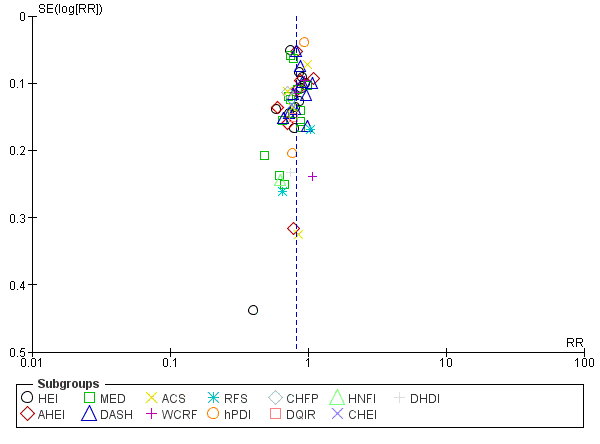


Figure S21: Funnel plot showing study precision against the relative risk with 95% CI for diet-quality indices of pre- and postdiagnosis diet (subgroup-analysis with each diet quality index) and overall mortality.

Abbreviations: *ACS* American Cancer Society, *AHEI* Alternative Healthy Eating Index, CHEI Chinese Healthy Eating Index, *CHFP* Chinese Food Pagoda, *DASH* Dietary Approaches to Stop Hypertension, *DHDI* Dutch Healthy Diet Index, *DQIR* Diet Quality Index-Revised, *HEI* Healthy Eating Index, *HNFI* healthy Nordic Food Index, *hPDI* healthful plant-based diet index, *MED* Mediterranean Diet Score, *RFS* Recommended Food Score, *RR* relative risk, *SE* standard error, *WCRF/AICR* World Cancer Research Fund/ American Institute for Cancer Research.


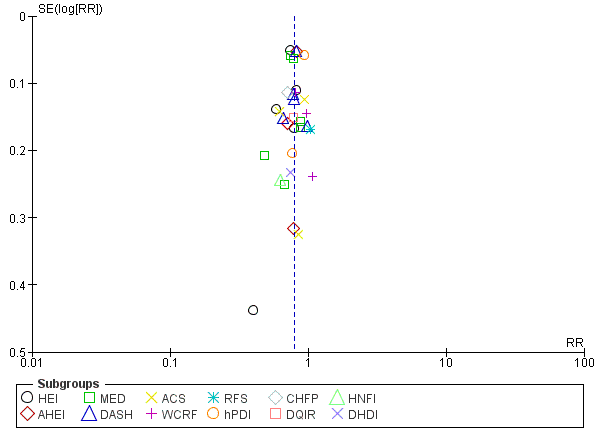


Figure S22: Funnel plot showing study precision against the relative risk with 95% CI for diet-quality indices of postdiagnosis diet (subgroup-analysis with each diet quality index) and overall mortality.

Abbreviations: *ACS* American Cancer Society, *AHEI* Alternative Healthy Eating Index, *CHFP* Chinese Food Pagoda, *DASH* Dietary Approaches to Stop Hypertension, *DHDI* Dutch Healthy Diet Index, *DQIR* Diet Quality Index-Revised, *HEI* Healthy Eating Index, *HNFI* healthy Nordic Food Index, *hPDI* healthful plant-based diet index, *MED* Mediterranean Diet Score, *RFS* Recommended Food Score, *RR* relative risk, *SE* standard error, *WCRF/AICR* World Cancer Research Fund/ American Institute for Cancer Research.


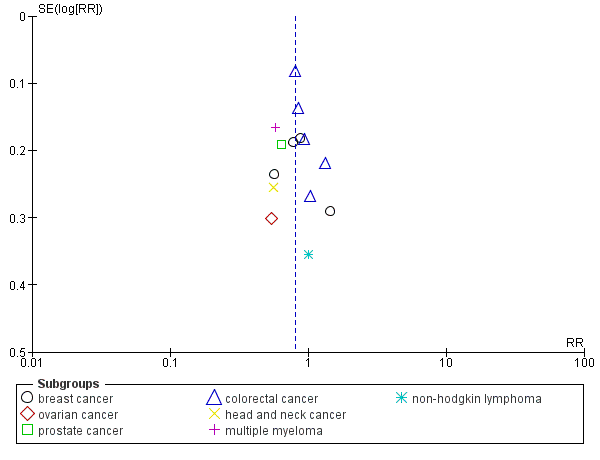


Figure S23: Funnel plot showing study precision against the relative risk with 95% CI for a healthy/prudent dietary pattern of pre- and postdiagnosis diet (subgroup-analysis with cancer type) and overall mortality.

Abbreviations: *RR* relative risk, *SE* standard error.


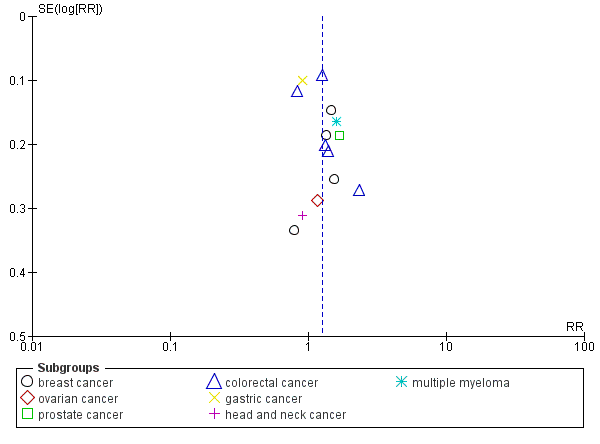


Figure S24: Funnel plot showing study precision against the relative risk with 95% CI for an unhealthy/western dietary pattern of pre- and postdiagnosis diet (subgroup-analysis with cancer type) and overall mortality.

Abbreviations: *RR* relative risk, *SE* standard error.


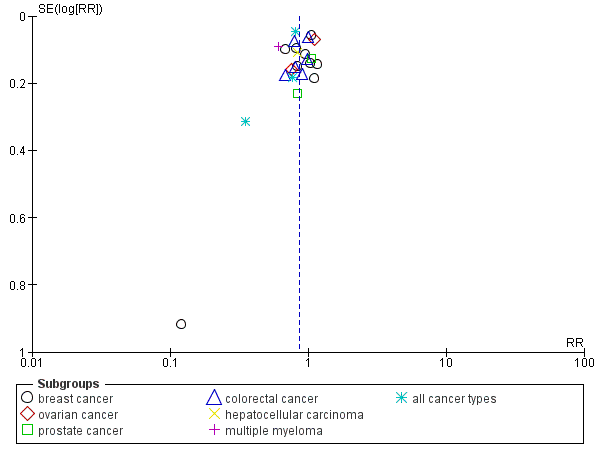


Figure S25: Funnel plot showing study precision against the relative risk with 95% CI for diet-quality indices of pre- and postdiagnosis diet (subgroup-analysis with cancer type) and cancer-specific mortality.

Abbreviations: *RR* relative risk, *SE* standard error.


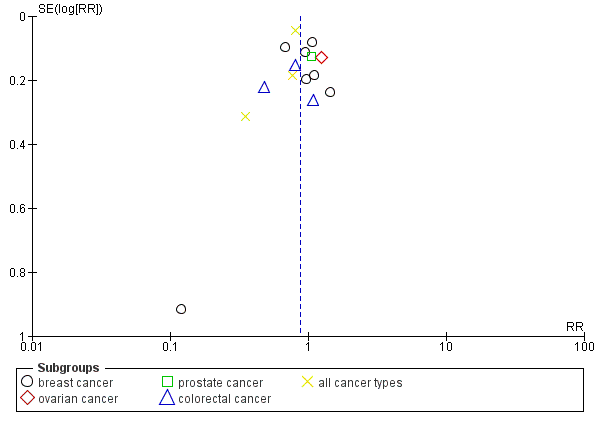


Figure S26: Funnel plot showing study precision against the relative risk with 95% CI for diet-quality indices of postdiagnosis diet (subgroup-analysis with cancer type) and cancer-specific mortality.

Abbreviations: *RR* relative risk, *SE* standard error.


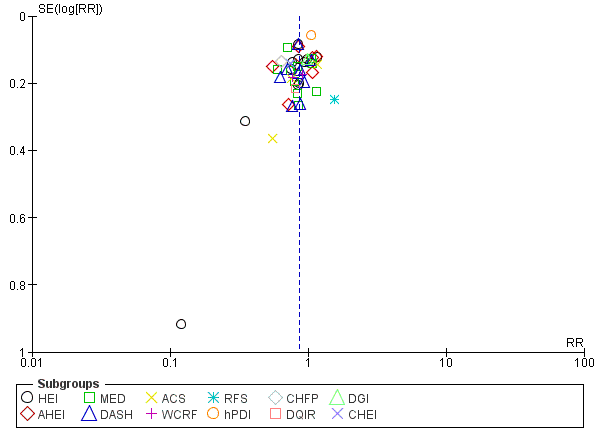


Figure S27: Funnel plot showing study precision against the relative risk with 95% CI for diet-quality indices of pre- and postdiagnosis diet (subgroup-analysis with each diet quality index) and cancer-specific mortality.

Abbreviations: *ACS* American Cancer Society, *AHEI* Alternative Healthy Eating Index, CHEI Chinese Healthy Eating Index, *CHFP* Chinese Food Pagoda, *DASH* Dietary Approaches to Stop Hypertension, *DGI* Australian Dietary Guideline Index, *DQIR* Diet Quality Index-Revised, *HEI* Healthy Eating Index, *hPDI* healthful plant-based diet index, *MED* Mediterranean Diet Score, *RFS* Recommended Food Score, *RR* relative risk, *SE* standard error, *WCRF/AICR* World Cancer Research Fund/ American Institute for Cancer Research.


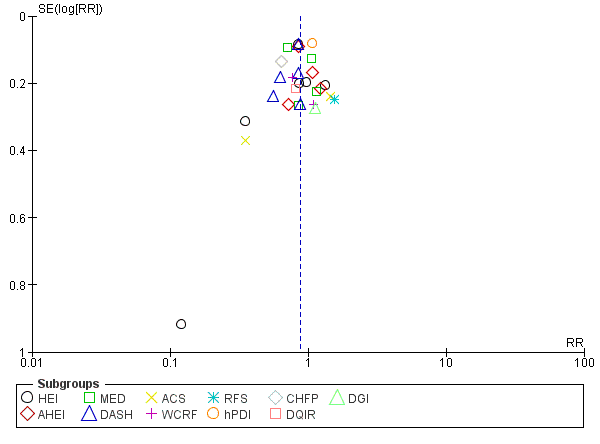


Figure S28: Funnel plot showing study precision against the relative risk with 95% CI for diet-quality indices of postdiagnosis diet (subgroup-analysis with each diet quality index) and cancer-specific mortality.

Abbreviations: *ACS* American Cancer Society, *AHEI* Alternative Healthy Eating Index, *CHFP* Chinese Food Pagoda, *DASH* Dietary Approaches to Stop Hypertension, *DGI* Australian Dietary Guideline Index, *DQIR* Diet Quality Index-Revised, *HEI* Healthy Eating Index, *hPDI* healthful plant-based diet index, *MED* Mediterranean Diet Score, *RFS* Recommended Food Score, *RR* relative risk, *SE* standard error, *WCRF/AICR* World Cancer Research Fund/ American Institute for Cancer Research.


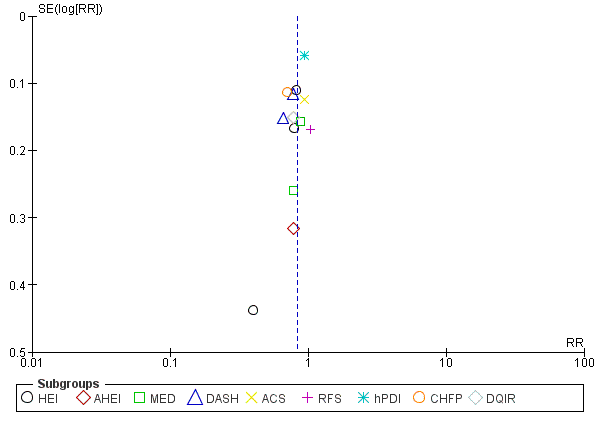


Figure S29: Funnel plot showing study precision against the relative risk with 95% CI for diet-quality indices of postdiagnosis diet (subgroup-analysis with each diet quality index) and overall mortality among breast cancer survivors.

Abbreviations: *ACS* American Cancer Society, *AHEI* Alternative Healthy Eating Index, *CHFP* Chinese Food Pagoda, *DASH* Dietary Approaches to Stop Hypertension, *DQIR* Diet Quality Index-Revised, *HEI* Healthy Eating Index, *hPDI* healthful plant-based diet index, *MED* Mediterranean Diet Score, *RFS* Recommended Food Score, *RR* relative risk, *SE* standard error.


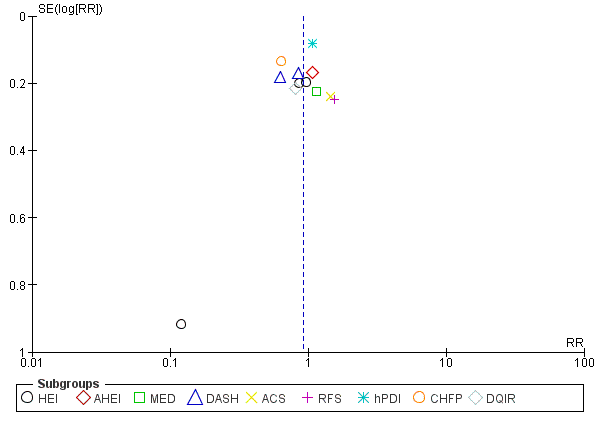


Figure S30: Funnel plot showing study precision against the relative risk with 95% CI for diet-quality indices of postdiagnosis diet (subgroup-analysis with each diet quality index) and cancer-specific mortality among breast cancer survivors.

Abbreviations: *ACS* American Cancer Society, *AHEI* Alternative Healthy Eating Index, *CHFP* Chinese Food Pagoda, *DASH* Dietary Approaches to Stop Hypertension, *DQIR* Diet Quality Index-Revised, *HEI* Healthy Eating Index, *hPDI* healthful plant-based diet index, *MED* Mediterranean Diet Score, *RFS* Recommended Food Score, *RR* relative risk, *SE* standard error.


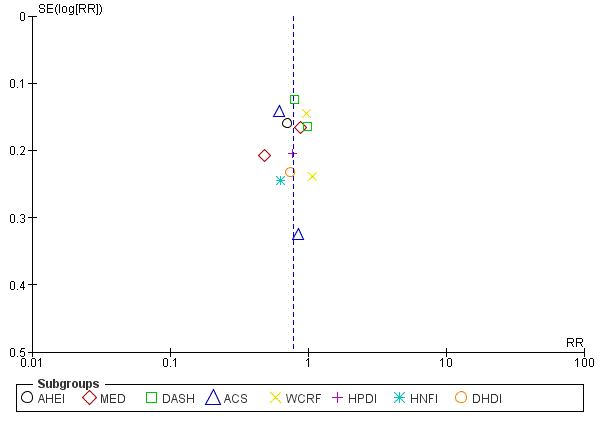


Figure S31: Funnel plot showing study precision against the relative risk with 95% CI for diet-quality indices of postdiagnosis diet (subgroup-analysis with each diet quality index) and overall mortality among colorectal cancer survivors.

Abbreviations: *AHEI* Alternative Healthy Eating Index, *DASH* Dietary Approaches to Stop Hypertension, *DHDI* Dutch Healthy Diet Index, *HNFI* healthy Nordic Food Index, *HPDI* healthful plant-based diet index, *MED* Mediterranean Diet Score, *RR* relative risk, *SE* standard error, *WCRF/AICR* World Cancer Research Fund/ American Institute for Cancer Research.
