# Supplementary material for: Association between Dietary Indices and Dietary Patterns and Mortality and Cancer Recurrence among Cancer Survivors: An Updated Systematic Review and Meta-Analysis of Cohort Studies
Source: Nutrients. 2023 Jul 14;15(14):3151. doi: 10.3390/nu15143151 (PMC10385219; doi:10.3390/nu15143151)
Supplement: Supplementary file 1 [file nutrients-15-03151-s001.zip › Trauchburg et al Manuscript_Supplementary_Material_Tables.docx]

**Table S1: Literature search strategy**

PubMed:

("diet"[All Fields] OR "dietary"[All Fields] OR "food"[All Fields] OR "foods"[All Fields] OR "mediterranean diet"[All Fields] OR "Mediterranean"[All Fields] OR "dietary pattern"[All Fields] OR "dietary score"[All Fields] OR "dietary adherence"[All Fields] OR "dash"[All Fields] OR "healthy eating"[All Fields] OR "HEI"[All Fields] OR "AHEI"[All Fields] OR "western"[All Fields] OR "prudent"[All Fields] OR "World Cancer Research Fund"[All Fields]) AND "cancer"[All Fields] AND ("survivors"[All Fields] OR "survivor"[All Fields] OR "survival"[All Fields] OR "recurrence"[All Fields]) AND ("prospective"[All Fields] OR "cohort"[All Fields] OR "longitudinal"[All Fields] OR "follow up"[All Fields] OR "case-cohort"[All Fields] OR "nested case-control"[All Fields]) AND 2016/05/18:2022/05/22[Date - Publication].

Web of Science (Web of Science Core Collection):

(((ALL=(diet OR dietary OR food OR foods OR mediterranean diet OR Mediterranean OR dietary pattern OR dietary score OR dietary adherence OR dash OR healthy eating OR HEI OR AHEI OR western OR prudent OR World Cancer Research Fund)) AND ALL=(cancer)) AND ALL=(survivors OR survivor OR survival OR recurrence)) AND ALL=(prospective OR cohort OR longitudinal OR follow up OR case-cohort OR nested case-control).

Timespan: 2016-05-18 to 2022-05-22 (Publication Date)

Table S2: Description and decision criteria for each domain in Cochrane Risk of bias in Non-randomized Studies of Interventions.

| **Domain** | **Explanation** | **Judgements** |
| --- | --- | --- |
| **Bias due to confounding** | - Is there potential for confounding of the effect of exposure in this study? - Did the authors use a multivariable-adjusted analysis method that controlled at least for age, sex, smoking, education/socioeconomic status, cancer stage, surgery/treatment and total energy intake? - Did the authors avoid adjusting for post-exposure variables?   Notes: Confounding is expected in all observational studies, low risk of bias was not assigned to any study. Time-varying confounding was expected to be unlikely and is not expected to cause risk of bias in the present study. | Low risk of bias: No bias expected due to confounding, including time-varying confounding.  Moderate risk of bias: Confounding is expected: age, sex, smoking, education/socioeconomic status, cancer stage, surgery/treatment and total energy intake have been appropriately controlled for in a multivariable-adjusted analysis  *or* confounding is expected: age, sex, smoking, cancer stage, surgery/treatment, and total energy intake have been appropriately controlled for in a multivariable-adjusted analysis and education/socioeconomic status is not expected to vary substantially within the cohort (e.g. NHS, HPFS)  *or* the authors statistically investigated whether the confounding domains have an effect on the risk estimate and excluded the confounder from the multivariable model if there was no effect on the overall effect estimate.  Serious risk of bias: At least one known important domain was not measured or appropriately controlled for  No information: No information on which confounder have been controlled for. |
| **Bias due to selection of participants** | - Was selection of participants into the study based on participants characteristics observed after start of the study/exposure assessment? - Do start of follow-up and start of exposure coincide for most participants? Were methods used that are likely to correct for the presence of selection biases?   Notes: In observational studies, it is unlikely that post-exposure variables influenced selection of participants into the study. Exclusion of participants may be mostly based on missing data, which will be considered in the domain referring to missings (see below). The start of follow-up is considered to coincide with the baseline exposure assessment. However, participants are already exposed at start of the study which might have influenced outcome measured that occurred shortly after start of the study. | Low risk of bias: All participants who would have been eligible for the target study were included in the study; and the authors excluded participants who died <1 year after diagnosis or the authors conducted a sensitivity analysis excluding participants who died <1 year after diagnosis and the results did not change.  Moderate risk of bias: Selection into the study may have been related to exposure and outcome (e.g. inclusion of postmenopausal women only); and the authors used appropriate methods to correct for the selection bias;  *or* the authors conducted no sensitivity analysis excluding participants who died <1 year after diagnosis.  Serious risk of bias: Selection into the study was related to exposure and outcome (e.g. only participants with stage IV tumor/ metastatic cancer were included in the analysis) and this could not be corrected for in the analyses;  *or* start of follow up and start of exposure do not coincide *and* the rate ratio is not constant over time.  No information: No information is reported about selection of participants into the study. |
| **Bias due to exposure assessment** | - Were exposure groups clearly defined and adequately assessed? - Was the information used to define the exposure groups based on reasonable a priori data?   Note: The start of follow-up is considered to coincide with the baseline exposure assessment. Any dietary assessment method involves measurement error, no study was assigned low risk of bias. | Low risk of bias: Exposure status was well defined (comprehensibly derived categories); *and* no measurement error is expected in its assessment.  Moderate risk of bias: Exposure status is well defined (comprehensibly derived categories); *and* exposure was measured using a validated tool (e.g. a validated FFQ).  Serious risk of bias: Exposure status is not well defined; *and* exposure was measured using not validated tools.  No information: No definition of exposure or no explanation of the source of information about exposure status is reported. |
| **Bias due to misclassification during follow-up** | - Were there deviations from the exposure beyond what would be expected in usual practice? - Were these deviations unbalanced between groups and likely to have affected the outcome?   Notes: Repeated measurements of the exposure are mostly not available in observational studies. It is not expected that there are high changes in diet in cancer participants. Changes in diet may be similar between studies and may also be similar between groups (differential misclassification is not expected). Recent studies have shown that changes in overall lifestyle (including changes in diet) occur after cancer diagnosis, but these changes are only slightly on average, even after individual consultation. Thus, if repeated measures are not available, moderate risk of bias could be assigned to a study. | Low risk of bias: Repeated measurements of the exposure status during follow-up are available. No or only slightly changes in diet quality were observed and the changes were considered in the analysis.  Moderate risk of bias: Repeated measurements of the exposure are not available, but high changes are not expected during follow-up (compare notes)  *or* repeated measurements of the exposure status during follow-up are available and some changes in lifestyle factors were observed. The analysis was appropriate to estimate the effect of changes in lifestyle factors, allowing for deviations that were likely to impact on the outcome;  Serious risk of bias: Exposure status is measured during follow-up and high changes in lifestyle factors have been observed, and the analysis was not appropriate to estimate the effect of changes in lifestyle factors, allowing for deviations that were likely to impact on the outcome.  No information: No information on deviations from the exposure is reported. |
| **Bias due to missing data** | - Were there missing outcome data? - Were participants excluded due to missing data on exposure status? - Were participants excluded due to missing data on other variables needed for analysis?   Notes: Missing data on exposure variables and other variables are expected to be missing at random and not related to exposure or outcome that have been assessed during follow-up. | Low risk of bias: Little loss-to-follow-up (<20%) and data on exposure and other variables were reasonably complete (<10% missing data) and was unlikely to introduce bias;  or the analysis addressed missing data and is likely to have removed any risk of bias.  Moderate risk of bias: There is a proportion of missing data in the original cohort or a high proportion of loss-to-follow-up; and the analysis is unlikely to have removed the risk of bias arising from the missing data (e.g. using logistic regression).  Serious risk of bias: High proportions (>50%) of missing data; and the analysis is unlikely to have removed the risk of bias arising from the missing data;  or missing data were addressed inappropriately in the analysis;  or the nature of the missing data means that the risk of bias cannot be removed through appropriate analysis.  No information: No information is reported about missing data or the potential for data to be missing. |
| **Bias due to measurement of the outcome** | - Could the outcome measure have been influenced by knowledge of the exposure status? - Were the methods of outcome assessment comparable across exposure groups? - Were any systematic error in measurement of the outcome related to exposure status?   Notes: In observational studies, it is not expected that outcome assessors were aware of exposure status of the participants. | Low risk of bias: The methods of outcome assessment were comparable across exposure groups; and the outcome measure was unlikely to be influenced by knowledge of the exposure status of study participants; and any error in measuring the outcome is unrelated to exposure status (i.e. objective measures such as confirmed medical records, record linkage).  Moderate risk of bias: The methods of outcome assessment were comparable across exposure groups; and any error in measuring the outcome may be minimally related to exposure status or if the outcome measure was not reliable measured (i.e. confirmed records are not available for the whole study population).  Serious risk of bias: The methods of outcome assessment were not comparable across exposure groups; or the outcome measure was subjective (i.e. self-report of cancer recurrence by study participants); and error in measuring the outcome was related to exposure status.  No information: No information is reported about the methods of outcome assessment. |
| **Bias due to selective reporting of the results** | - Is the reported effect estimate likely to be selected from multiple analyses of exposure-outcome relationship? - Is the reported effect estimate likely to be selected from different subgroups?   Notes: In observational studies, it is unusual to publish an a priori analysis plan or protocol. Multiple outcome measurements for the definition of cancer and individual cancer stages are not expected. | Low risk of bias: There is a clear description of all analysis and the analyses are consistent and all reported results correspond to all intended outcomes, analyses and sub-cohorts.  Moderate risk of bias: The analyses are clearly defined; and there is indication of selection of the reported analysis from among multiple analyses; and there is indication of selection of the cohort or subgroups for analysis and reporting on basis of the results (e.g. estimates not shown for all analyses).  Serious risk of bias: There is a high risk of selective reporting from among multiple analyses; or the cohort or subgroup is selected from a larger study for analysis and appears to be reported based on the results.  No information: There is too little information to make a judgement. |
| **Overall judgement** | Low risk of bias | The study is judged to be at low risk of bias for all domains. |
|  | Moderate risk of bias | The study is judged to be at low or moderate risk of bias for all domains. |
|  | Serious risk of bias | The study is judged to be at serious risk of bias in at least one domain |
|  | No information | There is no clear indication that the study is at serious risk of bias and there is a lack of information in one or more key domains of bias. |

Abbreviations: *FFQ* food frequency questionnaire, *HPFS* Health Professionals Follow-up Study, *NHS* Nurses’ Health Study.

Table S3: Full-text articles excluded.

| Study | Index includes not only diet (n=3) | Index not relevant  (hyperinsulinemia) (n=1) | exploratory factor analysis for dietary pattern (n=1) |
| --- | --- | --- | --- |
| Heitz et al. (2018) | x |  |  |
| Parada et al. (2019) | x |  |  |
| Tabung et al. (2020) |  | X |  |
| Van Blarigan et al. (2018) | x |  |  |
| Westhoff et al. (2018) |  |  | x |

Table S4: Comparison of included studies in the qualitative analysis with the same cohort.

| **Author (y)** | **Cohort** | **Cancer type** | **PRE** | **POST** | **Information for decision** | **Decision** |
| --- | --- | --- | --- | --- | --- | --- |
| Anyene et al. (2021) | Pathways Study | Breast cancer | x | x | Different indices (hPDI) | Include |
| Deshmukh et al. (2018) | NHANES III | All cancer types |  | x | HEI-1995 | Include |
| Di Maso et al. (2021) | Italian case-control study | Prostate cancer | x |  | Different cancer types | Include |
| Di Maso et al. (2020) | Italian case-control study | Breast cancer | x |  | Different cancer types | Include |
| Ergas et al. (2021) | Pathways Study | Breast cancer | x |  | Different indices (AHEI-2010, aMED, DASH) | Include |
| Fung et al. (2014) | NHS | Colorectal cancer |  | x | Different cancer types | Include |
| George et al. (2014) | WHI | Breast cancer |  | x | HEI-2005 | Complete exclusion |
| Guinter et al. (2018) | CPS-II Nutrition Cohort | Colorectal cancer | x | x | Different cancer types | Include |
| Izano et al. (2013) | NHS | Breast cancer |  | x | AHEI-2010 | Include |
| Jacobs et al. (2016) | MEC | Colorectal cancer | x |  | Prediagnosis | Include |
| Karavasiloglou et al. (2019) | NHANES III | Breast cancer and gynecological cancer |  | x | HEI-1995, MED | Exclusion of HEI, use of the other index |
| Kenfield et al. (2014) | HPFS | Prostate cancer |  | x | Different cancer types | Include |
| Kim et al. (2011) | NHS | Breast cancer |  | x | AHEI | Exclusion of AHEI,  use of the other indices |
| Kroenke et al. (2005) | NHS | Breast cancer | x | x | Different cancer types | Include |
| Lee et al. (2020) | NHS & HPFS | Multiple myeloma | x |  | Different cancer types | Include |
| McCullough et al. (2016) | CPS-II Nutrition Cohort | Breast cancer | x | x | Different cancer types | Include |
| Meyerhardt et al. (2007) | CALGB 89803 | Colon cancer |  | x | Prediagnosis | Include |
| Park et al. (2022) | MEC | All cancer types |  | x | Postdiagnosis | Include |
| Ratjen et al. (2017) | PopGen biobank | Colorectal cancer |  | x | Different indices (MED, HNFI) | Include |
| Ratjen et al. (2021) | PopGen biobank | Colorectal cancer |  | x | Different indices (hPDI) | Include |
| Song et al. (2021) | NHS & HPFS | Colorectal cancer | x | x | Different cancer types | Include |
| Sharma et al. (2018) | NLFCRC | Colorectal cancer | x |  | Prudent vegetable pattern | Include |
| Sun et al. (2018) | WHI | Breast cancer | x | x | HEI-2010 | Include |
| Van Blarigan et al. (2020) | CALGB/SWOG 80405 | Colorectal cancer | x |  | Postdiagnosis | Include |
| Zhu et al. (2013) | NLFCRC | Colorectal cancer | x |  | Prudent vegetable pattern | Complete exclusion |

Abbreviations: *AHEI* Alternative Healthy Eating Index, *aMED* alternate Mediterranean Diet Score, *CALGB* Cancer and Leukemia Group B, *CALGB/SWOG 80405* Cancer and Leukemia Group B/Southwest Oncology Group 80405, *CPS* Cancer Prevention Study, *DASH* Dietary Approaches to Stop Hypertension, *HEI* Healthy Eating Index, *HNFI* healthy Nordic Food Index, *hPDI* healthful plant-based diet index, *HPFS* Health Professionals Follow-up Study, *MEC* Multiethnic Cohort, *MED* Mediterranean Diet Score, *NHANES III* Third National Health and Nutrition Examination Survey, *NHS* Nurses’ Health Study, NLFCRC Newfoundland and Labrador Familial Colorectal Cancer cohort, *POST* postdiagnosis, *PRE* prediagnosis, *Ref* reference, *WHI* Women’s Health Initiative, *y* year.

Detailed explanations:

George et al. [1] analyzed data from the Women’s Health Initiative (WHI) cohort, as did Sun et al [2]. Both studies used the HEI and analyzed the postdiagnosis diet of breast cancer survivors. Sun et al. had a longer follow-up period of 12 years compared to 9.6 years and also examined pre-diagnosed diet. Therefore, Sun et al.104 was included. Both Sharma et al. [3] and Zhu et al. [4] investigated the association between dietary patterns and colorectal cancer survival using the data from the Newfoundland Familial Colorectal Cancer Registry with a pre-diagnosed diet. The more recent study was chosen because it investigated two dietary indices in addition to dietary patterns. Kim et al. [5] and Izano et al. [6] examined breast cancer survivors from the same cohort. Since only the index AHEI was the same and otherwise different indices were used, only AHEI from Kim et al. was not included in the quantitative analysis. The remaining indices from Kim et al. were used as Izano et al. did not examine those. Kroenke et al. [7] used the same data as the two aforementioned studies but analyzed the association with dietary patterns. The same applies to Karavasiloglou et al. [8] and Deshmukh et al. [9]. Both investigated the effect of adherence to HEI within the National Health and Nutrition Examination Survey (NHANES) cohort III among other dietary indices. Karavasiloglou et al. chose only breast and gynaecological cancer whereas Deshmukh et al did not narrow down the type of cancer. Since the latter provides a higher number of participants due to no limitation of cancer type, the HRs for adherence to HEI from Karavasiloglou et al. are not used in this meta-analysis. Furthermore, data from the Multiethnic Cohort (MEC) were used in two selected studies. Jacobs et al. [10] referred to the pre-diagnosed diet, whereas Park et al. [11] used data from the post-diagnosed diet. Therefore, both results were included in the analysis. Anyene et al. [12] provided a total of five different indices, two of them were WCRF and ACS. It was not clear whether only the diet sub-score or the total score was used for the WCRF and ACS indices. The total score also includes other components not related to diet, such as BMI or physical activity. Therefore, the WCRF and ACS indices from Anyene et al. were excluded, while the remaining indices were included in the persent meta-analysis. Finally, Kenfield et al. [13] used MED and alternate MED (aMED) as scores. The indices are similar in terms of evaluation, therefore they were merged for comparison with other dietary indices.

**References**

1. George, S.M.; Alfano, C.M.; Neuhouser, M.L.; Smith, A.W.; Baumgartner, R.N.; Baumgartner, K.B.; Bernstein, L.; Ballard-Barbash, R. Better postdiagnosis diet quality is associated with less cancer-related fatigue in breast cancer survivors. *J Cancer Surviv* **2014**, *8*, 680-687, doi:10.1007/s11764-014-0381-3.

2. Sun, Y.; Bao, W.; Liu, B.; Caan, B.J.; Lane, D.S.; Millen, A.E.; Simon, M.S.; Thomson, C.A.; Tinker, L.F.; Van Horn, L.V., et al. Changes in Overall Diet Quality in Relation to Survival in Postmenopausal Women with Breast Cancer: Results from the Women's Health Initiative. *J Acad Nutr Diet* **2018**, *118*, 1855-1863.e1856, doi:10.1016/j.jand.2018.03.017.

3. Sharma, I.; Roebothan, B.; Zhu, Y.; Woodrow, J.; Parfrey, P.S.; McLaughlin, J.R.; Wang, P.P. Hypothesis and data-driven dietary patterns and colorectal Cancer survival: findings from Newfoundland and Labrador colorectal Cancer cohort. *Nutr J* **2018**, *17*, 55, doi:10.1186/s12937-018-0362-x.

4. Zhu, Y.; Wu, H.; Wang, P.P.; Savas, S.; Woodrow, J.; Wish, T.; Jin, R.; Green, R.; Woods, M.; Roebothan, B., et al. Dietary patterns and colorectal cancer recurrence and survival: a cohort study. *BMJ Open* **2013**, *3*, doi:10.1136/bmjopen-2012-002270.

5. Kim, E.H.; Willett, W.C.; Fung, T.; Rosner, B.; Holmes, M.D. Diet quality indices and postmenopausal breast cancer survival. *Nutr Cancer* **2011**, *63*, 381-388, doi:10.1080/01635581.2011.535963.

6. Izano, M.A.; Fung, T.T.; Chiuve, S.S.; Hu, F.B.; Holmes, M.D. Are diet quality scores after breast cancer diagnosis associated with improved breast cancer survival? *Nutr Cancer* **2013**, *65*, 820-826, doi:10.1080/01635581.2013.804939.

7. Kroenke, C.H.; Fung, T.T.; Hu, F.B.; Holmes, M.D. Dietary patterns and survival after breast cancer diagnosis. *J Clin Oncol* **2005**, *23*, 9295-9303, doi:10.1200/JCO.2005.02.0198.

8. Karavasiloglou, N.; Pestoni, G.; Faeh, D.; Rohrmann, S. Post-Diagnostic Diet Quality and Mortality in Females with Self-Reported History of Breast or Gynecological Cancers: Results from the Third National Health and Nutrition Examination Survey (NHANES III). *Nutrients* **2019**, *11*, doi:10.3390/nu11112558.

9. Deshmukh, A.A.; Shirvani, S.M.; Likhacheva, A.; Chhatwal, J.; Chiao, E.Y.; Sonawane, K. The Association Between Dietary Quality and Overall and Cancer-Specific Mortality Among Cancer Survivors, NHANES III. *JNCI Cancer Spectr* **2018**, *2*, pky022, doi:10.1093/jncics/pky022.

10. Jacobs, S.; Harmon, B.E.; Ollberding, N.J.; Wilkens, L.R.; Monroe, K.R.; Kolonel, L.N.; Le Marchand, L.; Boushey, C.J.; Maskarinec, G. Among 4 Diet Quality Indexes, Only the Alternate Mediterranean Diet Score Is Associated with Better Colorectal Cancer Survival and Only in African American Women in the Multiethnic Cohort. *J Nutr* **2016**, *146*, 1746-1755, doi:10.3945/jn.116.234237.

11. Park, S.Y.; Kang, M.; Shvetsov, Y.B.; Setiawan, V.W.; Boushey, C.J.; Haiman, C.A.; Wilkens, L.R.; Le Marchand, L. Diet quality and all-cause and cancer-specific mortality in cancer survivors and non-cancer individuals: the Multiethnic Cohort Study. *Eur J Nutr* **2022**, *61*, 925-933, doi:10.1007/s00394-021-02700-2.

12. Anyene, I.C.; Ergas, I.J.; Kwan, M.L.; Roh, J.M.; Ambrosone, C.B.; Kushi, L.H.; Cespedes Feliciano, E.M. Plant-Based Dietary Patterns and Breast Cancer Recurrence and Survival in the Pathways Study. *Nutrients* **2021**, *13*, doi:10.3390/nu13103374.

13. Kenfield, S.A.; DuPre, N.; Richman, E.L.; Stampfer, M.J.; Chan, J.M.; Giovannucci, E.L. Mediterranean diet and prostate cancer risk and mortality in the Health Professionals Follow-up Study. *Eur Urol* **2014**, *65*, 887-894, doi:10.1016/j.eururo.2013.08.009.

Table S5: Characteristics of the cohort studies included in the present meta-analysis.

| **Author (y)** | **Country; Cohort name** | **Outcome** | **Population (n); Follow-up (y)** | **Sex; Age at diagnosis (y);** | **Tumor  characteristics** | **Exposure assessment; Timeframe;**  **Recurrence assessment** | **Components of score;**  **Score range** | **Adjustment** | **RR/HR (95% CI) Multivariable adjusted** |
| --- | --- | --- | --- | --- | --- | --- | --- | --- | --- |
| Al Ramadhani et al. (2021) | Australia  OPAL | Ovarian cancer survival | Pre-diagnosis: 650; Post-diagnosis: 503  4.4 | W  18-79 (age at study entry of OPAL) | Ovarian cancer | FFQ completed prediagnosis and 12 months post-diagnosis  Prediagnosis; Postdiagnosis  Assessment of recurrence: collected annually from medical records | **HEI-2010** 1. ↑ total fruit, whole fruit, total vegetables, dark green vegetables and legumes, seafood and plant proteins, total protein foods (each 0-5 points); 2. ↑ whole grains, dairy, PUFA and MUFA:SFA ratio (each 0-10 points); 3. ↓ refined grains, sodium (each 0-10 points); 4. ↓ empty calories (0-20 points).  Score range: 0-100  **AHEI-2010** 1. ↑ vegetables (excluding potatoes); 2. ↑ whole fruit; 3. ↑ whole grains; 4. ↑ nuts and legumes; 5. ↑ LCFA n-3; 6. ↑ PUFA; 7. ↓ sugar-sweetened beverages; 8. ↓ red/processed meat; 9. ↓ sodium; 10. ↓ trans fat; 11. ↔ alcohol. For each food group 0 to 10 points.  Score range: 0-110  **DASH** 1. ↑ vegetables; 2. ↑ nuts and legumes; 3. ↑ fruit; 4. ↑ whole grains; 5. ↑ low-fat dairy; 6. ↓ sodium; 7. ↓ red/processed meat; 8. ↓ sweetened beverages (for each 1-5 points).  Score range: 8-40 points  **DGI** 1. ↑ food variety; 2. ↑ vegetables; 3. ↑ fruit; 4. ↑ grain (cereal) foods (total cereal intake; wholegrain); 5. ↑ lean meat and poultry, fish, eggs, nuts and seeds, legumes/beans (total meat and alternative; lean meats:total meat ratio); 6. ↑ milk, yoghurt, cheese and/or their alternatives; 7. ↑ Drink plenty of water (Total beverage intake; water:total beverage ratio);  8. ↓ foods containing saturated fat, added salt, added sugars and alcohol; 9. ↓ foods high in saturated fat; 10. ↓ unsaturated oils, fats or spreads; 11. ↓ salt; 12. ↓ added sugar; 13. ↔ alcohol (for each 0-10 points).  Score rang: 0-130 | Prediagnosis: age, log energy, body mass index, smoking status. Stratified by diabetes and physical activity.  Postdiagnosis: age, log energy, smoking status at 12 months, FIGO stage. Stratified by physical activity at 12 months. | Prediagnosis *ovarian cancer survival* **HEI-2010:** HR 1.08 (0.80, 1.48);  **AHEI-2010:** HR 1.12 (0.84, 1.51); **DGI:** HR 0.94 (0.70, 1.28);   Postdiagnosis *ovarian cancer survival* **HEI-2010:** HR 1.33 (0.89, 2.01);  **AHEI-2010:** HR 1.22 (0.80, 1.84); **DGI:** HR 1.13 (0.66, 1.91);   Third vs. first tertile |
| Anyene et al. (2021) | USA  Pathways Study | Overall mortality, Breast cancer mortality, Non-breast cancer mortality, Cancer recurrence | 3,646  9.51 | W  60±12 | Breast cancer | FFQ (diet from the last 6 months) at baseline, 6, 24 and 72 months later prediagnosis: only the first FFQ was used postdiagnosis: cumulative average score of the repeated FFQs, if there was no follow-up FFQ, baseline FFQ was assumed to be constant over the study period  Prediagnosis; Postdiagnosis  Assessment of recurrence: Follow-up health status questionnaires and KPNC electronic medical record searches | **PDI:** 1. ↑ whole grains, fruits, vegetables, nuts, legumes, vegetable oils, tea, coffee;  2. ↑ fruit juices, refined grains, potatoes, sugar-sweetened beverages, sweets and desserts;  3. ↓ dairy, animal fat, egg, meat, fish or seafood, miscellaneous animal-based foods (Pizza, cream soups like chowders, mayonnaise, and sandwich spreads).  **hPDI:** 1 ↑ whole grains, fruits, vegetables, nuts, legumes, vegetable oils, tea, coffee;  2. ↓ fruit juices, refined grains, potatoes, sugar-sweetened beverages, sweets and desserts;  3. ↓ dairy, animal fat, egg, meat, fish or seafood, miscellaneous animal-based foods.  **uPDI:** 1. ↓ whole grains, fruits, vegetables, nuts, legumes, vegetable oils, tea, coffee;  2. ↑ fruit juices, refined grains, potatoes, sugar-sweetened beverages, sweets and desserts;  3. ↑ dairy, animal fat, egg, meat, fish or seafood, miscellaneous animal-based foods.  total serving size consumption for each food group was broken into cohort-specific quintiles, and each quintile was given a score between 1 and 5.   Score range: 18-90 points | Age at diagnosis, total energy intake, physical activity, race/ethnicity, education, menopausal status, smoking status. Stratified by tumor stage and ER status. | Prediagnosis:  *Overall mortality* **PDI:** HR 1 (0.87, 1.14);  **hPDI:** HR 0.94 (0.85, 1.05);  **uPDI:** HR 1.06 (0.96, 1.18);   *Breast cancer mortality* **PDI:** HR 1.03 (0.85, 1.24);  **hPDI:** HR 1.03 (0.88, 1.19);  **uPDI:** HR 0.97 (0.84, 1.13);   *Non-breast cancer mortality* **PDI:** HR 0.92 (0.76, 1.11);  **hPDI:** HR 0.88 (0.76, 1.02);  **uPDI:** HR 1.14 (0.98, 1.32);   *Cancer recurrence* **PDI:** HR 1.13 (0.97, 1.33);  **hPDI:** HR 1.05 (0.92, 1.19);  **uPDI:** HR 0.96 (0.84, 1.09);   Postdiagnosis:  *Overall mortality* **PDI:** HR 0.96 (0.82, 1.11);  **hPDI:** HR 0.93 (0.83, 1.05);  **uPDI:** HR 1.07 (0.96, 1.2);   *Breast cancer mortality* **PDI:** HR 0.98 (0.79, 1.22);  **hPDI:** HR 1.07 (0.91, 1.25);  **uPDI:** HR 0.94 (0.8, 1.1);   *Non-breast cancer mortality* **PDI:** HR 0.90 (0.73, 1.11);  **hPDI:** HR 0.83 (0.71, 0.98);  **uPDI:** HR 1.20 (1.02, 1.41);  *Cancer recurrence* **PDI:** HR 1.17 (0.98, 1.39);  **hPDI:** HR 1.11 (0.97, 1.26);  **uPDI:** HR 0.9 (0.79, 1.03);   Per 10-unit increase |
| Arthur et al. (2013) | USA  UM HN-SPORE | Overall mortality, Cancer recurrence | 542  6.0 | M/W  59±11  (at study entry) | Head and neck squamous cell carcinoma | FFQ (diet over the past year) shortly after diagnosis  Prediagnosis  Assessment of recurrence: abstracted from medical record review | *Principal component analysis* **Whole-foods pattern:** ↑ vegetables, fruit, legumes, fish, poultry, whole grains, fruit juice, olive oil, nuts, garlic.  **Western pattern:** ↑ red and processed meats, refined grains, French fries, potatoes, condiments, high-fat dairy products, margarine, butter, eggs, coffee, desserts, snacks, mayonnaise, regular beverages.  Score range: PCA | Age, sex, tumor site, cancer stage, treatment, ACE-27 comorbidities, smoking, BMI, total energy intake. | *Overall mortality* **Whole-foods:** HR 0.56 (0.34, 0.92); **Western:** HR 0.90 (0.49, 1.68);  *Cancer recurrence* **Whole-foods:** HR 0.66 (0.38, 1.16); **Western:** HR 0.82 (0.42, 1.61);  Fifth vs. first quintile |
| Deshmukh et al. (2018) | USA  NHANES III | Overall mortality, Cancer-specific mortality | 1,191  17.2 | M/W  ≥ 18 | All types of cancer | Single 24-hour dietary recall  Postdiagnosis | **HEI** (version of 1994–1996) ↑ grain, vegetable, fruit, meat, dairy, variety; ↔ total fat; ↓ saturated fat, cholesterol, sodium, (for each 0-10 points). A score of 0 is assigned for zero servings, and the maximum score indicates that the recommended servings were consumed.  Score range: 0-100 | Age, sex, income, education, body mass index, comorbidities. | All cancer patients *Overall mortality* **HEI:** HR 0.59 (0.45, 0.77);  *Cancer-specific mortality* **HEI:** HR 0.35 (0.19, 0.63);  Fourth vs. first quartile |
| Di Maso et al. (2020) | Italy  Italian case-control study | Overall mortality, Breast cancer mortality, Non-breast cancer mortality | 1,453  12.6 | W  23-78 (at study entry) | Breast cancer | FFQ (diet two years prior to diagnosis)  Prediagnosis | **MDS** (based on MED) 1. ↑ cereals; 2. ↑ fruit; 3. ↑ vegetables; 4. ↑ legumes; 5. ↑ fish; 6. ↑ MUFA/SFA ratio; 7. ↓ dairy products; 8. ↓ meat; 9. ↔ alcohol. 1 point for intake was greater/less than the cohort specific median.  Score range: 0-9 points | Area of residence at diagnosis, calendar period of cancer diagnosis, age at diagnosis, years of education, menopausal status, TNM stage, estrogen/ progesterone receptor status, total energy intake. Cause-specific mortality was further adjusted for competing risk according to Fine-Gray model. | *Overall mortality* **MDS:** HR 0.72 (0.57, 0.92);   *Breast cancer mortality* **MDS:** HR 0.83 (0.62, 1.11);   *Non-breast cancer mortality* **MDS:** HR 0.58 (0.36, 0.93);   Third vs. first tertile |
| Di Maso et al. (2021) | Italy  Italian case-control study | Prostate cancer mortality, Non-prostate cancer mortality | 777  10 | M  46-74 (at study entry) | Prostate cancer | FFQ (diet two years prior to diagnosis)  Prediagnosis | **MDS** (based on MED) 1. ↑ cereals; 2. ↑ fruit; 3. ↑ vegetables; 4. ↑ legumes; 5. ↑ fish; 6. ↑ MUFA/SFA ratio; 7. ↓ dairy products; 8. ↓ meat; 9. ↔ alcohol. 1 point for intake was greater/less than the cohort specific median.  Score range: 0-9 points | Age at diagnosis, years of education, Gleason score, abdominal obesity, smoking habits, total energy intake. | *Prostate cancer mortality* **MDS:** HR 0.83 (0.53, 1.31);   *Non-prostate cancer mortality* **MDS:** HR 0.73 (0.51, 1.05);   High vs. low adherence |
| Ergas et al. (2021) | USA  Pathways Study | Overall mortality, Breast cancer mortality, Non-breast cancer mortality, Cancer recurrence | 3,660  40,888 person-years of follow-up | W  24-94 | Breast cancer | FFQ at baseline average of 2.3 months after diagnosis  Prediagnosis  Assessment of recurrence: interviews with participants or from monthly algorithmic searches of Kaiser Permanente Northern, California electronic databases and confirmed by medical chart review | **ACS** 1. ↑ fruits/vegetables (servings/d, variety); 2. ↑ whole grains as a percentage of total grains; 3. ↓ total red and processed meat (for each 0-3 points). Score range: 0-9 points  **aMED** 1. ↑ total vegetables (excluding potatoes); 2. ↑ total fruit; 3. ↑ nuts; 4. ↑ legumes; 5. ↑ fish; 6. ↑ whole grains; 7. ↑ MUFA/SFA ratio; 8. ↔ alcohol; 9. ↓ red and processed meat. 1 point for intake was greater/less than the cohort specific median.   Score range: 0-9  **DASH** 1. ↑ total vegetables (excluding potatoes and legumes); 2. ↑ nuts and legumes; 3. ↑ total fruit; 4. ↑ whole grains; 5. ↑ low-fat dairy; 6. ↓ sodium; 7. ↓ red and processed meat; 8. ↓ sweetened beverages (for each 1-5 points).  Score range: 8-40 points  **HEI-2015** 1. ↑ total fruits; 2. ↑ whole fruits; 3. ↑ total vegetables; 4. ↑ greens and beans; 5. total protein; 6. ↑ seafood and plant protein; (each 0-5 points) 7. ↑ whole grain; 8. ↑ dairy; 9. ↑ (PUFA + MUFA)/SFAs ratio; 10. ↓ refined grains; 11. ↓ sodium; 12. ↓ added sugar; 13. ↓ saturated fat (each 0-10 points, calculated per 1000 kcal or percentage of energy).  Score range: 0-100 | Age at diagnosis, total energy, race and ethnicity, education, menopausal status, physical activity, smoking, cancer stage, estrogen-receptor status, progesterone-receptor status, HER2, body mass index, surgery type, chemotherapy, radiation, hormonal therapies. | *Overall mortality* **ACS:** HR 0.77 (0.59, 1.01);  **aMED:** HR 0.87 (0.66, 1.14);  **DASH:** HR 0.80 (0.61, 1.05);  **HEI:** HR 0.81 (0.62, 1.06);  *Breast cancer mortality* **ACS:** HR 0.75 (0.52, 1.09);  **aMED:** HR 0.79 (0.54, 1.16);  **DASH:** HR 0.93 (0.63, 1.39);  **HEI:** HR 0.84 (0.56, 1.27);   *Non-breast cancer mortality* **ACS:** HR 0.69 (0.48, 0.98);  **aMED:** HR 0.73 (0.50, 1.05);  **DASH:** HR 0.55 (0.38, 0.79);  **HEI:** HR 0.67 (0.48, 0.94);   *Cancer recurrence* **ACS:** HR 1.19 (0.89, 1.57);  **aMED:** HR 1.08 (0.79, 1.47);  **DASH:** HR 1.02 (0.73, 1.41);  **HEI:** HR 1.24 (0.88, 1.75);   Fifth vs. first quantile |
| Ferronha et al. (2012) | Portugal  Patients of Hospital de S. João and Instituto Português de  Oncologia Francisco Gentil | Overall mortality | 568  6.6 (for those alive at end)  1.2 (for those who died) | M/W  63 (median; at study entry) | Gastric cancer | FFQ (dietary intake referred to the previous year or the year before onset of symptoms)  Prediagnosis | *Principal component analysis* **Pattern 1:** ↑ dairy, fruits, salads and vegetables, ↓ meat and alcoholic beverages. **Pattern 2:** ↓ coffee, fish and seafood, fruits, salads and vegetables, sugar and sweets. **Pattern 3:** ↑ alcoholic beverages, cereals and potatoes, eggs, fast-foods/fried snacks, soft drinks, meat, salads and vegetables.  Score range: PCA | Age, sex, education, extent of disease (grouped in localised and local spread; regional spread; advanced; unknown), total energy intake. | *Overall mortality* **Pattern 2:** HR 0.94 (0.75; 1.19); **Pattern 3:** HR 0.79 (0.55; 1.15);  Pattern I was the reference |
| Fung et al. (2014) | USA  NHS | Overall mortality,  Colorectal cancer mortality | 1,201  11.2 | W  61-72 | Colorectal cancer (stages I to III) | FFQ at least 6 months after diagnosis  Postdiagnosis | **AHEI-2010** 1. ↑ vegetables (excluding potatoes); 2. ↑ whole fruit; 3. ↑ whole grains; 4. ↑ nuts and legumes; 5. ↑ LCFA n-3; 6. ↑ PUFA; 7. ↓ sugar-sweetened beverages; 8. ↓ red/processed meat; 9. ↓ sodium; 10. ↓ trans fat; 11. ↔ alcohol. For each food group 0 to 10 points.  Score range: 0-110  **DASH** 1. ↑ fruit; 2. ↑ vegetables; 3. ↑ nuts and legumes; 4. ↑ low-fat dairy products; 5. ↑ whole grains; 6. ↓ red/processed meats; 7. ↓ sweets; 8. ↓ sodium.  For healthy food groups: 1 point for the lowest quintile, 2 points for the next intake quintile, 5 points for the highest quintile. Scoring was reversed for unfavorable unhealthy food groups.  Score range: 0-40  **aMED** 1. ↑ vegetables; 2. ↑ legumes; 3. ↑ fruits; 4. ↑ nuts; 5. ↑ whole grains; 6. ↑ fish; 7. ↑ MUFA:SFA ratio; 8. ↓ meat; 9. ↔ alcohol. 1 point for intake was greater/less than the cohort-specific median.   Score range: 0-9  *Principal component analysis* **Prudent pattern:** ↑ fruits, vegetables, whole grains, poultry, low fat dairy products.  **Western pattern:** ↑ red and processed meats, refined grains, sweets and desserts, high fat dairy products.  Score range: PCA | Age, physical activity, BMI, weight change, cancer grade, chemotherapy, smoking status, energy intake, colon or rectal cancer, stage of disease, and date of colorectal cancer diagnosis DASH: additionally adjusted for alcohol. | *Overall mortality* **AHEI:** HR 0.71 (0.52, 0.98); **DASH:** HR 0.98 (0.71, 1.35); **aMED:** HR 0.87 (0.63, 1.21);  **Prudent:** HR 0.93 (0.65, 1.34);  **Western:** HR 1.32 (0.89, 1.97);   *Colorectal cancer mortality* **AHEI:** HR 0.72 (0.43, 1.21);  **DASH:** HR 0.87 (0.52, 1.45);  **aMED:** HR 0.84 (0.50, 1.42);  **Prudent:** HR 0.67 (0.37, 1.22);  **Western:** HR 1.66 (0.85, 3.23);   Fifth vs. first quintile |
| George et al. (2011) | USA  HEAL | Overall mortality, Cancer-specific mortality | 670  6 | W  >18 | Breast cancer | FFQ (diet of the previous year or month) at the 30-month postdiagnosis assessment  Postdiagnosis | **HEI-2005** 1. ↑ total fruit, whole fruit (not juice), total vegetables, dark green and orange vegetables and legumes, total grains, whole grains (each 0-5 points); 2. ↑ milk, meat and beans, oils (each 0-10 points); 3. ↓ SFA, sodium (each 0-10 points); 4. ↓ calories from solid fats, alcoholic beverages, added sugars (0-20 points).  Score range: 0-100 | Energy intake, physical activity, race, stage, tamoxifen use, body mass index. | *Overall mortality* **HEI-2005:** HR 0.40 (0.17, 0.94);  *Cancer-specific mortality* **HEI-2005:** HR 0.12 (0.02, 0.99);  Fourth vs. first quartile |
| Guinter et al. (2018) | USA  CPS-II Nutrition Cohort | Overall mortality, Colorectal cancer mortality, Non-colorectal cancer mortality | Pre-diagnosis: 2,671  6.5 (among participants who died); 11.4 (among participants who were alive at the end of the study);  Post-diagnosis: 1,321  6.4 (among participants who died); 13.5 (among participants who were alive at the end of the study) | M/W  73±7.0; (pre-diagnosis: 73.6±7.01; post-diagnosis: 70.6±6.06) | Colorectal cancer | FFQ (diet of the previous year) at baseline in 1992/1993 and/or 1999 or 2003  Prediagnosis; Postdiagnosis | **DASH** 1. ↑ vegetables; 2. ↑ nuts and legumes; 3. ↑ fruit; 4. ↑ whole grains; 5. ↑ low-fat dairy; 6. ↓ sodium; 7. ↓ red/processed meat; 8. ↓ sweetened beverages (for each 1-5 points).  Score range: 8-40 points  **ACS** 1.↑ fruits/vegetables (servings/d, variety); 2. ↑ whole grains:refined grains ratio; 3. ↓ red/processed meat (for each 0-3 points).  Score range: 0-9 points  *Principal component analysis* **Prudent pattern:**  Prediagnosis: ↑ citrus fruits, melons and berries, other fruits, dark green vegetables, cruciferous vegetables, red/orange vegetables, tomatoes, other vegetables, whole grains, beans and legumes. Postdiagnosis: ↑ other fruits, dark green vegetables, cruciferous vegetables, red/orange vegetables, tomatoes, other vegetables, poultry, other fish (not fried), beans and legumes, condiments.  **Western pattern:** Prediagnosis: ↑ starchy vegetables, refined grains, red meat (beef, lamb, pork, etc), cured/processed meats, eggs, solid fats, salty snacks.  Postdiagnosis: ↑ starchy vegetables, refined grains, red meat (beef, lamb, pork, etc), cured/processed meats, eggs, high-fat dairy, solid fats.  Score range: PCA | Age at diagnosis, year of diagnosis, sex, stage, total caloric intake, body mass index, education, smoking status, change in weight since 1992, treatment. | Prediagnosis: *Overall mortality* **DASH:** HR 0.92 (0.78, 1.08);  **ACS:** HR 0.78 (0.65, 0.95);  **Prudent:** HR 0.85 (0.73, 1.00);  **Western:** HR 1.30 (1.03, 1.64);   *Colorectal cancer mortality* **DASH:** HR 0.97 (0.74, 1.28);  **ACS:** HR 0.74 (0.54, 1.03);  **Prudent:** HR 0.85 (0.64, 1.13);  **Western:** HR 1.30 (0.88, 1.91);   *Non-colorectal cancer mortality* **DASH:** HR 0.88 (0.69, 1.12);  **ACS:** HR 0.80 (0.60, 1.06);  **Prudent:** HR 0.82 (0.64, 1.05);  **Western:** HR 1.63 (1.14, 2.31);   Postdiagnosis: *Overall mortality* **DASH:** HR 0.79 (0.62, 0.99);  **ACS:** HR 0.62 (0.47, 0.83);  **Prudent:** HR 0.72 (0.56, 0.93);  **Western:** HR 1.23 (0.91, 1.65);   *Colorectal cancer mortality* **DASH:** HR 0.56 (0.35, 0.89);  **ACS:** HR 0.35 (0.17, 0.73);  **Prudent:** HR 0.62 (0.37, 1.06);  **Western:** HR 1.72 (0.95, 3.12);   *Non-colorectal cancer mortality* **DASH:** HR 0.91 (0.65, 1.29);  **ACS:** HR 0.59 (0.39, 0.89);  **Prudent:** HR 0.67 (0.47, 0.97);  **Western:** HR 1.10 (0.72, 1.68);  Fourth vs. first quartile |
| Inoue-Choi et al. (2013) | USA  IWHS | Overall mortality, Cancer-specific mortality | 2,017  5.4 | W  72-88 (age at follow-up, when FFQ was assessed) | Breast, colorectal, gynecologic and other cancers | FFQ (diet after diagnosis)  Postdiagnosis | **WCRF/AICR-2007 (diet only)** 1. ↓ sugar beverage; 2. ↑ fruit and vegetable; 3. ↑ dietary fiber; 4. ↓ red meat and processed meat; 5. ↓ alcohol; 6. ↓ sodium.  For each food group 0 to 1 point.  Score range: 0-6 | Age, total number of comorbid conditions (accumulated, 1986–2004), perceived general health and current smoking, cancer stage, cancer type, cancer treatment (surgery, chemotherapy), subsequent cancer diagnosis before 2004, current cancer treatment, person years since cancer diagnosis, physical activity, body weight. | *Overall mortality* **WCRF/AICR-2007:** HR 0.80 (0.64, 1.00);  *Cancer-specific mortality* **WCRF/AICR-2007:** HR 0.76 (0.53, 1.09);  < 4.0 points vs. >4.0 points |
| Izano et al. (2013) | USA  NHS | Breast cancer mortality, Non-breast cancer mortality | 4,103  112 months | W  DASH:  Q1: 60.2±8.3 Q5: 60.4±8.2  AHEI-2010:  Q1: 60.4±8.6 Q5: 60.4±8.3 | Breast cancer (stages I–III) | FFQ at least 12 months after diagnosis  Postdiagnosis | **AHEI-2010** 1. ↑ vegetables (excluding potatoes); 2. ↑ whole fruit; 3. ↑ whole grains; 4. ↑ nuts and legumes; 5. ↑ LCFA n-3; 6. ↑ PUFA; 7. ↓ sugar-sweetened beverages; 8. ↓ red/processed meat; 9. ↓ sodium; 10. ↓ trans fat; 11. ↔ alcohol. For each food group 0 to 10 points.  Score range: 0-110  **DASH** 1. ↑ fruit; 2. ↑ vegetables; 3. ↑ nuts and legumes; 4. ↑ low-fat dairy products; 5. ↑ whole grains; 6. ↓ red/processed meats; 7. ↓ sweets; 8. ↓ sodium.  For healthy food groups: 1 point for the lowest quintile, 5 points for the highest quintile. Scoring was reversed for unfavorable unhealthy food groups.  Score range: 8-40 | Age at diagnosis, quintiles of energy intake, body mass index, body mass index change, age at first birth and parity, oral contraceptive use, menopausal status and HRT use, smoking, stage of disease, radiation treatment, chemotherapy and hormonal treatment, physical activity;  Stratified by time since diagnosis (months) | *Breast cancer mortality* **AHEI-2010:** RR 1.07 (0.77, 1.49); **DASH:** RR 0.85 (0.61, 1.19);  *Non-breast cancer mortality* **AHEI-2010:** RR 0.57 (0.42, 0.77); **DASH:** RR 0.72 (0.53, 0.99);  Fifth vs. first quintile |
| Jacobs et al. (2016) | USA  MEC | Overall mortality, Colorectal cancer mortality | 4,204  6.0 | M/W  71.4±8.7 | Colorectal cancer | FFQ (of the past year) at cohort entry  Prediagnosis | **HEI-2010** 1. ↑ total fruit, whole fruit, total vegetables, dark green vegetables and legumes, seafood and plant proteins, total protein foods (each 0-5 points);  2. ↑ whole grains, dairy, PUFA and MUFA:SFA ratio (each 0-10 points); 3. ↓ refined grains, sodium (each 0-10 points); 4. ↓ empty calories (0-20 points).  Score range: 0-100  **AHEI-2010** 1. ↑ total vegetables (excluding potatoes); 2. ↑ whole fruit; 3. ↑ whole grains; 4. ↓ sugar-sweetened beverages and fruit juice; 5. ↑ nuts and legumes; 6. ↓ trans fat; 7. ↑ LCFA n-3 (EPA + DHA); 8. ↑ PUFA; 9. ↓ sodium; 10. ↔ alcohol; 11. ↓ red and processed meat. For each 0-10 points.  Score range: 0-110  **aMED** 1. ↑ total vegetables (excluding potatoes); 2. ↑ total fruit; 3. ↑ nuts; 4. ↑ legumes; 5. ↑ fish; 6. ↑ whole grains; 7. ↑ MUFA:SFA ratio; 8. ↔ alcohol; 9. ↓ red and processed meat. 1 point for intake was greater/less than the cohort-specific median.   Score range: 0-9  **DASH** 1. ↑ total vegetables (excluding potatoes); 2. ↑ total fruit; 3. ↑ nuts, seeds, legumes; 4. ↑ low-fat dairy; 5. ↑ whole grains; 6. ↓ sodium; 7. ↓ sugar-sweetened beverages and fruit juices; 8. ↓ red and processed meat. Each 1-8 points.  Score range: 8-40 | Age at diagnosis, ethnicity, stage at diagnosis, total energy intake, smoking status, pack-years, physical activity, education, radiation treatment, chemotherapy, NSAID use, family history of colorectal cancer, comorbidities. | *Overall mortality* ♂ **HEI-2010:** HR 0.91 (0.76, 1.09); ♀ **HEI-2010:** HR 0.89 (0.72, 1.09); ♂ **AHEI-2010:** HR 1.08 (0.90, 1.28); ♀ **AHEI-2010:** HR 0.83 (0.67, 1.03); ♂ **aMED:** HR 0.99 (0.81, 1.22); ♀ **aMED:** HR 0.74 (0.58, 0.94); ♂ **DASH:** HR 1.06 (0.87, 1.28); ♀ **DASH:** HR 0.97 (0.77, 1.22);  *Colorectal cancer mortality* ♂ **HEI-2010:** HR 0.85 (0.66, 1.08); ♀ **HEI-2010:** HR 0.76 (0.58, 1.01); ♂ **AHEI-2010:** HR 1.07 (0.84, 1.36); ♀ **AHEI-2010:** HR 0.81 (0.61, 1.07); ♂ **aMED:** HR 1.07 (0.81, 1.42); ♀ **aMED:** HR 0.74 (0.54, 1.01); ♂ **DASH:** HR 1.05 (0.81, 1.37); ♀ **DASH:** HR 0.88 (0.64, 1.19);  Fourth vs. first quartile |
| Karavasiloglou et al. (2019) | USA  NHANES III | Overall mortality | 230  16 | W  44.0±1.3 | Breast or gynecological cancer | 24-hour dietary recall interview mean time 10.4 years between diagnosis and questionnaire  Postdiagnosis | **HEI** (version of 1994–1996) ↑ grain, vegetable, fruit, milk, meat, variety; ↔ total fat; ↓ saturated fat, cholesterol, sodium, (for each 0-10 points). A score of 0 is assigned for zero servings, and the maximum score indicates that the recommended servings were consumed.  Score range: 0-100  **MDS** (based on MED) 1. ↑ legumes; 2. ↑ vegetables; 3. ↑ fruit and nuts; 4. ↑ cereals; 5. ↑ fish and seafood; 6. ↓ meat and meat products; 7. ↓ dairy products; 8. ↓ MUFA:SFA ratio; 9. ↔ alcohol. 1 point for intake was greater/less than the cohort specific median.   Score range: 0-9 points | Age at completion of the NHANES III questionnaire, race/ethnicity, time between diagnosis and completion of the NHANES III questionnaire, body mass index, marital status, socioeconomic status, history of menopausal hormone therapy use, smoking status, self-reported prevalent chronic diseases at baseline, daily energy consumption, moderate to vigorous physical activity, alcohol consumption (only for HEI). | *Overall mortality* Total study population **HEI:** HR 0.43 (0.29, 0.64);  **MDS:** HR 0.67 (0.41, 1.11);   Breast cancer **HEI:** HR 0.49 (0.25, 0.97);  **MDS:** 0.78 (0.47, 1.32);   Gynecological cancer **HEI:** HR 0.20 (0.10, 0.43);  **MDS:** HR 0.49 (0.18, 1.37);   High vs. low adherence |
| Kenfield et al. (2014) | USA  HPFS | Overall mortality, Prostate cancer mortality | 4,538  9.1 | M  Tertile 1: 69.0 Tertile 3: 69.7 | Prostate cancer | FFQ at baseline and updated every 4 years; (cumulative average post-diagnostic dietary intakes from the most recent FFQ preceding was calculated)  Postdiagnosis | **MED** 1. ↓ dairy and meat; 2. ↔ alcohol; 3. ↑ vegetables; 4. ↑ legumes; 5. ↑ fruits and nuts; 6. ↑ grains; 7. ↑ fish; 8. ↑ PUFA:SFA ratio. 1 point for intake was greater/less than the cohort-specific median.  Score range: 0-9  **aMED** Slightly modified from the traditional score to separate fruits and nuts into separate categories, eliminate the dairy category, include only whole-grain products in the cereal category, and use a ratio of MUFA:SFA for the fat ratio. | Age at diagnosis, time period, time since diagnosis to FFQ, energy, body mass index, vigorous physical activity, smoking status, clinical stage, Gleason score, treatment. | *Overall mortality* **MED:** HR 0.78 (0.67, 0.90); **aMED:** HR 0.78 (0.64, 0.94);  *Prostate cancer mortality* **MED:** HR 1.01 (0.75, 1.38); **aMED:** HR 1.14 (0.73, 1.76);  **MED:** Third vs. first tertile **aMED:** Fifth vs. first quintile |
| Kim et al. (2011) | USA  NHS | Overall mortality, Breast cancer mortality, Non-breast cancer mortality | 2,729  Follow-up time not shown; diagnosed between 1978 and 1998, followed through 2004 | W  30-55 (age at study entry of NHS) | Breast cancer (stages I–III) | FFQ at least 12 months after diagnosis  Postdiagnosis  Assessment of recurrence:  by questionnaires (validation by reviewing the medical records of 39 women with stage III disease) | **AHEI** 1. ↑ vegetables (excluding potatoes); 2. ↑ fruit; 3. ↑ nuts and soy; 4. ↑ cereal fiber; 5. ↑ ratio of white to red meat; 6. ↑ PUFA:SFA ratio; 7. ↓ trans fat; 8. ↓ alcohol; (each 0-10 points) 9. Duration of multivitamin use (for ≥ 5 years 7.5 points, <5 years 2.5 points).  Score range: 2.5-87.5  **RFS** 1. fruits; 2. vegetables; 3. whole grains; 4. low saturated fat proteins; 5. low fat dairy products. 1 point for each recommended food that was eaten weekly.  Score range: 0-56  **DQIR** 1. ↑ grains; 2. ↑ vegetables; 3. ↑ fruits; 4. ↓ total fat; 5. ↓ saturated fat; 6. ↓ cholesterol; 7. ↑ iron; 8. ↑ calcium; 9. ↑ diet diversity; 10. ↓ added fat and sugar moderation. Each component 0-10 points; 10 based on the recommended range of intakes.  Score range: 0-100  **aMED** 1. ↑ vegetables (excluding potatoes); 2. ↑ legumes; 3. ↑ fruits; 4. ↑ nuts; 5. ↑ whole grains; 6. ↑ fish; 7. ↑ MUFA:SFA ratio; 8. ↓ meat and dairy; 9. ↔ alcohol. 1 point for intake was greater/less than the cohort-specific median.  Score range: 0-9 | Time since diagnosis, age, alcohol intake (only for RFS), energy, multivitamin use (except for AHEI), BMI, weight change, oral contraceptive use, age, smoking status, physical activity, stage, categories of treatment, age at first birth and parity, menopausal status, postmenopausal hormone use. | *Overall mortality* **AHEI:** RR 0.85 (0.63, 1.17); **DQIR:** RR 0.78 (0.58, 1.07); **RFS:** RR 1.03 (0.74, 1.42);  **aMED:** RR 0.87 (0.64, 1.17);   *Breast cancer mortality* **AHEI:** RR 1.53 (0.98, 2.39); **DQIR:** RR 0.81 (0.53, 1.24);  **RFS:** RR 1.54 (0.95, 2.47);  **aMED:** RR 1.15 (0.74, 1.77);  *Non-breast cancer mortality* **AHEI:** RR 0.52 (0.32, 0.83);  **DQIR:** RR 0.85 (0.54, 1.34);  **RFS:** RR 0.86 (0.54, 1.37);  **aMED:** RR 0.80 (0.50, 1.26);   Fifth vs. first quintile |
| Kroenke et al. (2005) | USA  NHS | Overall mortality, Breast cancer mortality, Non-breast cancer mortality | 2,619 (2,524 diet before diagnosis)  9 | W  Prudent pattern: Q1: 57 Q5: 58 Western pattern: Q1: 59 Q5: 57 (mean age) | Breast cancer | FFQ at least 1 year after diagnosis (diet before diagnosis: FFQ within 4 years before diagnosis)  Prediagnosis; Postdiagnosis | *Principal component analysis* **Prudent pattern:** ↑ fruits, vegetables, whole grains, legumes, poultry, fish.  **Western pattern:** ↑ refined grains, processed and red meats, desserts, high-fat dairy products, french fries.  Score range: PCA | Age, time since diagnosis, body-mass index, energy intake, smoking, physical activity, diet missing in 1986, 1990, 1994, 1998, age at menarche, oral contraceptive use, birth index, menopausal status, use of postmenopausal hormone therapy, age at menopause; tamoxifen, chemotherapy, stage at diagnosis. | Prediagnosis:  *Overall mortality* **Prudent:** RR non-significant (data not shown);  **Western:** RR 1.40 (0.93, 2.09);   *Breast cancer mortality* **Prudent:** RR non-significant (data not shown);  **Western:** RR 1.01 (0.59, 1.72);   *Non-breast cancer mortality* **Prudent:** RR non-significant (data not shown);  **Western:** RR 1.95 (1.06, 3.60);   Postdiagnosis:  *Overall mortality* **Prudent:** RR 0.78 (0.54, 1.12);  **Western:** RR 1.53 (1.03, 2.29);   *Breast cancer mortality* **Prudent:** RR 1.07 (0.66, 1.73);  **Western:** RR 1.01 (0.60, 1.70);   *Non-breast cancer mortality* **Prudent:** RR 0.54 (0.31, 0.95);  **Western:** RR 2.31 (1.23, 4.32);   Fifth vs. first quintile |
| Kwan et al. (2009) | USA  LACE | Overall mortality, Breast cancer mortality, Non-breast cancer mortality, Cancer recurrence | 1,901  5.93 | W  Prudent pattern: Q1: 58.6±11.5; Q4: 58.4±10.5 Western pattern: Q1: 59.3±10.3; Q4: 57.1±11.1 | Breast cancer | FFQ (diet of the last 12 months) at baseline (participants entered the cohort over an approximately 3-year period since diagnosis)  Postdiagnosis  Assessment of recurrence: semi-annual or annual health status update questionnaire | *Principal component analysis* **Prudent pattern:** ↑ cruciferous vegetables, other vegetables, tomatoes, dark yellow vegetables, fruits, legumes, onions, leafy vegetables, fish, soups, whole grains, poultry, not fried, salad dressings (all types), rice, grains and plain pasta, fruit juice, low-fat dairy, nuts, potatoes (not fried), cold cereals.  **Western pattern:** ↑ red meat, processed meats, creamy soups/sauces, butter, mayonnaise, Italian foods, fried potatoes, high-fat dairy, fried chicken, snacks, refined grains, pasta or potato salads, Mexican foods, sweets, high-energy drinks, eggs, organ meats.  Score range: PCA | Age at diagnosis, total energy intake, race, body mass index at enrollment, total physical activity, smoking, menopausal status at diagnosis, weight change from before diagnosis to baseline, stage of cancer, hormone receptor status, treatment. | *Overall mortality* **Prudent:** HR 0.57 (0.36, 0.90);  **Western:** HR 1.53 (0.93, 2.54);   B*reast cancer mortality* **Prudent:** HR 0.79 (0.43, 1.43);  **Western:** HR 1.20 (0.62, 2.32);   *Non-breast cancer mortality* **Prudent:** HR 0.35 (0.17, 0.73);  **Western:** HR 2.15 (0.97, 4.77);   *Cancer recurrence* **Prudent:** HR 0.95 (0.63, 1.43);  **Western:** HR 0.98 (0.62, 1.54);   Fourth vs. first quartile |
| Lee et al. (2020) | USA  NHS and HPFS | Overall mortality, Multiple myeloma cancer mortality | 423  3.5 | M/W  NHS: 69.6±7.8; HPFS: 72.3± 9.2 | Multiple myeloma | FFQ prior to diagnosis  Prediagnosis | **AHEI-2010** 1. ↑ vegetables (except for potatoes); 2. ↑ fruit; 3. ↑ whole grains; 4. ↓ sugar-sweetened beverages and fruit juice; 5. ↑ nuts and legumes; 6. ↓ red/processed meat; 7. ↓ trans fat; 8. ↑ LCFA n-3 (EPA + DHA); 9. ↑ PUFA; 10. ↓ sodium; 11. ↔ alcohol. For each 0-10 points.  Score range: 0-110  **aMED** 1. ↑ total vegetables (excluding potatoes); 2. ↑ total fruit; 3. ↑ nuts; 4. ↑ legumes; 5. ↑ fish; 6. ↑ whole grains; 7. ↑ MUFA:SFA ratio; 8. ↔ alcohol; 9. ↓ red and processed meat. 1 point for intake was greater/less than the cohort-specific median.   Score range: 0-9  **DASH** 1. ↑ total vegetables (excluding potatoes); 2. ↑ total fruit; 3. ↑ nuts, seeds, and legumes; 4. ↑ low-fat dairy; 5. ↑ whole grains; 6. ↓ sodium; 7. ↓ sugar-sweetened beverages and fruit juices; 8. ↓ red and processed meat. Each 1-8 points.  Score range: 8-40  *Principal component analysis* **Prudent pattern:** ↑ vegetables, fruits, legumes, whole grains, fish.  **Western pattern:** ↑ red/processed meats, high-fat dairy products, refined grains, sweets/desserts.  Score range: PCA | Age at diagnosis in years, prediagnosis energy intake, prediagnosis body mass index, time between food frequency questionnaire return date and multiple myeloma diagnosis, year of diagnosis, comorbidity score. The pooled analysis was additionally stratified by sex. | *Overall mortality* **AHEI-2010:** HR 0.60 (0.46, 0.79);  **aMED:** HR 0.65 (0.48, 0.87);  **DASH:** HR 0.73 (0.55, 0.96);  **Prudent:** HR 0.58 (0.42, 0.78);  **Western:** HR 1.60 (1.16, 2.20);   *Multiple myeloma* *cancer mortality* **AHEI-2010:** HR 0.55 (0.41, 0.74); **aMED:** HR 0.60 (0.44, 0.83); **DASH:** HR 0.71 (0.52, 0.96); **Prudent:** HR 0.55 (0.39, 0.77); **Western:** HR 1.80 (1.28, 2.54);  Third vs. first tertile  (pooled analyses of women and men) |
| Lei et al. (2021) | China  HKNKBCSS | Overall mortality, Breast cancer mortality, Cancer recurrence | 1,226  54.1 months | W  52.3±9.0 | Breast cancer | FFQ (diet over the previous year) assessed at 18-month follow-up after diagnosis  Postdiagnosis  Assessment of recurrence: medical records and asked during follow-ups through face-to-face interview | *Principal component analysis* **Western dietary pattern:** ↑ refined grain, red meat, fish and seafood, oil, cakes and snacks, processed meat, eggs; ↓ whole grain, leaf vegetables.  **Healthy dietary pattern:** ↑ leafy vegetables, non-leafy vegetables, fruits, potatoes, legumes.  Score range: PCA | Age at 18-month follow-up, total number of comorbidities at 18-month follow-up, stage at diagnosis, ER status, PR status, HER2 status, histology, chemotherapy, radiotherapy, adjuvant hormonal therapy usage at 18-month follow-up, menopausal status at 18-months follow-up, BMI at 18-month follow-up, level of physical activity at 18-month follow-up, total energy intake at 18-month follow-up. | *Overall mortality* **Western:** HR 0.79 (0.41, 1.52);  **Healthy:** HR 1.45 (0.82, 2.56);   *Breast cancer mortality* **Western:** HR 0.90 (0.45, 1.77);  **Healthy:** HR 1.37 (0.76, 2.49);   *Cancer recurrence* **Western:** HR 1.03 (0.61, 1.75);  **Healthy:** HR 1.01 (0.64, 1.59);   Third vs. first tertile |
| Luo et al. (2020) | China  GLCC | Overall mortality, Hepato-cellular carcinoma mortality | 887  797 days | M/W  51.9±12.0 | Hepatocellular carcinoma | FFQ (diet of the last year before diagnosis)  Prediagnosis | **CHEI** 1. ↑ total vegetables; 2. ↑ dark vegetables; 3. ↑ total grains; 4. ↑ whole grains and mixed beans; 5. ↑ tubers; 6. ↑ dairy; 7. ↑ soybeans; 8. ↑ fish and seafood; 9. ↑ poultry; 10. ↑ eggs; 11. ↑ seeds and nuts; 12. ↓ red meat; 13. ↓ added sugar; 14. ↓ alcohol; (each 0-5 points) 15. ↑ total fruits; 16. ↓ sodium; 17. ↓ cooking oils (each 0-10 points).  Score range: 0-100  **HEI-2015** 1. ↑ total fruits; 2. ↑ whole fruits; 3. ↑ total vegetables; 4. ↑ greens and beans; total protein; 6. ↑ seafood and plant protein; (each 0-5 points) 7. ↑ whole grain; 8. ↑ dairy; 9. ↑ (PUFA + MUFA)/SFA ratio; 10. ↓ refined grains; 11. ↓ sodium; 12. ↓ added sugar; 13. ↓ saturated fat (each 0-10 points).  Score range: 0-100 | Age at diagnosis, sex, energy intake, body mass index, smoking status, education level, alcohol drinking status, C‐reactive protein level, alpha‐fetoprotein level, Child–Pugh class, TNM stage, cancer treatment. | *Overall mortality* **CHEI:** HR 0.75 (0.58, 0.98);  **HEI-2015:** HR 0.86 (0.67, 1.11);   *hepatocellular carcinoma mortality* **CHEI:** HR 0.74 (0.56, 0.98);  **HEI-2015:** HR 0.93 (0.71, 1.21);  Third vs. first tertile |
| McCullough et al. (2016) | USA  ACS’s CPS-II | Overall mortality, Breast cancer mortality | Pre-diagnosis:  4,452  9.8±4.9  Post-diagnosis: 2,152  9.9±3.3 | W  70.7±7.2 | Breast cancer | FFQ Prediagnosis: at baseline (median: 7.9 years between FFQ and diagnosis) Postdiagnosis: returned at least 1 year after diagnosis (median: 3.1 years between diagnosis and FFQ)  Prediagnosis; Postdiagnosis | **ACS** 1. ↑ fruits/vegetables (servings/d, variety); 2. ↑ whole grains:refined grains ratio; 3. ↓ red/processed meat (for each 0-3 points).  Score range: 0-9 points | Age at diagnosis, diagnosis year, tumor stage at diagnosis, tumor grade at diagnosis, estrogen receptor status, progesterone receptor status, initial treatment, and the following assessed at the time of FFQ completion: BMI, smoking status, physical activity and energy intake. | Prediagnosis:  *Overall mortality* **ACS:** HR 1.00 (0.84, 1.18);   *Breast cancer mortality* **ACS:** HR 1.06 (0.79, 1.42);   Postdiagnosis:  *Overall mortality* **ACS:** HR 0.93 (0.73, 1.18);   *Breast cancer mortality* **ACS:** HR 1.44 (0.90, 2.30);   Third vs. first tertile |
| Meyerhardt et al. (2007) | USA  CALGB 89803 | Overall mortality, Cancer recurrence | 1,009  5.3 | M/W  Prudent pattern: Q1: 21-83 Q4: 28-81 Western pattern: Q1: 33-74 Q4: 28-83 | Colon cancer (stage III) | FFQ (diet over the past 3 months) during therapy and 6 months after therapy  Postdiagnosis  Assessment of recurrence: confirmed by biopsy within the study protocol (clinical trial) | *Principal component analysis* **Prudent pattern:** ↑ vegetables, leafy vegetables, yellow vegetables, cruciferous vegetables, legumes, fruit, light salad dressing, tomatoes, garlic, fish, poultry, fruit juice, whole grains, low-fat mayonnaise, regular salad dressing, wine, potatoes, cream soup or chowder, tea; ↓ french fries, sugar beverages.  **Western pattern:** ↑ high-fat dairy, low-fat dairy, refined grains, condiments, red meat, sweets and desserts, margarine, processed meat, potatoes, tomatoes, regular mayonnaise, butter, french fries, eggs, snacks, nuts, coffee, sugar beverages, beer, cream soup or chowder, pizza, regular salad dressing.  Score range: PCA | Sex, age, depth of invasion through bowel wall, number of positive lymph nodes, presence of clinical perforation at time of surgery, presence of bowel obstruction at time of surgery, baseline performance status, treatment group, weight change between first and second questionnaire, time-varying body mass index, time-varying physical activity level, time-varying total calories. | *Overall mortality* **Prudent:** HR 1.32 (0.86, 2.04);  **Western:** HR 2.32 (1.36, 3.96);   *Cancer recurrence* **Prudent:** HR 1.13 (0.77, 1.67);  **Western:** HR 2.85 (1.75, 4.63);   Fifth vs. first quintile |
| Ollberding et al. (2013) | USA  population-based, case-control study from which the cases were obtained by the Nebraska Lymphoma Study Group | Overall mortality | 301  8.2 | M/W  20-75 (age at study entry) | Non-Hodgkin lymphoma | FFQ during the year preceding diagnosis  Prediagnosis | *Principal component analysis* **“Fruit, vegetable and starch” dietary pattern:** ↑ other vegetables, tomatoes and juice, cruciferous vegetables, green leafy vegetables, fruit and fruit juice, legumes and soya, vegetable soup, fish, mayonnaise or creamy salad dressing, cereals and starches, potatoes, other soups, low-fat dairy products.  Score range: PCA | Age, sex, education, smoking status, total energy intake. | *Overall mortality* **Fruit, vegetable and starch:** HR 1.0 (0.5, 1.8)  Third vs. first tertile |
| Park et al. (2022) | USA  MEC | Overall mortality, Cancer-specific mortality | 6,370  10.7 | M/W  ♂ 68.1±7.8; ♀ 65.4±8.6 | All cancer types | FFQ  at baseline and 10-year follow-up surveys (only 10-year-follow-up was used for measurement of diet quality)  Postdiagnosis | **HEI-2015** 1. ↑ total fruits; 2. ↑ whole fruits; 3. ↑ total vegetables; 4. ↑ greens and beans; 5. total protein; 6. ↑ seafood and plant protein; (each 0-5 points) 7. ↑ whole grain; 8. ↑ dairy; 9. ↑ (PUFA + MUFA)/SFAs ratio; 10. ↓ refined grains; 11. ↓ sodium; 12. ↓ added sugar; 13. ↓ saturated fat (each 0-10 points, calculated per 1000 kcal or percentage of energy).  Score range: 0-100 **AHEI-2010** 1. ↑ vegetables (excluding potatoes); 2. ↑ whole fruit; 3. ↑ whole grains; 4. ↑ nuts and legumes; 5. ↑ LCFA n-3; 6. ↑ PUFA; 7. ↓ sugar-sweetened beverages; 8. ↓ red/processed meat; 9. ↓ sodium; 10. ↓ trans fat; 11. ↔ alcohol. For each food group 0 to 10 points.  Score range: 0-110  **aMED** 1. ↑ total vegetables (excluding potatoes); 2. ↑ total fruit; 3. ↑ nuts; 4. ↑ legumes; 5. ↑ fish; 6. ↑ whole grains; 7. ↑ MUFA/SFA ratio; 8. ↔ alcohol; 9. ↓ red and processed meat. 1 point for intake was greater/less than the cohort specific median.   Score range: 0-9  **DASH** 1. ↑ total vegetables (excluding potatoes and legumes); 2. ↑ nuts and legumes; 3. ↑ total fruit; 4. ↑ whole grains; 5. ↑ low-fat dairy; 6. ↓ sodium; 7. ↓ red and processed meat; 8. ↓ sweetened beverages (for each 1-5 points).  Score range: 8-40 points | Sex, age, race/ethnicity, education, body mass index, physical activity, marital status, comorbidity, total energy intake, menopausal hormone therapy for women only in the smoking model that included smoking status, average number of cigarettes, squared average number of cigarettes, number of years smoked (time-dependent), number of years since quitting (time-dependent), interactions between ethnicity and smoking status, average number of cigarettes, squared average number of cigarettes, number of years smoked, tumor stage, radiation and chemotherapy treatment, and years between diagnosis and 10-year follow-up. For HEI-2015 and DASH, further adjusted for alcohol intake. | *Overall mortality* **HEI-2015:** HR 0.74 (0.67, 0.82);  **AHEI-2010:** HR 0.82 (0.74, 0.92);  **aMED:** HR 0.74 (0.66, 0.84);  **DASH:** HR 0.82 (0.74, 0.91);   *Cancer-specific mortality* **HEI-2015:** HR 0.84 (0.71, 1.00);  **AHEI-2010:** HR 0.85 (0.71, 1.00);  **aMED:** HR 0.71 (0.59, 0.85);  **DASH:** HR 0.84 (0.71, 1.00);  Fourth vs. first Quartile |
| Pelser et al. (2014) | USA  NIH-AARP Diet and Healthy Study cohort | Overall mortality, Cancer-specific mortality | 5,727  (Colon cancer: 4,213;  Rectal cancer: 1,514)  5 | M/W  Colon cancer: Q1: 68.4; Q5: 70.5; Rectal cancer: Q1: 68.4; Q5: 69.8; (mean age) | Colon cancer, Rectal cancer | FFQ (for the previous 12 months) at baseline  Prediagnosis | **HEI-2005** 1. ↑ total fruit, whole fruit, total vegetables, dark-green vegetables and orange vegetables and legumes, total grains, whole grains (each 0-5 points); 2. ↑ milk, meats and beans (each 0-10 points); 3. ↓ oils, saturated fat, sodium (each 0-10 points); 4. ↓ calories from solid fat, alcohol, added sugar (0-20 points);  Score range: 0-100 | Age as time metric, lag time, sex, education, family history of colon cancer, cancer stage, first course of treatment (surgery, radiation, chemotherapy), BMI, physical activity, alcohol, smoking history. | Colon cancer: *Overall mortality* **HEI-2005:** HR 0.95 (0.78, 1.16);  *Cancer-specific mortality* **HEI-2005:** HR 0.99 (0.77, 1.27);  Rectal Cancer *Overall mortality* **HEI-2005:** HR 0.60 (0.42, 0.86);  *Cancer-specific mortality* HEI-2005: 0.64 (0.41, 0.99);  Fifth vs. first quintile |
| Ratjen et al. (2017) | Germany  PopGen biobank | Overall mortality | 1,404  7 | M/W  57–66 | Colorectal cancer | FFQ (diet of the previous 12 months) at a median time of 6 years after diagnosis  Postdiagnosis | **MMDS** (based on MED) 1. ↑ vegetables; 2. ↑ fruit and nuts; 3. ↑ legumes; 4. ↑ cereals; 5. ↑ fish; 6. ↓ meat and poultry products; 7. ↓ dairy products; 8. ↓ MUFA and PUFA:SFA ratio; 9. ↔ alcohol. 1 point for intake was greater/less than the cohort specific median.   Score range: 0-9 points  **HNFI** 1. ↑ cabbage; 2. ↑ root vegetables; 3. ↑ whole-grain bread (instead of rye bread); 4. ↑ oatmeal; 5. ↑ apples and pears; 6. ↑ fish and shellfish. 1 point for intake was greater/less than the cohort specific median.   Score range: 0-6 points | Sex, age at diet assessment, BMI, physical activity, survival time from colorectal cancer diagnosis until diet assessment, tumor location, occurrence of metastases, occurrence of other cancer, chemotherapy, smoking status, total energy intake, time x age, time x BMI, time x metastases | *Overall mortality* **MMDS:** HR 0.48 (0.32, 0.74);  **HNFI:** HR 0.63 (0.39, 1.04);  Fourth vs. first quartile |
| Ratjen et al. (2021) | Germany  PopGen biobank | Overall mortality | 1,404  7 | M/W  57–66 | Colorectal cancer | FFQ (diet during the previous 12 months) median of 6 years after diagnosis  Postdiagnosis | **PDI:** 1. ↑ whole grains, fruits, vegetables, nuts, legumes, vegetable oils, tea, coffee;  2. ↑ fruit juices, refined grains, potatoes, sugar-sweetened beverages, sweets and desserts;  3. ↓ dairy, animal fat, egg, meat, fish or seafood, miscellaneous animal-based foods (Pizza, cream soups like chowders, mayonnaise, and sandwich spreads).  **hPDI:**  1 ↑ whole grains, fruits, vegetables, nuts, legumes, vegetable oils, tea, coffee;  2. ↓ fruit juices, refined grains, potatoes, sugar-sweetened beverages, sweets and desserts;  3. ↓ dairy, animal fat, egg, meat, fish or seafood, miscellaneous animal-based foods.  **uPDI:**  1. ↓ whole grains, fruits, vegetables, nuts, legumes, vegetable oils, tea, coffee;  2. ↑ fruit juices, refined grains, potatoes, sugar-sweetened beverages, sweets and desserts;  3. ↑ dairy, animal fat, egg, meat, fish or seafood, miscellaneous animal-based foods.  total serving size consumption for each food group was broken into sex-specific quintiles, and each quintile was given a score between 1 and 5.   Score range: 18-90 points | Sex, age at diet assessment, BMI, physical activity, survival time from colorectal cancer diagnosis until diet assessment, tumor location, metastases, other cancer, type of therapy, smoking status, alcohol intake, total energy intake, time × age, time × BMI, time × metastases. | *Overall mortality* **PDI:** HR 0.46 (0.29, 0.75);  **hPDI:** HR 0.76 (0.51, 1.14);  **uPDI:** HR 1.29 (0.84, 1.98);   Fourth vs. First quintile |
| Sharma et al. (2018) | Canada  Newfoundland and Labrador Familial Colorectal Cancer cohort | Overall mortality | 532  6.27 | M/W  60.42±9.02 | Colorectal cancer | FFQ retrospectively a year before diagnosis  Prediagnosis | **aMED** 1. ↑ total vegetables (excluding potatoes); 2. ↑ legumes; 3. ↑ total fruit; 4. ↑ nuts; 5. ↑ whole grains; 6. ↓ red and processed meat; 7. ↑ fish; 8. ↑ MUFA:SFA ratio; 9. ↔ alcohol.  1 point for intake was greater/less than the cohort specific median.   Score range: 0-9  **RFS** 1. vegetables (22 items); 2. fruit (13 items); 3. protein (5 items); 4. grains (6 items); 5. dairy (skim milk).  1 point for each item consumed at least weekly.   Score range: 0-47  *Principal component analysis* **Processed meat pattern:** ↑ red meat, cured/processed red meat, cured/processed meat, fish, processed fish.  **Prudent vegetable pattern:** ↑ cruciferous vegetables, other fruits, other greens, tomato sauce, other vegetables.  **High sugar pattern:** ↑ desserts and sweets, pies, tarts.   Score range: PCA | Energy, stage of cancer, sex, age, marital status, tumour location, screening history, intake of alcohol, radiation and chemo therapy status, Microsatellite instability status wherever applicable. | *Overall mortality* **aMED:** HR 1.62 (1.04, 2.56);  **RFS:** HR 1.54 (0.92, 2.56);   Lowest vs. highest quartile  **Processed meat:** HR 1.53 (0.85, 2.27);  **Prudent vegetable:** HR 1.03 (0.61, 1.75);  **High sugar:** HR 1.27 (0.72, 2.23);  Highest vs. lowest quartile |
| Song et al. (2021) | USA  NHS and HPFS | Overall mortality, Colorectal cancer mortality | 1,491  7.92 | M/W  Q1: 69.1±8.9; Q4: 69.0±8.5 | Colorectal cancer | FFQ pre-diagnosis: last questionnaire collected before diagnosis or if missing, the most recently completed questionnaire from at most the two previous assessments was used; post-diagnosis: six months to four years after diagnosis  Prediagnosis; Postdiagnosis | **WCRF/AICR 2018** (only diet)) 1. ↑ fruits and vegetables; 2. ↑ dietary fiber; 3. ↑ whole grains, nuts and legumes; 4. ↓ refined grains and processed foods high in fat and sugar; 5. ↓ red and processed meat; 6. ↓ sugar-sweetened beverages; 7. ↓ alcohol. For each 0, 0.5 or 1 point, for components 5 and 6, each of their respective sub-components was assigned a score of 0, 0.5, or 1; the sub-components were then averaged to construct the component score.  Score range: 0 to 7 points | Prediagnosis: Age at diagnosis, cohort, tumor grade of differentiation, tumor subsite, year of diagnosis, regular sigmoidoscopy/colonoscopy screening, pre-diagnostic total energy intake, pre-diagnostic BMI, pre-diagnostic physical activity, pre-diagnostic regular use of aspirin, pre-diagnostic smoking.  Stratified by age at diagnosis and tumor stage.  Postdiagnosis: Age at diagnosis, cohort, tumor grade of differentiation, tumor subsite, year of diagnosis, regular sigmoidoscopy/colonoscopy screening, post-diagnostic total energy intake, post-diagnostic BMI, post-diagnostic physical activity, post-diagnostic regular use of aspirin, post-diagnostic smoking, pre-diagnostic WCRF/AICR diet score.  Stratified by age at diagnosis and tumor stage. | Prediagnostic WCRF/AICR (diet score only) *Overall mortality* **WCRF/AICR-2018:** HR 0.90 (0.71, 1.14);   *Colorectal cancer mortality* **WCRF/AICR-2018:** HR 0.78 (0.50, 1.22);   Postdiagnostic WCRF/AICR (diet score only)  *Overall mortality* **WCRF/AICR-2018:** HR 0.97 (0.73, 1.28);   *Colorectal cancer mortality* **WCRF/AICR-2018:** HR 1.09 (0.65, 1.81);   Fourth vs. first quartile |
| Sun et al. (2018) | USA  WHI | Overall mortality, Breast cancer mortality, Non-breast cancer mortality | 2,295  12 | W  HEI-2010 score change: Decrease: 66.4±6.9; No change or stable: 66.1±6.8; Increase: 65.0±6.9 | Breast cancer (postmeno-pausal) | FFQ  average time from prediagnosis FFQ to diagnosis: 1.5 years; average time from diagnosis to postdiagnosis FFQ: 1.8 years  Prediagnosis; Postdiagnosis | **HEI-2010** 1. ↑ total fruit, whole fruit, total vegetables, green vegetables and beans, total protein foods, seafood and plant proteins (each 0-5 points); 2. ↑ whole grains, dairy, PUFA and MUFA:SFA ratio (each 0-10 points); 3. ↓ refined grains, sodium (each 0-10 points);  4. ↓ empty calories (0-20 points).  Score range: 0-100 points | Age at diagnosis, prediagnosis total energy intake, race or ethnicity, education, income, breast cancer stage, estrogen receptor status, progesterone receptor status, prediagnosis smoking, prediagnosis physical activity, observational study vs dietary modification clinical trial intervention arm vs dietary modification clinical trial comparison arm, use of postmenopausal hormone therapy, prediagnosis alcohol intake, prediagnosis body mass index. | Prediagnosis: *Overall mortality* **HEI-2010:** HR 0.90 (0.72, 1.12);  *Breast cancer mortality* **HEI-2010:** HR 1.12 (0.76, 1.64);   *Non-breast cancer mortality* **HEI-2010:** HR 0.83 (0.63, 1.08);   Postdiagnosis: *Overall mortality* **HEI-2010:** HR 0.82 (0.66, 1.02);   *Breast cancer mortality* **HEI-2010:** HR 0.97 (0.66, 1.43);   *Non-breast cancer mortality* **HEI-2010:** HR 0.72 (0.55, 0.94);   Fourth vs. first quartile |
| Thomson et al. (2014) | USA  WHI | Overall mortality, Cancer-specific mortality | 636  1995-2012 | W  62.9 (mean age) | Ovarian cancer (postmeno-pausal) | FFQ at least 12 months before diagnosis  Prediagnosis | **HEI-2005:**  1. ↑ total fruit, whole fruit (not juice), total vegetables, dark green and orange vegetables and legumes, total grains, whole grains (each 0-5 points); 2. ↑ milk, meat and beans, oils (each 0-10 points); 3. ↓ oils, saturated fat, sodium (each 0-10 points); 4. ↓ calories from solid fats, alcoholic beverages, added sugars (0-20 points).  Score range: 0-100 | Age at diagnosis, stage at diagnosis, race/ethnicity, diabetes, physical activity, total energy intake, waist circumference, family history of ovarian cancer, clinical trial arms. | *Overall mortality* **HEI-2005:** HR 0.73 (0.55, 0.97);  *Ovarian cancer mortality* **HEI-2005:** HR 0.75 (0.55, 1.01);  Third vs. first tertile |
| Van Blarigan et al. (2020) | USA  CALGB/SWOG 80405 | Overall mortality | 1,284  73 months | M/W  59 (median) | Colorectal cancer (metastatic) | FFQ (diet of past 3 months) within 4 weeks after treatment initiation  Prediagnosis | **AHEI** 1. ↑ vegetables (excluding potatoes); 2. ↑ fruit; 3. ↑ whole grains; 4. ↑ nuts and legumes; 5. ↑ LCFA n-3; 6. ↑ PUFA; 7. ↓ sugar-sweetened beverages and juice; 8. ↓ red/processed meat; 9. ↓ sodium; 10. ↓ trans fat; 11. ↔ alcohol. For each food group 0 to 10 points.  Score range: 0-110  **aMED** 1. ↑ total vegetables (excluding potatoes); 2. ↑ fruit; 3. ↑ nuts; 4. ↑ legumes; 5. ↑ fish; 6. ↑ whole grains; 7. ↑ MUFA/SFA ratio; 8. ↔ alcohol; 9. ↓ red and processed meat. 1 point for intake was greater/less than the cohort specific median.   Score range: 0-9  **DASH** 1. ↑ total vegetables (excluding potatoes and legumes); 2. ↑ nuts and legumes; 3. ↑ total fruit; 4. ↑ whole grains; 5. ↑ low-fat dairy; 6. ↓ sodium; 7. ↓ red and processed meat; 8. ↓ sweetened beverages; 9. ↓ sweets and desserts (for each 1-5 points).  Score range: 8-45 points  *Principal component analysis* **Western dietary pattern:** ↑ dairy, refined grains, condiments, red meat, sweets and desserts.  **Prudent dietary pattern:** ↑ vegetables, legumes, fruit.  Score range: PCA | Age, sex, kcal/d, race/ethnicity; performance status; protocol chemotherapy; primary tumor unresected; diabetes; treatment arm; KRAS status; tumor sidedness; weight change in previous 6 months; body mass index; physical activity. | *Overall mortality* **AHEI:** HR 0.88 (0.73, 1.08);  **AMED:** HR 0.83 (0.67, 1.04);  **DASH:** HR 0.91 (0.75, 1.12);   **Western:** HR 0.83 (0.66, 1.04);  **Prudent:** HR 0.85 (0.65, 1.10);   Fifth vs first quantile |
| Van Zutphen et al. (2021) | Netherlands  COLON and EnCoRe | Overall mortality, Cancer recurrence | 1,425  2.6 (for cancer recurrence); 4.4 (for mortality) | M/W  66 | Colorectal cancer  (stage I–III) | COLON: FFQ (refers to the previous month) 6 months after diagnosis  EnCoRe: 7-day dietary record 6 months after treatment  Postdiagnosis  Assessment of recurrence: medical records were checked by trained registrars from the Dutch Cancer Registry. | **WCRF/AICR sub-score for diet** 1. ↑ fruits and vegetables; 2. ↑ dietary fiber (each 0-0.5 points); 3. ↓ processed foods; 4. ↓ red and processed meat; 5. ↓ sugary drinks grains (each 0-1 points);   Score range: 0 to 4 points  **ACS sub-score for diet** 1. ↑ fruits/vegetables (servings/d, variety); 2. ↑ whole grains:refined grains ratio; 3. ↓ red/processed meat (for each 0-3 points).  Score range: 0-9 points  **DHDI-2015** 1. ↑ fruit, vegetables, legumes, nuts, fish, tea, ratio liquid fats to solid fats (each 0-10 points); 2. ↑ whole grains, ratio of wholegrains to refined grains (each 0-5 points); 3. diary (0-10 points); 3. ↓ red meat, processed meat, sugary drinks (each 0-10 points).  Score range: 0-120 points | Age at diagnosis, stage of disease, sex, adjuvant chemotherapy, education, smoking, and cohort. All components were mutually adjusted for each other. | *Overall mortality* **WCRF/AICR:** HR 1.07 (0.67, 1.70); **ACS:** HR 0.85 (0.45, 1.60); **Dutch Healthy Diet Index:** HR 0.74 (0.47, 1.16);  *Cancer recurrence* **WCRF/AICR:** HR 1.16 (0.74, 1.82); **ACS:** HR 1.32 (0.74, 2.37); **Dutch Healthy Diet Index:** HR 1.16 (0.73, 1.83);  ***WCRF/AICR and ACS:*** *Third vs. first tertile* ***Dutch Healthy Diet Index:*** *Fourth vs. first quartile* |
| Vrieling et al. (2013) | Germany  MARIE study | Overall mortality, Breast cancer mortality, Non-breast cancer mortality, Cancer recurrence | 2,522  5.5 | W  50-74 | Breast cancer (invasive, postmeno-pausal) | FFQ refers to the year prior to diagnosis  Prediagnosis  Assessment of recurrence: medical records were checked or treating physicians were contacted | *Principal component analysis* **Healthy pattern:** ↑ vegetables, fruits, vegetable oil, sauces/condiments, soups/bouillons intake.  **Unhealthy pattern:** ↑ red meat, processed meat, deep-frying fat intake.  Score range: PCA | Tumour size, nodal status, metastases, tumour grade, ERPR status, radiotherapy, HRT use at diagnosis, mode of detection, total energy intake.  Non-breast cancer mortality: additionally adjusted for cardiovascular disease (because of missing covariate values, 66 observations were not included in model 2). Cancer recurrence: because of missing covariate values, 49 observations were not included in model 2.  Stratified by age at diagnosis and study centre. | *Overall mortality* **Healthy:** HR 0.87 (0.61, 1.23); **Unhealthy:** HR 1.34 (0.93, 1.94);  *Breast cancer mortality* **Healthy:** HR 0.89 (0.59, 1.35); **Unhealthy:** HR 0.99 (0.64, 1.52);  *Non-breast cancer mortality*  **Healthy:** HR 0.81 (0.40, 1.61); **Unhealthy:** HR 3.69 (1.66, 8.17);  *Cancer recurrence* **Healthy:** HR 0.71 (0.48, 1.06); **Unhealthy:** HR 0.91 (0.61, 1.36);  Fourth vs. first quartile |
| Wang et al. (2020) | China  SBCSS | Overall mortality, Breast cancer mortality | 3,450  5.0 | W  Q1: 60.7±10.4; Q4:  57.5± 8.9; (age at study entry) | Breast cancer | FFQ 5-year postdiagnosis  Postdiagnosis | **CHFP-2007** 1. ↓ salt; 2. ↓ fats and oil; 3. ↑ dairy products; 4. ↑ beans; 5. ↑ vegetables; 6. ↑ fruits; 7. ↑ grains (each 0-5 points); 8. ↓ meat and poultry (0-4 points); 9. ↑ fish; 10. ↓ eggs (each 0-3 points).  Score range: 0-45 points  **CHFP-2016** 1. ↓ salt; 2. ↓ fats and oil; 3. ↑ dairy products; 4. ↑ beans; 5. ↑ vegetables; 6. ↑ fruits; 7. ↑ grains (each 0-5 points); 8. ↓ meat and poultry (0-4 points); 9. ↑ fish; 10. ↓ eggs (each 0-3 points). With different recommended intake amounts for beans, meat and poultry, fish, vegetables, fruits, grains.  Score range: 0-45 points  **modified HEI-2015** 1. ↑ total fruits; 2. ↑ whole fruits; 3. ↑ total vegetables; 4. ↑ greens and beans; 5. ↑ total protein; 6. ↑ seafood and plant protein; 7. ↑ dairy; (each 0-5 points) 8. ↓ refined grains; 9. ↓ added sugars; 10. ↑ fatty acids (MUFA and PUFA/SFA); 11. ↓ sodium; 12. ↓ saturated fats (each 0-10 points) (whole grain excluded, due to low consummation in the Shanghai area).  Score range: 0-100 points  **modified DASH** 1. ↑ vegetables; 2. ↑ fruits; 3. ↑ dairy products; 4. ↓ poultry, fish and eggs; 5. ↑ nuts (nuts, beans, legumes); 6. ↓ fats and oil; 7. ↓ sodium (each 0-10 points).  Score range: 0-70 points | Age at 60-month survey, intervals between diagnosis and 60-month survey, total energy intake, income, education, marriage, menopausal status at diagnosis, BMI at 60-month survey, physical activity at 60-month survey, ER, PR, HER2, TNM stages, comorbidity, chemotherapy, radiotherapy, immunotherapy. | *Overall mortality* **CHFP-2007:** HR 0.66 (0.48, 0.89); **CHFP-2016:** HR 0.75 (0.55, 1.01); **DASH:** HR 0.66 (0.49, 0.91); **HEI-2015:** HR 0.79 (0.57, 1.10);  *Breast cancer mortality* **CHFP-2007:** HR 0.58 (0.40, 0.84); **CHFP-2016:** HR 0.70 (0.48, 1.01); **DASH:** HR 0.63 (0.44, 0.92); **HEI-2015:** HR 0.86 (0.58, 1.27);  Fourth vs. first quartile |
| Wen et al. (2022) | China  OOPS | Overall mortality | 703  37.57 months | W  Alive:53.32±9.11; Deceased: 54.98±10.74 | Ovarian cancer | FFQ during the year prior to diagnosis  Prediagnosis | *Principal component analysis* **Healthy pattern:** ↑ fruit, egg, vegetable, legumes and legume products, whole grain, fish, tubers, ginger and garlic.  **Ethnic pattern:** ↑ sesame paste, fruit or vegetable juice, coffee, alcohol and alcoholic beverages.  **Animal foods pattern:** ↑ animal organs, animal blood, processed meat products.  **Sweet pattern:** ↑ sugar-containing beverages, ice cream, candy and cake.  Score range: PCA | Age at diagnosis, total energy intake, body mass index, diet change, comorbidities, education, FIGO stage, histological type, histopathologic grade, menopausal status, parity, oral contraceptives, physical activity, residual lesions, smoke status, other dietary patterns. | *Overall mortality* **Healthy:** HR 0.54 (0.30, 0.98);  **Ethnic:** HR 1.19 (0.75, 1.91);  **Animal foods:** HR 1.90 (1.14, 3.17);  **Sweet:** HR 0.70 (0.44, 1.12);  Third vs. first tertile |
| Yang et al. (2015) | USA  PHS I or II | Overall mortality, Prostate cancer mortality | 926  9.9 | M  40-84 (at study entry) | Prostate cancer (nonmetastatic) | FFQ (diet over the previous year) 5.1 years (median) after diagnosis  Postdiagnosis | *Principal component analysis* **Prudent pattern:** ↑ legumes, vegetables, fruits, whole grains, garlic, soy products, fish, oil and vinegar dressing.  **Western pattern:** ↑ processed and red meats, eggs, potatoes, high-fat dairy products, butter, refined grains, snacks, sweets, desserts.  Score range: PCA | Age at diagnosis, total energy intake, BMI, smoking status, vigorous exercise, Gleason score, clinical stage, prostate-specific antigen level, time interval between diagnosis and FFQ completion, initial treatment after diagnosis (radiation, prostatectomy, others, unspecified or missing), family history of prostate cancer. | *Overall mortality* **Prudent:** 0.64 (0.44, 0.93); **Western:** RR 1.67 (1.16, 2.42);  *Prostate cancer mortality* **Prudent:** RR 0.46 (0.17, 1.24); **Western:** RR 2.53 (1.00, 6.42);  Fourth vs. first quartile |
| Zhu et al. (2013) | Canada  Patients were enrolled through the Newfound-land Familial Colorectal Cancer Registry | Overall mortality | 529  6.4 | M/W  Processed meat pattern: Q1: 61.4±8.7; Q4: 59.3±9.3 Prudent vegetable pattern: Q1: 57.4±10.3; Q4: 62.1±8.0 High-sugar pattern: Q1: 59.5±9.3; Q4: 61.7±8.6 | Colorectal cancer | FFQ one year prior to diagnosis  Prediagnosis | *Principal component analysis* **Processed meat pattern:** ↑ cured/processed meat, cured/processed red meat, red meat, fish, processed fish.  **Prudent vegetable pattern:** ↑ other greens, other fruit, other vegetables, tomato sauce.  **High-sugar pattern:** ↑ desserts and sweets, pies, tarts.  Score range: PCA | Total energy intake, sex, age at diagnosis, stage at diagnosis, marital status, family history, reported screening procedure, reported chemoradiotherapy and microsatellite instability status (where appropriate). | *Overall mortality* **Processed meat:** HR 1.53 (0.85, 2.74); **Prudent vegetable:** HR 1.03 (0.61, 1.75); **High-sugar:** HR 1.27 (0.72, 2.25);  Fourth vs. first quartile |

Abbreviations: *ACE* Adult Comorbidity Evaluation, *ACS* American Cancer Society, *AHEI* Alternative Healthy Eating Index, *aMED* Alternate Mediterranean Diet Score, *BMI* Body Mass Index, *CALGB* Cancer and Leukemia Group B, *CALGB/SWOG 80405* Cancer and Leukemia Group B/Southwest Oncology Group 80405, *CI* confidence interval, CHEI Chinese Healthy Eating Index, *CHFP* Chinese Food Pagoda, *COLON* Colorectal cancer: Longitudinal Observational study on Nutritional and lifestyle factors, *CPS* Cancer Prevention Study, *DASH* Dietary Approaches to Stop Hypertension, *DGI* Australian Dietary Guideline Index, *DHA* docosahexaenoic acid, *DHDI* Dutch Healthy Diet Index, *DQIR* Diet Quality Index-Revised, *EnCoRe* energy for life after colorectal cancer, *EPA* eicosapentaenoic acid, *ER* Estrogen Receptor, *ERPR* Estrogen/Progesterone Receptor, *FFQ* food frequency questionnaire, *FIGO* International Federation of Gynecology and Obstetrics, *GLCC* Guangdong Liver Cancer Cohort, *HEAL* Health Eating Activity and Lifestyle Study, *HEI* Healthy Eating Index, *HER2* human epidermal growth factor receptor 2, *HNFI* healthy Nordic Food Index, *hPDI* healthful plant-based diet index, *HR* hazard ration, *HKNKBCSS* Hong Kong NTEC-KWC Breast Cancer Survival Study, *HPFS* Health Professionals Follow-up Study, *HRT* hormone replacement therapy, *IWHS* Iowa Women’s Health Study, *LACE* Life After Cancer Epidemiology, *LCFA* long-chain fatty acids, *M (sex)* men, *MDS* Mediterranean Diet Score, *MEC* Multiethnic Cohort, *MED* Mediterranean Diet Score, *MMDS* Modified Mediterranean Diet Score, *MUFA* monounsaturated fatty acids, *NHANES III* Third National Health and Nutrition Examination Survey, *NHS* Nurses’ Health Study, *NIH-AARP* National Institutes of Health American Association of Retired Persons Diet and Health Study, *NSAID* nonsteroidal anti-inflammatory drugs, *OOPS* Ovarian cancer follow-up study, *OPAL* Ovarian cancer Prognosis And Lifestyle, *PCA* principal component analysis, *PDI* overall plant-based diet index, *PHS* Physicians' Health Study, *PR* Progesterone Receptor, *PUFA* polyunsaturated fatty acids, *Q* Quintile, *Ref* reference, *RFS* Recommended Food Score, *RR* relative risk, *SBCSS* Shanghai Breast Cancer Survival Study, *SFA* saturated fatty acids, *TNM* tumor nodes metastasis, *UM HN-SPORE* University of Michigan Head and Neck Specialized Program of Research Excellence, *uPDI* unhealthful plant-based diet index, *USA* United States of America, *W (sex)* women, *WCRF/AICR* World Cancer Research Fund/ American Institute for Cancer Research, *WHI* Wom en’s Health Initiative, *y* year.

Table S6: Overview of included studies in the meta-analysis analyzing diet-quality indices.

| **Author (y)** | **Cohort** | **Cancer type** | **PRE** | **POST** | **HEI 1995** | **HEI 2005** | **HEI 2010** | **HEI 2015** | **AHEI 2010** | **MED** | **aMED** | **DASH** | **ACS** | **WCRF** | **RFS** | **hPDI** | **CHFP** | **DQIR** | **DGI** | **HNFI** | **CHEI** | **DHDI** | **OM** | **CSM** | **CR** |
| --- | --- | --- | --- | --- | --- | --- | --- | --- | --- | --- | --- | --- | --- | --- | --- | --- | --- | --- | --- | --- | --- | --- | --- | --- | --- |
| Al Ramadhani et al. (2021) | OPAL | Ovarian cancer | x | x |  |  | x |  | x |  |  |  |  |  |  |  |  |  | x |  |  |  |  | x |  |
| Anyene et al. (2021) | Pathways Study | Breast cancer | x | x |  |  |  |  |  |  |  |  |  |  |  | x |  |  |  |  |  |  | x | x | x |
| Deshmukh et al. (2018) | NHANES III | All cancer types |  | x | x |  |  |  |  |  |  |  |  |  |  |  |  |  |  |  |  |  | x | x |  |
| Di Maso et al. (2020) | Italian case-control study | Breast cancer | x |  |  |  |  |  |  | x |  |  |  |  |  |  |  |  |  |  |  |  | x | x |  |
| Di Maso et al. (2021) | Italian case-control study | Prostate cancer | x |  |  |  |  |  |  | x |  |  |  |  |  |  |  |  |  |  |  |  | * | x |  |
| Ergas et al. (2021) | Pathways Study | Breast cancer | x |  |  |  |  | x |  |  | x | x | x |  |  |  |  |  |  |  |  |  | x | x | x |
| Fung et al. (2014) | NHS | Colorectal cancer |  | x |  |  |  |  | x |  | x | x |  |  |  |  |  |  |  |  |  |  | x | x |  |
| George et al. (2011) | HEAL | Breast cancer |  | x |  | x |  |  |  |  |  |  |  |  |  |  |  |  |  |  |  |  | x | x |  |
| Guinter et al. (2018) | CPS-II Nutrition Cohort | Colorectal cancer | x | x |  |  |  |  |  |  |  | x | x |  |  |  |  |  |  |  |  |  | x | x |  |
| Inoue-Choi et al. (2013) | IWHS | All cancer types |  | x |  |  |  |  |  |  |  |  |  | x |  |  |  |  |  |  |  |  | x | x |  |
| Izano et al. (2013) | NHS | Breast cancer |  | x |  |  |  |  | x |  |  | x |  |  |  |  |  |  |  |  |  |  | * | x |  |
| Jacobs et al. (2016) | MEC | Colorectal cancer | x |  |  |  | x |  | x |  | x | x |  |  |  |  |  |  |  |  |  |  | x | x |  |
| Karavasiloglou et al. (2019) | NHANES III | Breast cancer and  gynecological cancer |  | x |  |  |  |  |  | x |  |  |  |  |  |  |  |  |  |  |  |  | x |  |  |
| Kenfield et al. (2014)^79^ | HPFS | Prostate cancer |  | x |  |  |  |  |  | x | x |  |  |  |  |  |  |  |  |  |  |  | x | x |  |
| Kim et al. (2011 | NHS | Breast cancer |  | x |  |  |  |  |  |  | x |  |  |  | x |  |  | x |  |  |  |  | x | x |  |
| Lee et al. (2020) | NHS & HPFS | Multiple myeloma | x |  |  |  |  |  | x |  | x | x |  |  |  |  |  |  |  |  |  |  | x | x |  |
| Luo et al. (2020) | GLCC | Hepatocellular  carcinoma | x |  |  |  |  | x |  |  |  |  |  |  |  |  |  |  |  |  | x |  | x | x |  |
| McCullough et al. (2016) | CPS-II Nutrition Cohort | Breast cancer | x | x |  |  |  |  |  |  |  |  | x |  |  |  |  |  |  |  |  |  | x | x |  |
| Park et al. (2022) | MEC | All cancer types |  | x |  |  |  | x | x |  | x | x |  |  |  |  |  |  |  |  |  |  | x | x |  |
| Pelser et al. (2014) | NIH-AARP | Colon and rectal cancer | x |  |  | x |  |  |  |  |  |  |  |  |  |  |  |  |  |  |  |  | x | x |  |
| Ratjen et al. (2017) | PopGen biobank | Colorectal cancer |  | x |  |  |  |  |  | x |  |  |  |  |  |  |  |  |  | x |  |  | x |  |  |
| Ratjen et al. (2021) | PopGen biobank | Colorectal cancer |  | x |  |  |  |  |  |  |  |  |  |  |  | x |  |  |  |  |  |  | x |  |  |
| Sharma et al. (2018) | NLFCRC | Colorectal cancer | x |  |  |  |  |  |  |  | x |  |  |  | x |  |  |  |  |  |  |  | x |  |  |
| Song et al. (2021) | NHS & HPFS | Colorectal cancer | x | x |  |  |  |  |  |  |  |  |  | x |  |  |  |  |  |  |  |  | x | x |  |
| Sun et al. (2018) | WHI | Breast cancer | x | x |  |  | x |  |  |  |  |  |  |  |  |  |  |  |  |  |  |  | x | x |  |
| Thomson et al. (2014) | WHI | Ovarian cancer | x |  |  | x |  |  |  |  |  |  |  |  |  |  |  |  |  |  |  |  | x | x |  |
| Van Blarigan et al. (2020) | CALGB/SWOG 80405 | Colorectal cancer | x |  |  |  |  |  | x |  | x | x |  |  |  |  |  |  |  |  |  |  | x |  |  |
| Van Zutphen et al. (2021) | COLON & EnCoRe | Colorectal cancer |  | x |  |  |  |  |  |  |  |  | x | x |  |  |  |  |  |  |  | x | x |  | x |
| Wang et al. (2020) | SBCSS | Breast cancer |  | x |  |  |  | x |  |  |  | x |  |  |  |  | x** |  |  |  |  |  | x | x |  |
|  |  | **Summe:** | **16** | **19** | **1** | **3** | **3** | **4** | **7** | **5** | **9** | **9** | **4** | **3** | **2** | **2** | **1** | **1** | **1** | **1** | **1** | **1** | **28** | **23** | **3** |

*Overall mortality was calculated by combining the HR of cancer-specific mortality and non-cancer specific mortality.

**CHFP2007 and CHFP2016.

Abbreviations: *ACS* American Cancer Society, *AHEI* Alternative Healthy Eating Index, *aMED* Alternate Mediterranean Diet Score, *CALGB/SWOG 80405* Cancer and Leukemia Group B/Southwest Oncology Group 80405, CHEI Chinese Healthy Eating Index, *CHFP* Chinese Food Pagoda, *COLON* Colorectal cancer: Longitudinal Observational study on Nutritional and lifestyle factors, *CPS* Cancer Prevention Study, *CR* cancer recurrence, *CSM* cancer-specific mortality, *DASH* Dietary Approaches to Stop Hypertension, *DGI* Australian Dietary Guideline Index, *DHDI* Dutch Healthy Diet Index, *DQIR* Diet Quality Index-Revised, *EnCoRe* energy for life after colorectal cancer, *GLCC* Guangdong Liver Cancer Cohort, *HEAL* Health Eating Activity and Lifestyle Study, *HEI* Healthy Eating Index, *HNFI* healthy Nordic Food Index, *hPDI* healthful plant-based diet index, *HPFS* Health Professionals Follow-up Study, *IWHS* Iowa Women’s Health Study, *MEC* Multiethnic Cohort, *MED* Mediterranean Diet Score, *NHANES III* Third National Health and Nutrition Examination Survey, *NHS* Nurses’ Health Study; *NIH-AARP* National Institutes of Health American Association of Retired Persons Diet and Health Study, *NLFCRC* Newfoundland and Labrador Familial Colorectal Cancer cohort, *OM* overall mortality, *OPAL* Ovarian cancer Prognosis And Lifestyle, *POST* postdiagnosis, *PRE* prediagnosis, *RFS* Recommended Food Score, *Ref* reference, *SBCSS* Shanghai Breast Cancer Survival Study, *WCRF* World Cancer Research Fund Score, *WHI* Women’s Health Initiative, *y* year.

Table S7: Overview of included studies in the meta-analysis analyzing healthy/prudent dietary patterns.

| **Author (y)** | **Cohort** | **Cancer type** | **PRE** | **POST** | **Term of healthy/prudent pattern(s)** | **OM** | **CSM** | **CR** |
| --- | --- | --- | --- | --- | --- | --- | --- | --- |
| Arthur et al. (2013) | UM HN-SPORE | Head and neck cancer | x |  | Whole food pattern | x |  | x |
| Fung et al. (2014) | NHS | Colorectal cancer |  | x | Prudent pattern | x | x |  |
| Guinter et al. (2018) | CPS-II Nutrition Cohort | Colorectal cancer | x | x | Prudent pattern | x | x |  |
| Kroenke et al. (2005) | NHS | Breast cancer |  | x | Prudent pattern | x | x |  |
| Kwan et al. (2009) | LACE | Breast cancer |  | x | Prudent pattern | x | x | x |
| Lee et al. (2020) | NHS & HPFS | Multiple myeloma | x |  | Prudent pattern | x | x |  |
| Lei et al. (2021) | HKNKBCSS | Breast cancer |  | x | Healthy pattern | x | x | x |
| Meyerhardt et al. (2007) | CALGB | Colon cancer |  | x | Prudent pattern | x |  | x |
| Ollberding et al. (2013) | Nebraska Lymphoma Study Group | Non-Hodgkin lymphoma | x |  | Fruit, vegetable and starch pattern | x |  |  |
| Sharma et al. (2018) | NLFCRC | Colorectal cancer | x |  | Prudent vegetable pattern | x |  |  |
| Van Blarigan et al. (2020) | CALGB/SWOG 80405 | Colorectal cancer | x |  | Prudent pattern | x |  |  |
| Vrieling et al. (2013) | MARIE study | Breast cancer | x |  | Healthy pattern | x | x | x |
| Wen et al. (2022) | OOPS | Ovarian cancer | x |  | Healthy pattern | x |  |  |
| Yang et al. (2015) | PHS I and II | Prostate cancer |  | x | Prudent pattern | x | x |  |
|  |  | **Summe:** | **8** | **7** | **14** | **14** | **8** | **5** |

Abbreviations: *CALGB* Cancer and Leukemia Group B, *CALGB/SWOG 80405* Cancer and Leukemia Group B/Southwest Oncology Group 80405, *CPS* Cancer Prevention Study, *CR* cancer recurrence, *CSM* cancer-specific mortality, *HKNKBCSS* Hong Kong NTEC-KWC Breast Cancer Survival Study, *HPFS* Health Professionals Follow-up Study, *LACE* Life After Cancer Epidemiology, *NHS* Nurses’ Health Study; *NLFCRC* Newfoundland and Labrador Familial Colorectal Cancer cohort, *OM* overall mortality, *OOPS* Ovarian cancer follow-up study, *PHS* Physicians' Health Study, *POST* postdiagnosis, *PRE* prediagnosis, *Ref* reference, *UM HN-SPORE* University of Michigan Head and Neck Specialized Program of Research Excellence, *y* year.

Table S8: Overview of included studies in the meta-analysis analyzing unhealthy/western dietary patterns.

| **Author (y)** | **Cohort** | **Cancer type** | **PRE** | **POST** | **Term of unhealthy/western pattern(s)** | **OM** | **CSM** | **CR** |
| --- | --- | --- | --- | --- | --- | --- | --- | --- |
| Arthur et al. (2013) | UM HN-SPORE | Head and neck cancer | x |  | Western pattern | x |  | x |
| Ferronha et al. (2012) | Patients of Hospital de S. João & IPO | Gastric cancer | x |  | Pattern 2, pattern 3 | x |  |  |
| Fung et al. (2014) | NHS | Colorectal cancer |  | x | Western pattern | x | x |  |
| Guinter et al. (2018) | CPS-II Nutrition Cohort | Colorectal cancer | x | x | Western pattern | x | x |  |
| Kroenke et al. (2005) | NHS | Breast cancer | x | x | Western pattern | x | x |  |
| Kwan et al. (2009) | LACE | Breast cancer |  | x | Western pattern | x | x | x |
| Lee et al. (2020) | NHS & HPFS | Multiple myeloma | x |  | Western pattern | x | x |  |
| Lei et al. (2021) | HKNKBCSS | Breast cancer |  | x | Western pattern | x | x | x |
| Meyerhardt et al. (2007) | CALGB | Colon cancer |  | x | Western pattern | x |  | x |
| Sharma et al. (2018) | NLFCRC | Colorectal cancer | x |  | Processed meat pattern, high-sugar pattern | x |  |  |
| Van Blarigan et al. (2020) | CALGB/SWOG 80405 | Colorectal cancer | x |  | Western pattern | x |  |  |
| Vrieling et al. (2013) | MARIE study | Breast cancer | x |  | Unhealthy pattern | x | x | x |
| Wen et al. (2022) | OOPS | Ovarian cancer | x |  | Ethnic pattern, animal foods pattern, sweet pattern | x |  |  |
| Yang et al. (2015) | PHS I and II | Prostate cancer |  | x | Western pattern | x | x |  |
|  |  | **Summe:** | **9** | **7** | **14** | **14** | **8** | **5** |

Abbreviations: *CALGB* Cancer and Leukemia Group B, *CALGB/SWOG 80405* Cancer and Leukemia Group B/Southwest Oncology Group 80405, *CPS* Cancer Prevention Study, *CR* cancer recurrence, *CSM* cancer-specific mortality, *HKNKBCSS* Hong Kong NTEC-KWC Breast Cancer Survival Study, *HPFS* Health Professionals Follow-up Study, *IPO* Instituto Português de Oncologia Francisco Gentil, *LACE* Life After Cancer Epidemiology, *NHS* Nurses’ Health Study; *NLFCRC* Newfoundland and Labrador Familial Colorectal Cancer cohort, *OM* overall mortality, *OOPS* Ovarian cancer follow-up study, *PHS* Physicians' Health Study, *POST* postdiagnosis, *PRE* prediagnosis, *Ref* reference, *UM HN-SPORE* University of Michigan Head and Neck Specialized Program of Research Excellence, *y* year.

Table S9: Detailed risk of bias judgement for each included study in the meta-analysis.

Conducted with the Cochrane Risk of bias in Non-randomized Studies of Interventions tool.

| **Domain and Questions** | **Study** | **Answer** |
| --- | --- | --- |
| **Bias due to confounding**   - Is there potential for confounding of the effect of exposure in this study?      - Did the authors use a multivariable-adjusted analysis method that controlled at least for age, sex, smoking, education/socioeconomic status, cancer stage, surgery/treatment and total energy intake? - Did the authors avoid adjusting for post-exposure variables?   Notes: Confounding is expected in all observational studies, low risk of bias was not assigned to any study. Time-varying confounding was expected to be unlikely and is not expected to cause risk of bias in the present study. | Al Ramadhani et al. (2021) | - Yes - Sex (participants were women only), education/socioeconomic status, cancer stage (model was stratified by cancer stage), surgery/treatment and total energy intake not adjusted for |
|  | Anyene et al. (2021) | - Yes - Sex (participants were women only), surgery/treatment not adjusted for |
|  | Arthur et al. (2013) | - Yes - Education/socioeconomic status not adjusted for, however: education was balanced and was assessed for collinearity and excluded in final models because it was highly correlated with other variables. |
|  | Deshmukh et al. (2018) | - Yes - Smoking, cancer stage, surgery/treatment and total energy intake not adjusted for |
|  | Di Maso et al. (2020) | - Yes - Sex (participants were women only), smoking, surgery/treatment (no participant received previous cancer treatment) not adjusted for |
|  | Di Maso et al. (2021) | - Yes - Sex (participants were men only), smoking, surgery/treatment (no participant received previous cancer treatment) not adjusted for |
|  | Ergas et al. (2021) | - Yes - Sex not adjusted for, however: participants were women only |
|  | Ferronha et al. (2012) | - Yes - Smoking not adjusted for because it did not influence the estimates meaningfully. Surgery/treatment not adjusted for, however: participants completed the questionnaire mostly before surgical treatment. |
|  | Fung et al. (2014) | - Yes - Sex (participants were women only), education/socioeconomic not adjusted for, however: education/socioeconomic status is not expected to vary substantially within the cohort (NHS) |
|  | George et al. (2011) | - Yes - Sex (participants were women only), age (used as the underlying time metric), smoking, education/socioeconomic status, cancer stage, surgery/treatment not adjusted for   "we included variables that improved model fit and changed the magnitude of hazard ratios by at least 10% and/or allowed comparison to the published literature."  It is not shown which variables have reached or not reached 10%. Therefore, it was classified as N.I.. |
|  | Guinter et al. (2018) | - Yes - Yes |
|  | Inoue-Choi et al. (2013) | - Yes - Sex (participants were women only), education/socioeconomic (however: not expected to vary substantially within the cohort (IWHS)), total energy intake not adjusted for |
|  | Izano et al. (2013) | - Yes - Sex (participants were women only), education/socioeconomic not adjusted for, however: education/socioeconomic status is not expected to vary substantially within the cohort (NHS) |
|  | Jacobs et al. (2016) | - Yes - Sex not adjusted for since men and women were analysed separately |
|  | Karavasiloglou et al. (2019) | - Yes - Sex (participants were women only), surgery/treatment not adjusted for |
|  | Kenfield et al. (2014) | - Yes - Sex (participants were men only), education/socioeconomic not adjusted for, however: education/socioeconomic status is not expected to vary substantially within the cohort (HPFS) |
|  | Kim et al. (2011) | - Yes - Sex (participants were women only), education/socioeconomic not adjusted for, however: education/socioeconomic status is not expected to vary substantially within the cohort (NHS) |
|  | Kroenke et al. (2005) | - Yes - Sex (participants were women only), education/socioeconomic not adjusted for, however: education/socioeconomic status is not expected to vary substantially within the cohort (NHS) |
|  | Kwan et al. (2009) | - Yes - Sex (participants were women only), education/socioeconomic not adjusted for, however: education/socioeconomic status is not expected to vary substantially within the cohort (LACE) |
|  | Lee et al. (2020) | - Yes - Sex (men and women were analysed separately and results were pooled), smoking, education/socioeconomic status (however: not expected to vary substantially within the cohort (NHS, HPFS)), cancer stage, surgery/treatment (data were not available for all participants) not adjusted for |
|  | Lei et al. (2021) | - Yes - Sex (participants were women only), smoking not adjusted for |
|  | Luo et al. (2020) | - Yes - Yes |
|  | McCullough et al. (2016) | - Yes - Sex (participants were women only), education/socioeconomic (tests showed no changes of the RRs) not adjusted for |
|  | Meyerhardt et al. (2007) | - Yes - Cancer stage (participants with stage III only were included), education/socioeconomic not adjusted for, however: education/socioeconomic status is not expected to vary substantially within the cohort (CALGB) |
|  | Ollberding et al. (2013) | - Yes - Education/socioeconomic status, cancer stage, surgery/treatment not adjusted for, however: subgroup analyses were adjusting for established clinical prognostic factors including initial treatment, Ann Arbor stage at diagnosis (I, II, III, IV) and the presence of B-symptoms at diagnosis (yes/no) |
|  | Park et al. (2022) | - Yes - Yes |
|  | Pelser et al. (2014) | - Yes - Yes |
|  | Ratjen et al. (2017) | - Yes - Cancer stage, education/socioeconomic not adjusted for |
|  | Ratjen et al. (2021) | - Yes - Cancer stage, education/socioeconomic not adjusted for |
|  | Sharma et al. (2018) | - Yes - Smoking (was tested but showed no detectable effect in the association), education/socioeconomic status not adjusted for |
|  | Song et al. (2021) | - Yes - Education/socioeconomic status (however: not expected to vary substantially within the cohort (NHS, HPFS)), surgery/treatment not adjusted for   All models were stratified by age and cancer stage |
|  | Sun et al. (2018) | - Yes - Sex (participants were women only), surgery/treatment not adjusted for |
|  | Thomson et al. (2014) | - Yes - Sex (participants were women only), education/socioeconomic, surgery/treatment not adjusted for |
|  | Van Blarigan et al. (2020) | - Yes - Smoking, education/socioeconomic not adjusted for |
|  | Van Zutphen et al. (2021) | - Yes - total energy intake (was tested but made <5% differences to the results) not adjusted for |
|  | Vrieling et al. (2013) | - Yes - Sex (participants were women only), education/socioeconomic, surgery/treatment, smoking not adjusted for, however: these variables were not significant and did not change the risk estimates by >10% and were therefore not included in the final model |
|  | Wang et al. (2020) | - Yes - Sex (participants were women only) not adjusted for |
|  | Wen et al. (2022) | - Yes - Sex (participants were women only), surgery/treatment not adjusted for |
|  | Yang et al. (2015) | - Yes - Sex (participants were women only), education/socioeconomic not adjusted for |
| **Bias due to selection of participants**   - Was selection of participants into the study based on participants characteristics observed after start of the study/exposure assessment? - Do start of follow-up and start of exposure coincide for most participants? Were methods used that are likely to correct for the presence of selection biases?   Notes: In observational studies, it is unlikely that post-exposure variables influenced selection of participants into the study. Exclusion of participants may be mostly based on missing data, which will be considered in the domain referring to missings (see below). The start of follow-up is considered to coincide with the baseline exposure assessment. However, participants are already exposed at start of the study which might have influenced outcome measured that occurred shortly after start of the study. | Al Ramadhani et al. (2021) | - No - Yes - Inclusion of a six or 12-month lag between the measurement of diet quality and start of follow-up showed similarly results; |
|  | Anyene et al. (2021) | - No - Yes - no subgroup analysis excluding participants who died <1 year after diagnosis; |
|  | Arthur et al. (2013) | - No - Yes - no subgroup analysis excluding participants who died <1 year after diagnosis; |
|  | Deshmukh et al. (2018) | - No - Yes - sensitivity analysis was performed where deaths that occurred within a five-year follow-up window were censored and it was consistent with the main analysis; |
|  | Di Maso et al. (2020) | - No - Yes - no subgroup analysis excluding participants who died <1 year after diagnosis; |
|  | Di Maso et al. (2021) | - No - Yes - no subgroup analysis excluding participants who died <1 year after diagnosis; |
|  | Ergas et al. (2021) | - No - Yes - no subgroup analysis excluding participants who died <1 year after diagnosis; |
|  | Ferronha et al. (2012) | - No - Yes - no subgroup analysis excluding participants who died <1 year after diagnosis; No changes when excluding patients who died up to 15 days after surgery; |
|  | Fung et al. (2014) | - No - Yes - participants who died within 6 months after the return of the first post-diagnosis biennial questionnaire were excluded and "additional analysis excluding survivors who died within one year of diagnosis did not materially change the results"; |
|  | George et al. (2011) | - No - Yes - "When we excluded women who had events in the first year of follow-up, the magnitude of HRs were similar"; |
|  | Guinter et al. (2018) | - No - Yes - In the postdiagnosis models, participants who completed their questionnaire within 12 months of diagnosis were excluded in a sensitivity analysis and no substantive differences were observed; |
|  | Inoue-Choi et al. (2013) | - No - Yes - Participants who were diagnosed with cancer within 2 years prior to the questionnaire were excluded; - Inclusion of postmenopausal women only, because the main cohort included postmenopausal women only; |
|  | Izano et al. (2013) | - No - Yes - "Women whose disease recurred or who died less than a year after breast cancer diagnosis were excluded from analyses."; |
|  | Jacobs et al. (2016) | - No - Yes - no subgroup analysis excluding participants who died <1 year after diagnosis; |
|  | Karavasiloglou et al. (2019) | - No - Yes - no subgroup analysis excluding participants who died <1 year after diagnosis, however: exposure assessment occurred on average 10.4 years after diagnosis. |
|  | Kenfield et al. (2014) | - No - Yes - no subgroup analysis excluding participants who died <1 year after diagnosis; |
|  | Kim et al. (2011) | - No - Yes - "Women with any death or recurrence within one year of diagnosis were excluded."; |
|  | Kroenke et al. (2005) | - No - Yes - participants with breast cancer deaths that occurred within a year of diagnosis were excluded; |
|  | Kwan et al. (2009) | - No - Yes - no subgroup analysis excluding participants who died <1 year after diagnosis, however: participants entered the cohort over an approximately 3-year period since diagnosis; |
|  | Lee et al. (2020) | - No - Yes - patients who died during a 2-year lag to pre-diagnosis were excluded; |
|  | Lei et al. (2021) | - No - Yes - no subgroup analysis excluding participants who died <1 year after diagnosis; |
|  | Luo et al. (2020) | - No - Yes - Sensitivity analysis was conducted by excluding participants who died within 90 days during their follow‐up and did not alter the results; |
|  | McCullough et al. (2016) | - No - Yes - Sensitivity analyses showed similar results after excluding women who were diagnosed 2 years before post-diagnostic diet reporting and excluding the first 2 years of follow-up; |
|  | Meyerhardt et al. (2007) | - No - Yes - Patients who developed cancer recurrence or died within 90 days after the first FFQ were excluded in the primary analyses; Exclusion of patients who developed cancer recurrence or died within 180 days after the first FFQ didn't change the results largely; - Inclusion of participants with stage III colon cancer only because the main cohort included participants with stage III colon cancer only; |
|  | Ollberding et al. (2013) | - No - Yes - Patients who died within 6 months of the date of diagnosis were excluded; |
|  | Park et al. (2022) | - No - Yes - A sensitivity analysis without individuals who died within 2 years after the 10-year follow-up was conducted and the findings remained similar; |
|  | Pelser et al. (2014) | - No - Yes - no subgroup analysis excluding participants who died <1 year after diagnosis; |
|  | Ratjen et al. (2017) | - No - Yes - Sensitivity analyses were performed excluding participants who died within 12 months of diet assessment and results were largely unchanged, however: exposure assessment occurred 6 years (median) after diagnosis; |
|  | Ratjen et al. (2021) | - No - Yes - Sensitivity analyses showed slightly weaker but similar associations after excluding individuals who died within 12 months of the diet; |
|  | Sharma et al. (2018) | - No - Yes - no subgroup analysis excluding participants who died <1 year after diagnosis; |
|  | Song et al. (2021) | - No - Yes - Sensitivity analyses were conducted excluding the first six months and the first three years of follow-up after the post-diagnostic assessment; the findings remained similar; |
|  | Sun et al. (2018) | - No - Yes - sensitivity analyses excluding women who died within 2 years after completion of post-diagnosis FFQ were conducted; "the results became statistically insignificant", but no exclusion within the main results was done. Therefore, moderate risk was chosen; - Inclusion of postmenopausal women only, because the main cohort included postmenopausal women only; |
|  | Thomson et al. (2014) | - No - Yes - no subgroup analysis excluding participants who died <1 year after diagnosis; - Inclusion of postmenopausal women only, because the main cohort included postmenopausal women only; |
|  | Van Blarigan et al. (2020) | - No - Yes - A sensitivity analysis excluding patients who died within 90 days after administration of the diet did not change the results; |
|  | Van Zutphen et al. (2021) | - No - Yes - no subgroup analysis excluding participants who died <1 year after diagnosis; - Exposure assessment occurred 6 months after diagnosis or 6 months after treatment. |
|  | Vrieling et al. (2013) | - No - Yes - no subgroup analysis excluding participants who died <1 year after diagnosis; - Inclusion of postmenopausal women only, because the main cohort included postmenopausal women only; |
|  | Wang et al. (2020) | - No - Yes - no subgroup analysis excluding participants who died <1 year after diagnosis; |
|  | Wen et al. (2022) | - No - Yes - no subgroup analysis excluding participants who died <1 year after diagnosis; |
|  | Yang et al. (2015) | - No - Yes - sensitivity analyses excluding participants who died within 2 years after FFQ were conducted; |
| **Bias due to exposure assessment**   - Were exposure groups clearly defined and adequately assessed? - Was the information used to define the exposure groups based on reasonable a priori data?   Note: The start of follow-up is considered to coincide with the baseline exposure assessment. Any dietary assessment method involves measurement error ^104^, no study was assigned low risk of bias. | Al Ramadhani et al. (2021) | - Yes, use of validated FFQ - Categories: Tertile |
|  | Anyene et al. (2021) | - Yes, use of a modified version of a validated FFQ - Categories: Per 10-unit increase |
|  | Arthur et al. (2013) | - Yes, use of validated FFQ - Categories: Quintiles |
|  | Deshmukh et al. (2018) | - No, use of a single 24-hour dietary recall - Categories: Quartile |
|  | Di Maso et al. (2020) | - Yes, use of validated FFQ - Categories: Tertile |
|  | Di Maso et al. (2021) | - Yes, use of validated FFQ - Categories: High vs. low adherence |
|  | Ergas et al. (2021) | - Yes, use of a modified version of a validated FFQ - Categories: Quantile |
|  | Ferronha et al. (2012) | - Yes, use of validated FFQ - Categories: Pattern I was the reference |
|  | Fung et al. (2014) | - Yes, use of validated FFQ - Categories: Quintiles |
|  | George et al. (2011) | - Yes, use of validated FFQ - Categories: Quartile |
|  | Guinter et al. (2018) | - Yes, use of validated FFQ - Categories: Quartile |
|  | Inoue-Choi et al. (2013) | - Yes, use of validated FFQ - Categories: Tertile |
|  | Izano et al. (2013) | - Yes, use of validated FFQ - Categories: Quintile |
|  | Jacobs et al. (2016) | - Yes, use of validated FFQ - Categories: Quartile |
|  | Karavasiloglou et al. (2019) | - No, use of a 24-hour dietary recall interview - Categories: High vs. Low |
|  | Kenfield et al. (2014) | - Yes, use of validated FFQ - Categories: MED: Tertile; aMED: Quintile |
|  | Kim et al. (2011) | - Yes, use of validated FFQ - Categories: Quintile |
|  | Kroenke et al. (2005) | - Yes, use of validated FFQ - Categories: Quintile |
|  | Kwan et al. (2009) | - Yes, use of validated FFQ - Categories: Quartile |
|  | Lee et al. (2020) | - Yes, use of validated FFQ - Categories: Tertile |
|  | Lei et al. (2021) | - Yes, use of validated FFQ - Categories: Tertile |
|  | Luo et al. (2020) | - Yes, use of validated FFQ - Categories: Tertile |
|  | McCullough et al. (2016) | - Yes, use of a modified version of a validated FFQ - Categories: Tertile |
|  | Meyerhardt et al. (2007) | - Yes, use of validated FFQ - Categories: Quintile |
|  | Ollberding et al. (2013) | - Yes, us of validated FFQ - Categories: Tertile |
|  | Park et al. (2022) | - Yes, use of validated FFQ - Categories: Quartile |
|  | Pelser et al. (2014) | - Yes, use of validated FFQ - Categories: Quintile |
|  | Ratjen et al. (2017) | - Yes, use of validated FFQ - Categories: Quartile |
|  | Ratjen et al. (2021) | - Yes, use of validated FFQ - Categories: Quintile |
|  | Sharma et al. (2018) | - Yes, use of validated FFQ - Categories: Quartile |
|  | Song et al. (2021) | - Yes, use of validated FFQ - Categories: Quartile |
|  | Sun et al. (2018) | - Yes, use of validated FFQ - Categories: Quartile |
|  | Thomson et al. (2014) | - Yes, use of validated FFQ - Categories: Tertile |
|  | Van Blarigan et al. (2020) | - Yes, use of validated FFQ - Categories: Quantile |
|  | Van Zutphen et al. (2021) | - yes, use of validated FFQ and a 7-day dietary record - Categories: WCRF/AICR and ACS: Tertile; DHDI: Quartile |
|  | Vrieling et al. (2013) | - Yes, use of validated FFQ - Categories: Quartile |
|  | Wang et al. (2020) | - Yes, use of validated FFQ - Categories: Quartile |
|  | Wen et al. (2022) | - Yes, use of validated FFQ - Categories: Tertile |
|  | Yang et al. (2015) | - Yes, use of a modified version of a validated FFQ - Categories: Quartile |
| **Bias due to misclassification during follow-up**   - Were there deviations from the exposure beyond what would be expected in usual practice? - Were these deviations unbalanced between groups and likely to have affected the outcome?   Notes: Repeated measurements of the exposure are mostly not available in observational studies. It is not expected that there are high changes in diet in cancer participants. Changes in diet may be similar between studies and may also be similar between groups (differential misclassification is not expected). Recent studies have shown that changes in overall lifestyle (including changes in diet) occur after cancer diagnosis, but these changes are only slightly on average, even after individual consultation.^95,126,127,128^ Thus, if repeated measures are not available, moderate risk of bias could be assigned to a study. | Al Ramadhani et al. (2021) | - No repeated measurements of the exposure status during follow-up are available, but high changes are not expected during follow-up (compare notes) |
|  | Anyene et al. (2021) | - Repeated measurements of the exposure status during follow-up are available and cumulative average of post-diagnosis diet was used |
|  | Arthur et al. (2013) | - No repeated measurements of the exposure status during follow-up are available, but high changes are not expected during follow-up (compare notes) |
|  | Deshmukh et al. (2018) | - No repeated measurements of the exposure status during follow-up are available, but high changes are not expected during follow-up (compare notes) |
|  | Di Maso et al. (2020) | - No repeated measurements of the exposure status during follow-up are available, but high changes are not expected during follow-up (compare notes) |
|  | Di Maso et al. (2021) | - No repeated measurements of the exposure status during follow-up are available, but high changes are not expected during follow-up (compare notes) |
|  | Ergas et al. (2021) | - No repeated measurements of the exposure status during follow-up are available, but high changes are not expected during follow-up (compare notes) |
|  | Ferronha et al. (2012) | - No repeated measurements of the exposure status during follow-up are available, but high changes are not expected during follow-up (compare notes) |
|  | Fung et al. (2014) | - Repeated measurements of exposure are available, however, "in this analysis, we used the first FFQ that was collected at least 6 months after diagnosis to minimize dietary intake affected by active treatment." |
|  | George et al. (2011) | - No repeated measurements of the exposure status during follow-up are available, but high changes are not expected during follow-up (compare notes) |
|  | Guinter et al. (2018) | - Repeated measurements of the exposure status during follow-up are available and were considered in the sensitivity analyses. No substantive differences were observed |
|  | Inoue-Choi et al. (2013) | - No repeated measurements of the exposure status during follow-up are available, but high changes are not expected during follow-up (compare notes) |
|  | Izano et al. (2013) | - Repeated measurements of the exposure status during follow-up are available and cumulative averages of the diet scores from repeated FFQs were computed |
|  | Jacobs et al. (2016) | - Repeated measurements of the exposure status during follow-up are available and "correlations between prediagnostic (Qx1) and postdiagnostic (Qx3) dietary index scores indicated acceptable consistency" |
|  | Karavasiloglou et al. (2019) | - No repeated measurements of the exposure status during follow-up are available, but high changes are not expected during follow-up (compare notes) |
|  | Kenfield et al. (2014) | - Repeated measurements of the exposure status during follow-up are available and cumulative average post-diagnostic dietary intakes from the most recent FFQ preceding diagnosis until the end of follow-up were calculated. |
|  | Kim et al. (2011) | - Repeated measurements of exposure are available, however, only the FFQ at least 12 months after diagnosis was used. High changes are not expected during follow-up (compare notes) - "Cumulative updating of diet indices after diagnosis was not used because of potential bias of decreased intake or changes in diet due to a recurrence." |
|  | Kroenke et al. (2005) | - Repeated measurements of the exposure status during follow-up are available. Three sets of analyses were conducted for pre-diagnosis diet and post-diagnosis diet. However, for post-diagnosis only the FFQ at least 12 months after diagnosis was used. High changes are not expected during follow-up (compare notes) |
|  | Kwan et al. (2009) | - No repeated measurements of the exposure status during follow-up are available, but high changes are not expected during follow-up (compare notes) |
|  | Lee et al. (2020) | - Repeated measurements of the exposure status during follow-up are available. Sensitivity analysis showed similar associations. Additionally, Spearman correlation coefficients between the most recent and the cumulative average prediagnosis dietary patterns, and between the most recent prediagnosis and the first postdiagnosis dietary patterns were calculated and showed high correlations. |
|  | Lei et al. (2021) | - No repeated measurements of the exposure status during follow-up are available, but high changes are not expected during follow-up (compare notes) |
|  | Luo et al. (2020) | - No repeated measurements of the exposure status during follow-up are available, but high changes are not expected during follow-up (compare notes) |
|  | McCullough et al. (2016 | - Repeated measurements of the exposure status during follow-up are available (not the same amount and not for all patients) and for post-diagnosis diet the FFQ returned at least 1 year after the participant’s diagnosis was used. Changes were not considered in the analysis, however: pre- and post-diagnosis diet were analysed separately and high changes are not expected during follow-up (compare notes) |
|  | Meyerhardt et al. (2007) | - Repeated measurements of the exposure status during follow-up are available and cumulative averaging was used |
|  | Ollberding et al. (2013) | - No repeated measurements of the exposure status during follow-up are available, but high changes are not expected during follow-up (compare notes) |
|  | Park et al. (2022) | - Repeated measurements of the exposure status during follow-up are available. Changes in diet quality were obtained in comparison to no changes in diet quality only |
|  | Pelser et al. (2014) | - No repeated measurements of the exposure status during follow-up are available, but high changes are not expected during follow-up (compare notes) |
|  | Ratjen et al. (2017) | - No repeated measurements of the exposure status during follow-up are available, but high changes are not expected during follow-up (compare notes) |
|  | Ratjen et al. (2021) | - No repeated measurements of the exposure status during follow-up are available, but high changes are not expected during follow-up (compare notes) |
|  | Sharma et al. (2018) | - No repeated measurements of the exposure status during follow-up are available, but high changes are not expected during follow-up (compare notes) |
|  | Song et al. (2021) | - Repeated measurements of the exposure status during follow-up are available. Changes were considered in the analysis (HR adjusted for pre-diagnostic WCRF/AICR diet score) |
|  | Sun et al. (2018) | - Repeated measurements of the exposure status during follow-up are available (not the same amount and not for all patients) and the FFQs administered the closest in time to before and after breast cancer diagnosis was used. Additionally, analyses showed that most (72%) of the participants maintained relatively stable diet quality after breast cancer diagnosis |
|  | Thomson et al. (2014) | - Repeated measurements of the exposure status during follow-up are available (not the same amount and not for all patients) and the average measured at least 12 months prior to diagnosis was used |
|  | Van Blarigan et al. (2020) | - No repeated measurements of the exposure status during follow-up are available, but high changes are not expected during follow-up (compare notes) |
|  | Van Zutphen et al. (2021) | - Repeated measurements of the exposure status during follow-up are available and only slight changes in diet quality were observed |
|  | Vrieling et al. (2013) | - No repeated measurements of the exposure status during follow-up are available, but high changes are not expected during follow-up (compare notes) |
|  | Wang et al. (2020) | - No repeated measurements of the exposure status during follow-up are available, but high changes are not expected during follow-up (compare notes) |
|  | Wen et al. (2022) | - No repeated measurements of the exposure status during follow-up are available, but high changes are not expected during follow-up (compare notes) and Adjustment for dietary change was made (patients were asked if they have changed their diet) |
|  | Yang et al. (2015) | - No repeated measurements of the exposure status during follow-up are available, but high changes are not expected during follow-up (compare notes) |
| **Bias due to missing data**   - Were there missing outcome data? - Were participants excluded due to missing data on exposure status? - Were participants excluded due to missing data on other variables needed for analysis?   Notes: Missing data on exposure variables and other variables are expected to be missing at random and not related to exposure or outcome that have been assessed during follow-up. | Al Ramadhani et al. (2021) | - "Clinical data were abstracted from women’s medical records, surgical and pathology report and information about disease recurrence, vital status and cause of death were collected annually from medical records."   Therefore, no loss-to-follow up   - based on included cases in analysis, data are reasonably complete |
|  | Anyene et al. (2021) | - Outcome data were obtained by follow-up health status questionnaires, medical record searches, mortality files and linkage to National Death Index.   Therefore, no loss-to-follow up   - based on included cases in analysis, data are reasonably complete |
|  | Arthur et al. (2013) | - No loss-to-follow up since the follow up includes medical record reviews and the Social Security Death Index only and no response of participants was required - "Dietary intake data were assessed for missing values and energy outliers by using standard techniques" - based on included cases in analysis, data are reasonably complete |
|  | Deshmukh et al. (2018) | - "Mortality,…,was obtained from the National Center for Health Statistics Linked Mortality Files."   Therefore, no loss-to-follow up   - based on included cases in analysis, data are reasonably complete |
|  | Di Maso et al. (2020) | - "The vital status, the date of death, and the underlying cause of death were ascertained through a record-linkage procedure with the population-based regional cancer registries"   Therefore, no loss-to-follow up   - based on included cases in analysis, data are reasonably complete |
|  | Di Maso et al. (2021) | - "The vital status, the date, and the underlying cause of death ,..., were ascertained ,..., through a record-linkage procedure with the population-based regional cancer registries"   Therefore, no loss-to-follow up   - based on included cases in analysis, data are reasonably complete |
|  | Ergas et al. (2021) | - Outcome data were confirmed by medical chart review or were identified from linkages with data from the state of California, the Social Security Administration, and the National Death Index.   Therefore, no loss-to-follow up   - based on included cases in analysis, data are reasonably complete |
|  | Ferronha et al. (2012) | - No loss-to-follow up since patients were excluded, if no follow-up information could be obtained - based on included cases in analysis, data are reasonably complete |
|  | Fung et al. (2014) | - "The follow-up for death was over 95% complete" - based on included cases in analysis, data are reasonably complete |
|  | George et al. (2011) | - No loss-to-follow up since the follow-up occurred through obtaining state mortality files and the National Death Index only and no response from participants was required - based on included cases in analysis, data are reasonably complete |
|  | Guinter et al. (2018) | - "Vital status, cause of death, and date of death were determined through linkage to the National Death Index. Cause of death was obtained for 99.3% of all known deaths in the Cohort." - based on included cases in analysis, data are reasonably complete |
|  | Inoue-Choi et al. (2013) | - No loss-to-follow up since the vital status of the cohort participants was updated annually via linkage with the State Health Registry of Iowa, supplemented with the National Death Index - based on included cases in analysis, data are reasonably complete |
|  | Izano et al. (2013) | - "Deaths were reported by family members, the postal service, or searches in the National Death Index for questionnaire non-responders.", "The ascertainment of the cause of death in this cohort is estimated to be 98%." - based on included cases in analysis, data are reasonably complete |
|  | Jacobs et al. (2016) | - "Dates and causes of death were identified by routine linkages with California and Hawaii vital records and the National Death Index databases."   Therefore, no loss-to-follow up   - For covariates with missing values a missing category was created. - based on included cases in analysis, data are reasonably complete |
|  | Karavasiloglou et al. (2019) | - Mortality was obtained from National Death Index.   Therefore, no loss-to-follow up   - based on included cases in analysis, data are reasonably complete |
|  | Kenfield et al. (2014) | - "Deaths were identified from family reports and National Death Index searches; we ascertained >98% of deaths. Causes of death were adjudicated by study physicians who reviewed medical records and death certificates." - based on included cases in analysis, data are reasonably complete |
|  | Kim et al. (2011) | - "Deaths were reported by family members or the postal service, or were identified from a search of the National Death Index; the ascertainment of death is estimated to be 98% complete. The primary cause of death was determined by physicians reviewers." - based on included cases in analysis, data are reasonably complete |
|  | Kroenke et al. (2005) | - "Ascertainment of deaths in the NHS cohort included reporting by the family or postal authorities. Additionally, names of persistent nonresponders were searched in the National Death Index.", "The cause of death was assigned by physician reviewers.", "More than 98% of deaths in the NHS cohort have been identified by this method." - based on included cases in analysis, data are reasonably complete |
|  | Kwan et al. (2009) | - "Medical records were reviewed to verify reported outcomes. Participant deaths were determined through KPNC electronic data sources, a family member responding to a mailed questionnaire, or a phone call." , "copies of death certificates were obtained to confirm cause of death."   Therefore, no loss-to-follow up.   - based on included cases in analysis, data are reasonably complete |
|  | Lee et al. (2020) | - "Deaths were identified by next of kin, the postal system or routine searches of the National Death Index, methods which have shown greater than 98% sensitivity in the NHS and HPFS. Reviewers blinded to exposures reviewed the corresponding medical records to assign the cause of death." - based on included cases in analysis, data are reasonably complete |
|  | Lei et al. (2021) | - "Information about disease progression and survival status was collected by reviewing medical records annually."   Therefore, no loss-to-follow up   - based on included cases in analysis, data are reasonably complete |
|  | Luo et al. (2020) | - "Death and causes of death were ascertained by referring to the death registration and reporting system of the Guangdong Provincial Center for Disease Control and Prevention, combined with the inpatient and outpatient medical system of Sun Yat‐sen University Cancer Center. In addition, we called or sent mail to participants or their surrogates to confirm their survival status."   Therefore, no loss-to-follow up   - based on included cases in analysis, data are reasonably complete |
|  | McCullough et al. (2016) | - Outcome data were determined by linkage to the National Death Index and cause of death has been obtained for 99.3 % of all known deaths. - based on included cases in analysis, data are reasonably complete |
|  | Meyerhardt et al. (2007) | - No information available on how outcome data was collected and if outcome data is missing - "Covariates with missing variables were coded with indicator variables in adjusted models." - based on included cases in analysis, data are reasonably complete |
|  | Ollberding et al. (2013) | - "Vital status was obtained through active patient follow-up supplemented by data linkages to the Nebraska state death certificate files.", Only patients with available follow-up data were included.   Therefore, no loss-to-follow up.   - "Patients with missing data on the key covariates educational attainment and smoking status, or who died within 6 months of the date of diagnosis, were also excluded." - based on included cases in analysis, data are reasonably complete |
|  | Park et al. (2022) | - "Deaths were identified by linkage to Hawaii and California death files and the National Death Index"   Therefore, no loss-to-follow up   - based on included cases in analysis, data are reasonably complete |
|  | Pelser et al. (2014) | - "Mortality and cause of death were ascertained by linkage with the Social Security Administration Death Master File and the National Death Index Plus."   Therefore, no loss-to-follow up.   - based on included cases in analysis, data are reasonably complete |
|  | Ratjen et al. (2017) | - "Participants who did not respond or for whom spouses reported the study participant's death, vital status was obtained from population registries, and the date of death was recorded."   Therefore, no loss-to-follow up   - Individuals with missing information on the year of diagnosis, vital status and participants for whom information on follow-up length was implausible were excluded. - based on included cases in analysis, data are reasonably complete |
|  | Ratjen et al. (2021) | - Vital status was updated via population registries. "the date of death could be verified for all cases." - Therefore, no loss-to-follow up - based on included cases in analysis, data are reasonably complete |
|  | Sharma et al. (2018) | - "We conducted follow-up questionnaires with participants and linked records to death certificates, pathology reports, autopsy records, physicians’ notes, and surgical reports. Additional data were obtained from the Dr. H. Bliss Murphy Cancer Care Foundation;"   "Individuals who were lost to follow up,...,were censored at the time of the last contact"  Therefore, no loss-to-follow up   - based on included cases in analysis, data are reasonably complete |
|  | Song et al. (2021) | - "Deaths were identified through review of the National Death Index, postal authorities, or the next-of-kin in response to the follow-up questionnaires." The cause of death was identified by reviewing death certificates and medical records.   Therefore, no loss-to-follow up   - Missing data for any covariate was less than 3 % and based on included cases in analysis, data are reasonably complete |
|  | Sun et al. (2018) | - Medical records, National Death Index and death certificate were reviewed.   Therefore, no loss-to-follow up   - based on included cases in analysis, data are reasonably complete |
|  | Thomson et al. (2014) | - "Death of any cause was ascertained from proxy report, or vital status was determined through linkage with the National Death Index with cause of death coded according to the International Classification of Diseases."   Therefore, no loss-to-follow up   - based on included cases in analysis, data are reasonably complete |
|  | Van Blarigan et al. (2020) | - No information available on how outcome data was collected and if outcome data is missing - "A missing indicator was used to account for missing data in KRAS and tumor sidedness."   Missing data on other variables were assigned to the most common categories for multivariate adjustment.   - based on included cases in analysis, data are reasonably complete |
|  | Van Zutphen et al. (2021) | - "Vital status and date of death were determined through linkage to the Municipal Personal Record Database of the Netherlands"   Therefore, no loss-to-follow up.   - based on included cases in analysis, data are reasonably complete |
|  | Vrieling et al. (2013) | - "vital status was determined through population registries" and "Causes of death were extracted from death certificates. Medical records were checked or treating physicians were contacted to identify recurrences and to verify self-reported events collected during a follow-up telephone interview,..., (90% self-reported events verified)." - based on included cases in analysis, data are reasonably complete |
|  | Wang et al. (2020) | - "Information on breast cancer recurrence/metastasis, mortality and cause of death for current study participants was collected during the 10-year postdiagnosis in-person survey, with a response rate of 93.8%. Survival status and cause of death were supplemented by periodic record linkage to the Shanghai Vital Statistics Registry" - based on included cases in analysis, data are reasonably complete |
|  | Wen et al. (2022) | - "The vital status of participants were obtained by medical records and active follow-up."   Therefore, no loss-to-follow up   - based on included cases in analysis, data are reasonably complete |
|  | Yang et al. (2015) | - "Deaths were identified by reports from family members and postal authorities, and systematic searches of the National Death Index,..., confirmed through review of death certificates and medical records,..., When medical records cannot be obtained, cause of death is assigned upon reviews of all other available data by the Endpoints Committee."   Therefore, no loss-to-follow up.   - based on included cases in analysis, data are reasonably complete |
| **Bias due to measurement of the outcome**   - Could the outcome measure have been influenced by knowledge of the exposure status? - Were the methods of outcome assessment comparable across exposure groups? - Were any systematic error in measurement of the outcome related to exposure status?   Notes: In observational studies, it is not expected that outcome assessors were aware of exposure status of the participants. | Al Ramadhani et al. (2021) | - outcome assessment the same in all groups - "Information about disease recurrence, vital status and cause of death were collected annually from medical records." |
|  | Anyene et al. (2021) | - outcome assessment the same in all groups - "Breast cancer recurrences were identified using a combination of follow-up health status questionnaires and Kaiser Permanente Northern California electronic medical record searches. Mortality and causes of death were ascertained from Kaiser Permanente Northern California’s Virtual Data Warehouse mortality files, which incorporate internal data from the Kaiser Permanente Northern California health system, and external linkages with mortality information from the State of California, the Social Security Administration, and the National Death Index" |
|  | Arthur et al. (2013) | - outcome assessment the same in all groups - "Death and recurrence data were obtained from medical records and the Social Security Death Index" |
|  | Deshmukh et al. (2018) | - outcome assessment the same in all groups - "Mortality was obtained from the National Center for Health Statistics Linked Mortality Files." |
|  | Di Maso et al. (2020) | - outcome assessment the same in all groups - "The vital status, the date of death, and the underlying cause of death were ascertained through a record-linkage procedure with the population-based regional cancer registries covering the areas where patients were enrolled." |
|  | Di Maso et al. (2021) | - outcome assessment the same in all groups - "The vital status, the date, and the underlying cause of death (i.e., the condition that led to death) were ascertained up to 31 December 2017 through a record-linkage procedure with the population-based regional cancer registries of Friuli Venezia Giulia and Veneto regions" |
|  | Ergas et al. (2021) | - outcome assessment the same in all groups - "Recurrences were ascertained either during follow-up interviews with participants or from monthly algorithmic searches of KPNC electronic databases. All recurrences were confirmed by medical chart review. Deaths and causes of death were identified during follow-up interviews with relatives of participants and then confirmed by medical chart review or from linkages with data from the state of California, the Social Security Administration, and the National Death Index." |
|  | Ferronha et al. (2012) | - outcome assessment the same in all groups - "The vital status of the participants was assessed by the RORENO ( North Region Cancer Registry)." |
|  | Fung et al. (2014) | - outcome assessment the same in all groups - "Death was ascertained from state vital statistics records, the National Death Index, and through review of death certificates that were submitted via post by the deceased participants’ next-of-kin." |
|  | George et al. (2011) | - outcome assessment the same in all groups - "We used SEER cancer registry data from New Mexico, Los Angeles County, and Western Washington to determine vital status. We obtained data on underlying cause of death from state mortality files and the National Death Index." |
|  | Guinter et al. (2018) | - outcome assessment the same in all groups - "Vital status, cause of death, and date of death were determined through linkage to the National Death Index." |
|  | Inoue-Choi et al. (2013) | - outcome assessment the same in all groups - "Vital status of the cohort participants is updated annually via linkage with the State Health Registry of Iowa, supplemented with the National Death Index" |
|  | Izano et al. (2013) | - outcome assessment the same in all groups - "Deaths were reported by family members, the postal service, or searches in the National Death Index for questionnaire non-responders. Cause of death was ascertained by physicians’ review of death certificates and medical records when needed. The ascertainment of the cause of death in this cohort is estimated to be 98%" |
|  | Jacobs et al. (2016) | - outcome assessment the same in all groups - "Dates and causes of death were identified by routine linkages with California and Hawaii vital records and the National Death Index databases." |
|  | Karavasiloglou et al. (2019) | - outcome assessment the same in all groups - "Probabilistic linkage of the NHANES III with the National Death Index, maintained by the National Center for Health Statistics, was used to obtain mortality information." |
|  | Kenfield et al. (2014) | - outcome assessment the same in all groups - "Deaths were identified from family reports and National Death Index searches; we ascertained >98% of deaths. Causes of death were adjudicated by study physicians who reviewed medical records and death certificates." |
|  | Kim et al. (2011) | - outcome assessment the same in all groups - "Deaths were reported by family members or the postal service, or were identified from a search of the National Death Index; the ascertainment of death is estimated to be 98% complete. The primary cause of death was determined by physicians reviewers." |
|  | Kroenke et al. (2005) | - outcome assessment the same in all groups - "Ascertainment of deaths in the NHS cohort included reporting by the family or postal authorities. Additionally, names of persistent nonresponders were searched in the National Death Index."   "The cause of death was assigned by physician reviewers."  "More than 98% of deaths in the NHS cohort have been identified by this method." |
|  | Kwan et al. (2009) | - outcome assessment the same in all groups - Medical records were reviewed to verify reported outcomes.   "Participant deaths were determined through Kaiser Permanente Northern California electronic data sources, a family member responding to a mailed questionnaire, or a phone call."  Copies of death certificates were obtained to confirm cause of death. |
|  | Lee et al. (2020) | - outcome assessment the same in all groups - "Deaths were identified by next of kin, the postal system or routine searches of the National Death Index.", "Reviewers blinded to exposures reviewed the corresponding medical records to assign the cause of death." |
|  | Lei et al. (2021) | - outcome assessment the same in all groups - "Information about disease progression and survival status was collected by reviewing medical records annually" |
|  | Luo et al. (2020) | - outcome assessment the same in all groups - "Death and causes of death were ascertained by referring to the death registration and reporting system of the Guangdong Provincial Center for Disease Control and Prevention, combined with the inpatient and outpatient medical system of Sun Yat‐sen University Cancer Center." |
|  | McCullough et al. (2016) | - outcome assessment the same in all groups - "Vital status of participants was determined through December 31, 2012 by linkage to the National Death Index" |
|  | Meyerhardt et al. (2007) | - outcome assessment the same in all groups - No information about outcome assessment |
|  | Ollberding et al. (2013) | - outcome assessment the same in all groups - "Vital status was obtained through active patient follow-up supplemented by data linkages to the Nebraska state death certificate files." |
|  | Park et al. (2022) | - outcome assessment the same in all groups - "Deaths were identified by linkage to Hawaii and California death files and the National Death Index" |
|  | Pelser et al. (2014) | - outcome assessment the same in all groups - "Mortality and cause of death were ascertained by linkage with the Social Security Administration Death Master File and the National Death Index Plus" |
|  | Ratjen et al. (2017) | - outcome assessment the same in all groups - Vital status was obtained via population registries. |
|  | Ratjen et al. (2021) | - outcome assessment the same in all groups - "Vital status of all study participants was updated from March to June 2016 via population registries" |
|  | Sharma et al. (2018) | - outcome assessment the same in all groups - "We conducted follow-up questionnaires with participants and linked records to death certificates, pathology reports, autopsy records, physicians’ notes, and surgical reports. Additional data were obtained from the Dr. H. Bliss Murphy Cancer Care Foundation; many of the results can be mutually verified" |
|  | Song et al. (2021) | - outcome assessment the same in all groups - "Deaths were identified through review of the National Death Index, postal authorities, or the next-of-kin in response to the follow-up questionnaires. The cause of death was identified by study physicians blinded to exposure data through review of death certificates and medical records." |
|  | Sun et al. (2018) | - outcome assessment the same in all groups - "All medical records were reviewed centrally by the WHI Outcomes Adjudication Physician Committee before assigning diagnosis in the data set; similar approaches were used to inform on cause of death." |
|  | Thomson et al. (2014) | - outcome assessment the same in all groups - "Death of any cause was ascertained from proxy report, or vital status was determined through linkage with the National Death Index with cause of death coded according to the International Classification of Diseases" |
|  | Van Blarigan et al. (2020) | - outcome assessment the same in all groups - No information about outcome assessment |
|  | Van Zutphen et al. (2021) | - outcome assessment the same in all groups - "Information on recurrences was collected from medical records by trained registrars from the Dutch Cancer Registry"; "Vital status and date of death were determined through linkage to the Municipal Personal Record Database of the Netherlands" |
|  | Vrieling et al. (2013) | - outcome assessment the same in all groups - "Vital status of participants was determined through population registries up to the end of 2009. Causes of death were extracted from death certificates. Medical records were checked or treating physicians were contacted to identify recurrences and to verify self-reported events collected during a follow-up telephone interview conducted from May to September 2009 (>90% self-reported events verified)." |
|  | Wang et al. (2020) | - outcome assessment the same in all groups - "Information on breast cancer recurrence/metastasis, mortality and cause of death for current study participants was collected during the 10-year postdiagnosis in-person survey, with a response rate of 93.8%. Survival status and cause of death were supplemented by periodic record linkage to the Shanghai Vital Statistics Registry" |
|  | Wen et al. (2022) | - outcome assessment the same in all groups - Outcome data were obtained by medical records and active follow-up. |
|  | Yang et al. (2015) | - outcome assessment the same in all groups - "Deaths were identified by reports from family members and postal authorities, and systematic searches of the National Death Index. Deaths were confirmed through review of death certificates and medical records to determine cause of death, assigned by the Endpoints Committee of three physicians. When medical records cannot be obtained, cause of death is assigned upon reviews of all other available data by the Endpoints Committee." |
| **Bias due to selective reporting of the results**   - Is the reported effect estimate likely to be selected from multiple analyses of exposure-outcome relationship? - Is the reported effect estimate likely to be selected from different subgroups?   Notes: In observational studies, it is unusual to publish an a priori analysis plan or protocol. Multiple outcome measurements for the definition of cancer and individual cancer stages are not expected. | Al Ramadhani et al. (2021) | - Effect estimates unlikely to be selected from multiple analyses - Unlikely to be selected from different subgroups |
|  | Anyene et al. (2021) | - Effect estimates unlikely to be selected from multiple analyses - Unlikely to be selected from different subgroups |
|  | Arthur et al. (2013) | - Effect estimates unlikely to be selected from multiple analyses - Unlikely to be selected from different subgroups |
|  | Deshmukh et al. (2018) | - Effect estimates unlikely to be selected from multiple analyses - Unlikely to be selected from different subgroups |
|  | Di Maso et al. (2020) | - Effect estimates unlikely to be selected from multiple analyses - Unlikely to be selected from different subgroups |
|  | Di Maso et al. (2021) | - Effect estimates unlikely to be selected from multiple analyses - Unlikely to be selected from different subgroups |
|  | Ergas et al. (2021) | - Effect estimates unlikely to be selected from multiple analyses - Unlikely to be selected from different subgroups |
|  | Ferronha et al. (2012) | - Effect estimates unlikely to be selected from multiple analyses - Unlikely to be selected from different subgroups |
|  | Fung et al. (2014) | - Effect estimates unlikely to be selected from multiple analyses - Unlikely to be selected from different subgroups |
|  | George et al. (2011) | - Effect estimates unlikely to be selected from multiple analyses - Unlikely to be selected from different subgroups |
|  | Guinter et al. (2018)^74^ | - Effect estimates unlikely to be selected from multiple analyses - Unlikely to be selected from different subgroups |
|  | Inoue-Choi et al. (2013 | - Effect estimates unlikely to be selected from multiple analyses - Unlikely to be selected from different subgroups |
|  | Izano et al. (2013) | - Effect estimates unlikely to be selected from multiple analyses - Unlikely to be selected from different subgroups |
|  | Jacobs et al. (2016) | - Effect estimates unlikely to be selected from multiple analyses - Unlikely to be selected from different subgroups |
|  | Karavasiloglou et al. (2019) | - Effect estimates unlikely to be selected from multiple analyses - Unlikely to be selected from different subgroups |
|  | Kenfield et al. (2014) | - Effect estimates unlikely to be selected from multiple analyses - Unlikely to be selected from different subgroups |
|  | Kim et al. (2011) | - Effect estimates unlikely to be selected from multiple analyses - Unlikely to be selected from different subgroups |
|  | Kroenke et al. (2005) | - Effect estimates unlikely to be selected from multiple analyses - Unlikely to be selected from different subgroups |
|  | Kwan et al. (2009) | - Effect estimates unlikely to be selected from multiple analyses - Unlikely to be selected from different subgroups |
|  | Lee et al. (2020) | - Effect estimates unlikely to be selected from multiple analyses - Unlikely to be selected from different subgroups |
|  | Lei et al. (2021) | - Effect estimates unlikely to be selected from multiple analyses - Unlikely to be selected from different subgroups |
|  | Luo et al. (2020) | - Effect estimates unlikely to be selected from multiple analyses - Unlikely to be selected from different subgroups |
|  | McCullough et al. (2016) | - Effect estimates unlikely to be selected from multiple analyses - Unlikely to be selected from different subgroups |
|  | Meyerhardt et al. (2007) | - Effect estimates unlikely to be selected from multiple analyses - Unlikely to be selected from different subgroups |
|  | Ollberding et al. (2013) | - Effect estimates unlikely to be selected from multiple analyses - Unlikely to be selected from different subgroups |
|  | Park et al. (2022) | - Effect estimates unlikely to be selected from multiple analyses - Unlikely to be selected from different subgroups |
|  | Pelser et al. (2014) | - Effect estimates unlikely to be selected from multiple analyses - Unlikely to be selected from different subgroups |
|  | Ratjen et al. (2017) | - Effect estimates unlikely to be selected from multiple analyses - Unlikely to be selected from different subgroups |
|  | Ratjen et al. (2021) | - Effect estimates unlikely to be selected from multiple analyses - Unlikely to be selected from different subgroups |
|  | Sharma et al. (2018) | - Effect estimates unlikely to be selected from multiple analyses - Unlikely to be selected from different subgroups |
|  | Song et al. (2021) | - Effect estimates unlikely to be selected from multiple analyses - Unlikely to be selected from different subgroups |
|  | Sun et al. (2018) | - Effect estimates unlikely to be selected from multiple analyses - Unlikely to be selected from different subgroups |
|  | Thomson et al. (2014) | - Effect estimates unlikely to be selected from multiple analyses - Unlikely to be selected from different subgroups |
|  | Van Blarigan et al. (2020) | - Effect estimates unlikely to be selected from multiple analyses - Unlikely to be selected from different subgroups |
|  | Van Zutphen et al. (2021) | - Effect estimates unlikely to be selected from multiple analyses - Unlikely to be selected from different subgroups |
|  | Vrieling et al. (2013) | - Effect estimates unlikely to be selected from multiple analyses - Unlikely to be selected from different subgroups |
|  | Wang et al. (2020) | - Effect estimates unlikely to be selected from multiple analyses - Unlikely to be selected from different subgroups |
|  | Wen et al. (2022) | - Effect estimates unlikely to be selected from multiple analyses - Unlikely to be selected from different subgroups |
|  | Yang et al. (2015) | - Effect estimates unlikely to be selected from multiple analyses - Unlikely to be selected from different subgroups |

| **Overall judgement**   - Low risk of bias - Moderate risk of bias - Serious risk of bias - No information | Al Ramadhani et al. (2021) | H |
| --- | --- | --- |
|  | Anyene et al. (2021) | H |
|  | Arthur et al. (2013) | M |
|  | Deshmukh et al. (2018) | H |
|  | Di Maso et al. (2020) | H |
|  | Di Maso et al. (2021) | H |
|  | Ergas et al. (2021) | M |
|  | Ferronha et al. (2012) | M |
|  | Fung et al. (2014) | M |
|  | George et al. (2011) | N.I. |
|  | Guinter et al. (2018) | M |
|  | Inoue-Choi et al. (2013) | H |
|  | Izano et al. (2013) | M |
|  | Jacobs et al. (2016) | M |
|  | Karavasiloglou et al. (2019) | H |
|  | Kenfield et al. (2014) | M |
|  | Kim et al. (2011) | M |
|  | Kroenke et al. (2005) | M |
|  | Kwan et al. (2009 | M |
|  | Lee et al. (2020) | H |
|  | Lei et al. (2021) | H |
|  | Luo et al. (2020) | M |
|  | McCullough et al. (2016) | M |
|  | Meyerhardt et al. (2007) | N.I. |
|  | Ollberding et al. (2013) | H |
|  | Park et al. (2022) | M |
|  | Pelser et al. (2014) | M |
|  | Ratjen et al. (2017) | H |
|  | Ratjen et al. (2021) | H |
|  | Sharma et al. (2018) | H |
|  | Song et al. (2021) | H |
|  | Sun et al. (2018) | H |
|  | Thomson et al. (2014) | H |
|  | Van Blarigan et al. (2020) | N.I. |
|  | Van Zutphen et al. (2021) | M |
|  | Vrieling et al. (2013) | M |
|  | Wang et al. (2020) | M |
|  | Wen et al. (2022) | H |
|  | Yang et al. (2015) | H |

Abbreviations: *ACS* American Cancer Society, *aMED* Alternate Mediterranean Diet Score, *CALGB* Cancer and Leukemia Group B, *DHDI* Dutch Healthy Diet Index, *FFQ* food frequency questionnaire, *H* serious risk of bias, *HPFS* Health Professionals Follow-up Study, *LACE* Life After Cancer Epidemiology, *M* moderate risk of bias, *MED* Mediterranean Diet Score, NHS, *N.I.* no information, *RR* relative risk, *WCRF/AICR* World Cancer Research Fund/ American Institute for Cancer Research.
